# The impact of isosmotic conditions on the metabolism and hypoxia tolerance of a reportedly oxyconforming teleost

**DOI:** 10.1111/jfb.70410

**Published:** 2026-03-16

**Authors:** Timothy D. Clark, Luis L. Kuchenmüller, Elizabeth C. Hoots, Maryane Gradito, Jake M. Martin

**Affiliations:** ^1^ School of Life and Environmental Sciences, Deakin University Geelong Victoria Australia; ^2^ Department of Wildlife, Fish and Environmental Studies Swedish University of Agricultural Sciences Umeå Sweden

**Keywords:** critical oxygen saturation (O_2crit_), critical oxygen tension (P_crit_), fish, *Galaxias maculatus*, osmorespiratory compromise, oxyregulation, salinity

## Abstract

Fish must manage the competing demands of ion balance and gas exchange across the gills – a physiological tension known as the osmorespiratory compromise. In dynamic estuarine environments, the osmorespiratory compromise may be exacerbated by variable salinity and periods of hypoxia that demand high respiratory work. This study examined whether acute exposure to isosmotic conditions (9 ppt) lowers aerobic metabolism and enhances hypoxia tolerance relative to fresh water (0 ppt) in the fish *Galaxias maculatus*, a species that purportedly lacks oxyregulatory capacity when faced with hypoxia. Analysis via Bayesian mixed models found no impact of salinity on routine or standard oxygen uptake rates (*Ṁ*O_2_). The majority of fish maintained their *Ṁ*O_2_ as oxygen declined to ~10% air saturation, with only 8 of 58 individuals displaying a measurable critical oxygen saturation (O_2crit_). Average O_2crit_ values were similar across treatments (25.3% in 0 ppt versus 24.3% in 9 ppt), though the small number of fish showing a clear threshold suggests that the average O_2crit_ of the species might be substantially lower. Contrary to earlier reports, our findings show that *G. maculatus* has an oxyregulatory capacity that aligns with other teleosts. The marked interindividual variability in *Ṁ*O_2_ patterns with progressive hypoxia was a feature of this study when compared with other species, adding to a growing pattern of impressive physiological plasticity in *G. maculatus*. A clearer understanding of the consequences of the osmorespiratory compromise at the whole‐animal level relies on further examinations of the interplay between salinity and oxygen across stenohaline and euryhaline species and across acute and chronic exposures.

## 1 INTRODUCTION

Shallow‐water coastal communities face some of the most volatile conditions of all aquatic organisms (Blewett et al., 2022). Temperature, oxygen and salinity can all fluctuate remarkably on short temporal scales due to heatwaves, tidal cycles and rainfall (Kaplan et al., 2003; Morrison et al., 2002; Shaw et al., 2012). Fish living in these habitats generally exhibit impressive environmental resilience, with reports of superior tolerance to hypoxia and salinity, among other factors (Mandic et al., 2009; Molina et al., 2020). Less is understood about interactions between factors and whether, for example, a change in one environmental variable may help or hinder resilience to another variable (see Rodgers et al., 2025).

For example, oxygen and salinity can covary in estuarine habitats over short temporal scales (Onabule et al., 2020). Although fishes in such environments generally exhibit high hypoxia tolerance, it is possible that salinity interacts with hypoxia tolerance due to the trade‐off between ion regulation and oxygen uptake at the gills [i.e., the osmorespiratory compromise (Wood & Eom, 2021)]. Indeed, hypoxia tolerance was influenced by salinity in the euryhaline Atlantic killifish (*Fundulus heteroclitus*), whereby fish acclimated to approximately isosmotic conditions (11 ppt salinity) for ≥4 weeks had higher hypoxia tolerance (lower critical oxygen tension [P_crit_]) than fish acclimated to 0 ppt (Giacomin et al., 2019). Hypoxia tolerance at 11 ppt also tended to be higher than at 35 ppt – suggestive of a salinity optimum – but the trends were not significant. In the closely related Gulf killifish (*Fundulus grandis*), >14 days of acclimation to 10 ppt resulted in a marginally reduced P_crit_ relative to fish held at 1 ppt (Reemeyer & Rees, 2020). Only a few other studies have examined the interactive effects of salinity on hypoxia tolerance, and we are not aware of any study that has quantified such patterns across acute temporal scales more representative of dynamic coastal habitats. However, if this pattern holds true across fish species, then it represents a mechanism by which fish could exploit salinity gradients to modulate their resilience to hypoxia.

The common jollytail or inanga (*Galaxias maculatus*; Jenyns 1842) is a widely distributed fish in the Southern Hemisphere with high ecological and economic importance (Barbee et al., 2011). The species spawns in estuarine environments, and then the young embark on a several‐month pelagic phase in the open ocean before migrating back to estuarine and riverine habitats (McDowall et al., 1994). Our recent work on *G. maculatus* has focused on understanding the roles of oxygen and energy supply in determining growth rates and reproductive potential in response to temperature, with a particular focus on testing whether these processes are limited by gill oxygen uptake capacity (Hoots et al., 2024; Skeeles & Clark, 2024; e.g., Skeeles et al., 2023).

The relevance of our work on *G. maculatus* has recently been criticised (Müller & Pauly, 2024), with claims that the species is not appropriate for testing the theory of gill‐oxygen limitation due to reports of it being an oxyconformer (Urbina et al., 2012; Urbina & Glover, 2013). That is, it has been argued that *G. maculatus* is unable to maintain resting rates of oxygen uptake once environmental oxygen declines below 100% air saturation. There are two main reasons why the oxyconformer status of *G. maculatus* raises questions. First, teleost fishes are almost exclusively oxyregulators with a detectable P_crit_ (a.k.a. O_2crit_ when measured as oxygen saturation) (Ultsch & Regan, 2019). Indeed, Ultsch and Regan (2019) and Svendsen et al. (2019) questioned the oxyconforming status of *G. maculatus*, pointing to a range of other species that were reported to be oxyconformers until subsequent studies proved otherwise. Second, the respirometry approaches used in the studies characterising *G. maculatus* as an oxyconformer had some critical limitations; specifically, the respirometers were not equipped with a mixing mechanism, and oxygen was measured only sparsely by removing water samples from the respirometers rather than logging oxygen continuously within them (Urbina et al., 2012; Urbina & Glover, 2013). These issues are known to affect data quality and have the potential to result in misleading interpretations (Clark et al., 2013; Rodgers et al., 2016).

Here, we address several of the open questions identified above. Using *G. maculatus* exposed to a salinity of either 0 ppt (fresh water) or 9 ppt [isosmotic; Urbina and Glover (2015)], we test (1) whether the species exhibits oxyregulation in the face of progressive hypoxia, and (2) whether acute exposure to isosmotic conditions can ease the osmorespiratory compromise to reduce resting oxygen uptake rates and improve hypoxia tolerance (measured as O_2crit_).

## 2 MATERIALS AND METHODS

All experiments were conducted in accordance with the guidelines set by the Deakin University Animal Ethics Committee (#B31‐2022), which complies with the Australian Code for the Care and Use of Animals for Scientific Purposes set by the Australian Federal Government.

### 2.1 Animals and holding conditions

Juvenile *G. maculatus* (*n* = 64, mass range = 0.21–1.60 g, length range = 40–70 mm) were collected in box traps from near the mouth of the Cumberland River in Lorne, VIC, Australia on 8 November 2024. The water temperature at capture ranged from 14.5 to 15.6°C, and salinity was measured at 0.1 ppt at all sections of the river where the fish were caught. Fish were transported by car to Deakin University's Queenscliff Marine and Freshwater Science Centre in an aerated 100 L tank filled with river water.

Upon arrival, the fish were split evenly across two aerated 200 L holding tanks, each maintained at 14°C (±0.4°C [actual range]) and with ~1000 L day^−1^ flow‐through of fresh water. The tanks were loosely covered with dark plastic to minimise visual disturbance without completely excluding light. Fish were fed ~2% of their tank biomass every 3 days (Otohime B1, BMAQUA, Frederickton, Australia) and were monitored daily for animal welfare. Water chemistry assessments of ammonia and nitrite were also conducted daily for each holding tank, with water flow‐through adjusted to maintain concentrations at or near 0 mg L^−1^. All fish appeared healthy during the course of the experiment. A natural diel light cycle was imposed by slowly ramping on the lights between 6:00 a.m. and 7:00 a.m., and slowly ramping them off between 7:00 p.m. and 8:00 p.m.

### 2.2 Experimental set‐up

An intermittent‐flow respirometry system was used to measure whole animal oxygen uptake rate (*Ṁ*O_2_) for each individual fish as a proxy for aerobic metabolic rate (Clark et al., 2013). The respirometry system consisted of two dark 70 L reservoir trays, each containing eight custom‐built, transparent respirometry chambers of either 58 or 105 mL volume (with one 1.6 g individual measured in a 300 mL respirometry chamber). Each respirometer was equipped with a stir‐bar beneath a perforated stage on the bottom of the chamber, and it sat above a slowly rotating magnetic disc to ensure that water was constantly mixed but without requiring fish to swim to maintain position. Each reservoir tray was connected to a 200 L sump. Water from each sump was pumped through 5 mm vinyl tubing to flush each of the eight respirometers within one reservoir tray, and the water exited the chambers through a standpipe within the lid. The excess water in each reservoir tray then drained back into the respective sump. Each sump was maintained at the desired dissolved oxygen saturation (DO) by bubbling air or nitrogen gas as needed, the latter regulated by an oxygen control system (OxyGuard Pacific, Farum, Denmark). Temperature in the sumps was controlled by circulating water through chillers (TK 2000, TECO, Ravenna, Italy; and HC‐1000A, Hailea, Guangdong, China).

Flushing of each respirometer could be stopped manually with a valve built into the 5 mm inflow line, or automatically via a digital timer to which each reservoir's flush pump was connected (Smart_shifter, LabVIEW 2012, National Instruments, Austin, TX, USA). The lid of each respirometry chamber was equipped with an optical oxygen sensor through a cable gland, which logged data to a Firesting unit (PyroScience, Aachen, Germany). Four Firesting units were run in parallel, each reading from four oxygen sensors and one temperature sensor. The Firesting units recorded oxygen concentration (mg O_2_ L^−1^) and temperature of each chamber at 0.2 Hz (i.e., every 5 s) to a laptop running Oxygen Logger software (PyroScience, Germany).

All 16 oxygen probes were initially calibrated at 0% air saturation using sodium sulphite, and before each trial all oxygen sensors were calibrated to 100% air saturation in vigorously aerated fresh water. Oxygen leak tests were performed initially and whenever adjustments were made to any chambers; this involved injecting deoxygenated water into the standpipes of the sealed chambers and monitoring their measured oxygen level relative to the water in the reservoir tray. These tests were performed with the reservoir tray maintained at 100% air saturation and also at 50% air saturation (see the following sections).

### 2.3 Respirometry protocol

Trials were conducted on all 64 fish, but isolated technical issues (e.g., oxygen sensors going offline) prevented robust data from 6 fish, leaving *n* = 58 presented henceforth. Fish were fasted for 72 h before being netted and placed into the respirometers one at a time. We alternated the holding tanks from which fish were taken to allow them to be fed on a regular schedule. Fish were allocated to either 58 or 105 mL respirometers based on visual assessment of their relative size. For each run, at least one oxygen sensor was left free‐floating in each reservoir tray to allow the continuous determination of oxygen levels in the reservoir water. Additionally, all initial trials were conducted with at least one experimental blank (i.e., a fish‐free chamber with the lid attached) for each respirometer size to quantify background (microbial) respiration. Following extensive testing, some later trials included only one chamber per tray as an experimental blank.

Once all respirometers contained fish or were designated as experimental blanks, one of the two sumps (randomised for each trial) was flushed at a rate of ~1.2 L min^−1^ with seawater until it reached a salinity of 9–10 ppt after 60–90 min, whereas the other sump received a similar flow‐through of fresh water for the same amount of time. The salinity of 9–10 ppt was chosen to reflect the isosmotic level in teleosts in general and *G. maculatus* specifically (Urbina & Glover, 2015). Flow‐through water was then ceased to both sumps. The trays were loosely covered with an opaque black plastic sheet to minimise visual disturbance, and fish were left undisturbed overnight (12–19 h). During this period of resting, a digital timer was set to intermittently flush and seal all chambers on an automated 30 min:10 min flush:seal cycle.

The following morning at approximately 8:30 a.m., for four of six trials (*n* = 39 individuals), nitrogen was gently bubbled in both sumps while chambers were flushing. The sumps (and chambers and reservoir trays) were reduced to 75% air saturation across 30 min, maintained at that level using the OxyGuard system, and all chambers were sealed with the manual valves for 60 min. Valves were then opened, and chambers were briefly flushed with water of 75% air saturation before sumps were reduced with nitrogen bubbling to 50% air saturation over a 30 min period while chambers were flushing. Again, each chamber was sealed with the manual valves (this time at 50% air saturation) and they remained sealed until oxygen levels dropped below 10% air saturation (1.031 or 0.975 mg O_2_ L^−1^ for 0 ppt and 9 ppt, respectively) or the chamber oxygen stopped decreasing (an indication of loss of equilibrium). This respirometry approach required 3–8 h (median 6 h).

For the other two of six trials (*n* = 19 individuals), at approximately 8:30 a.m. chambers were manually sealed at 100% air saturation and remained sealed until the same endpoints noted above. The sumps were gently bubbled with nitrogen to obtain 50% air saturation in the reservoir trays within 1–2 h of sealing the respirometers, to ensure the water surrounding the respirometers matched the oxygen levels used in the other four of six trials. This approach allowed us to monitor *Ṁ*O_2_ across a broader DO range, but the duration of the trials was extended to 3–13 h (median 7 h).

After all chambers were opened, total body length (mm) and mass after careful blotting (to the nearest 0.01 g) were determined for all fish. After removal of the fish, all respirometers were sealed at 100% air saturation for more than 20 min to measure background respiration. Freshwater conditions were re‐established for the next trial.

### 2.4 Data analysis and statistics

For estimates of aerobic metabolic rate, output from the Firesting system was reformatted in Microsoft Excel for import into LabChart (ADInstruments, Sydney, Australia). A slope was calculated from the oxygen measurements (mg L^−1^ s^−1^) for each sealed cycle of the intermittent‐flow phase of the trial (~7 min taken from each 10‐min slope) and for rolling 20‐min intervals for the manually sealed phases of the trial (where necessary, slightly less than 20 min was used). These slopes were corrected for any background respiration and, using the tests with deoxygenated water performed throughout the experimental period, slopes were corrected for any oxygen diffusion dynamics between the chambers (which typically dropped to <10% air saturation during O_2crit_ trials) and reservoir baths (which were maintained at 50% air saturation during O_2crit_ trials). The impact of all corrections to *Ṁ*O_2_ values is displayed in File S1, Figure S1 for transparency (File S1 contains all supplementary [S] figures and tables). The first five cycles (~3.5 h) of the intermittent‐flow phase were removed to allow for fish habituation to respirometers. Slopes estimated using <5 min intervals were removed, as were any slopes that were visually anomalous (<0.5% of slopes). The absolute value of the slopes was multiplied by the volume of the respirometry chamber (minus fish mass, assuming 1 g = 1 mL) for the corrected measure of *Ṁ*O_2_ in mg O_2_ h^−1^ (see Figures S2–S4 for *Ṁ*O_2_–DO relationships for each salinity and chamber size combination).

Data analysis was conducted in R (version 4.2.3) using the R studio environment, Build 463 (R Core Team, 2023). Every *Ṁ*O_2_ measurement during the intermittent‐flow phase of respirometry at 100% air saturation was used in routine metabolic rate (RMR) assessments (after removing the first ~3.5 h post‐entry). Standard metabolic rate (SMR) at 100% air saturation was estimated from the intermittent‐flow stage of the trial using the custom function ‘*calcSMR*’ by Claireaux and Chabot (2016). Two alternative methods were applied depending on the variability in the leftmost *Ṁ*O_2_ distribution for each fish. When the coefficient of variation for the leftmost distribution was less than 5.4% – indicating a tightly clustered group of low metabolic rates – the mean of the lowest normal distribution (MLND) was used. Otherwise, the mean of the lowest 20% quantile was used.

To evaluate the group‐level effects of salinity on SMR and RMR, Bayesian generalised linear mixed models (GLMMs) with Gamma‐distributed errors and a log link function were used. For the RMR model, all *Ṁ*O_2_ measurements from the intermittent‐flow stage of the trial were included. Fixed effects were: mean temperature during the interval of the slope estimate (13.84–14.38°C), light phase during the interval (light or dark; light defined as 07:00 a.m.–07:00 p.m.), cycle number (5–27), fish mass (0.21–1.60 g) and salinity treatment (0 or 9 ppt). A random intercept was included for each individual fish (fish ID: 1–58) to account for repeated measures. The shape parameter (α) was modelled as a function of measurement number and salinity. For SMR, the model included fixed effects for the mean temperature across cycles (13.87–14.26°C), the total number of cycles (10–23), fish mass and salinity treatment. In this case, α was modelled as a function of salinity.

To assess the relationship between mass‐specific *Ṁ*O_2_ (mg O_2_ g^−1^ h^−1^) and dissolved oxygen (DO; % air saturation), an incremental regression approach was used, adapted from Urbina et al. (2012). A series of Bayesian regression models with increasing polynomial order (from 0th to 3rd) were fit to the data. Comparing the relative fit of each model allowed a mathematical assessment of whether individuals exhibited oxyconforming or oxyregulating behaviour (Urbina et al., 2012). A linear (first‐order) relationship with a positive slope was interpreted as evidence of oxyconforming, whereas a flat (0th‐order) or higher‐order (second‐ or third‐order) polynomial relationship indicated oxyregulating behaviour. Model comparison was conducted using the expected log pointwise predictive density for leave‐one‐out cross‐validation (ELPD‐LOO; *elpd_loo* function, *LOO* package). This Bayesian model evaluation approach estimates generalisation performance by iteratively refitting the model with one data point held out. This approach penalises models that overfit the training data and perform poorly on unseen observations.

The critical oxygen saturation for aerobic metabolism (O_2crit_) was estimated for all fish using the rule‐based linear regression method of Claireaux and Chabot (2016). For this study, O_2crit_ was defined as the lowest ambient oxygen saturation at which an organism's SMR can be maintained through aerobic respiration [i.e., the minimum level of oxygen required to sustain the metabolic demands of maintenance; Chabot et al. (2016)]. Below this threshold, the organism is said to be an oxyconformer, and its oxygen uptake rate (*Ṁ*O_2_) decreases in parallel with environmental oxygen levels. This method involves identifying the lowest 5% of *Ṁ*O_2_ values recorded at normoxic DO (≥ 80%) and locating the lowest DO value at which *Ṁ*O_2_ remains above this threshold – this becomes the ‘pivot point’. Below the pivot point, *Ṁ*O_2_ values are considered to indicate regulatory failure. A linear regression is then fit to this subset; if the intercept is positive, it is forced through the origin. The O_2crit_ is the point where this regression intersects the SMR. Here, we applied a numerical‐based and visual‐based approach to determine reliable O_2crit_ estimates. For the numerical approach, only individuals best fit by higher‐order polynomial models (second or third order) with three consecutive *Ṁ*O_2_ values below the SMR and the lowest 5% of *Ṁ*O_2_ at normoxia were considered reliable. For the visual, expert‐judgement‐based approach, the *Ṁ*O_2_–DO relationship for all fish (*n* = 58) was assessed by each author independently, and the presence of an O_2crit_ was categorised into yes, no or maybe.

Bayesian models were run across four chains using default priors for 8000 iterations with 1000 warm‐up samples (for incremental regression models, 2000 iterations and 100 warm‐up samples were used). Convergence was assessed via trace plots and R‐hat statistics (values = 1 indicating convergence). Model predictions are presented as estimated marginal mean contrasts expressed as ratios (fold changes) with 95% highest posterior density credible intervals (CrIs). Evidence for an effect was inferred when the 95% CrI excluded 1.0.

## 3 RESULTS

**FIGURE 1:**
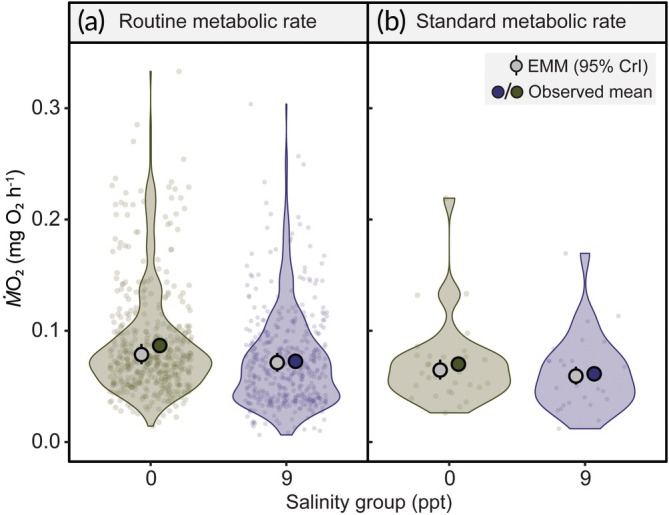
Routine and standard aerobic metabolic rates of *Galaxias maculatus* are not dependent on salinity. Routine metabolic rate (RMR; mg O_2_ h^−1^) (a) and standard metabolic rate (SMR; mg O_2_ h^−1^) (b), plotted by salinity treatment and controlling for mass and other covariates (Tables S1 and S2). Transparent points show observed values; the shaded area is a kernel density of the observed data. The large coloured points (right) show the observed means, whereas the large grey points with error bars (left) show the estimated marginal means with 95% Highest Posterior Density Credible Intervals (95% CrI). Note that there is only one SMR value per fish (*n* = 30 and 28 at 0 and 9 ppt, respectively), whereas each fish has multiple RMR values (see Materials and Methods).

Salinity treatment did not have a meaningful effect on RMR or SMR, with respective values in 0 ppt being 1.11‐fold (95% CrI: 0.94 to 1.29) and 1.09‐fold (95% CrI: 0.89 to 1.32) higher than in 9 ppt (Figure 1). Fish exhibited higher RMR during the light phase, estimated to be 1.14‐fold higher than during the dark phase (1.09–1.20). For estimates associated with all other covariates, see Tables S1 and S2.

**FIGURE 2:**
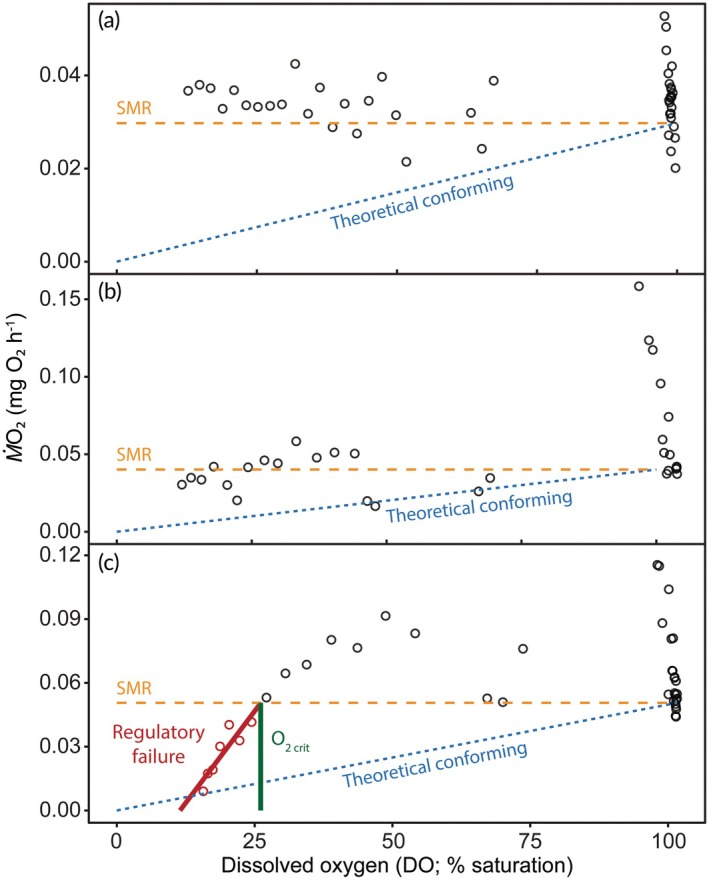
Different responses in oxygen uptake rate (*Ṁ*O_2_) of individual *Galaxias maculatus* exposed to declining dissolved oxygen. Representative examples of fish that were best modelled by a 0th‐order polynomial (a), first‐order polynomial (b) and second‐order polynomial (c). The orange dashed line shows the standard metabolic rate (SMR), whereas the blue dotted line shows the theoretical oxyconforming regression. For plot (c), the solid red sloped line shows the regression associated with failure of oxyregulation based on the rule‐based linear regression method of Claireaux and Chabot (2016), and the solid vertical green line shows the O_2crit_ estimate (intersection of oxyregulatory failure regression and SMR).

The relationship between *Ṁ*O_2_ and DO during the hypoxia tolerance tests was most frequently best predicted by a second‐order polynomial (*n* = 22; 38% of fish), followed by third‐order (*n* = 15; 26%), first‐order (*n* = 11; 19%) and zeroth‐order polynomials (*n* = 10; 17%) (Figure S5). These relationships were not driven by body mass, as there were no mass differences between the polynomial groupings (Table S3). A zeroth‐order fit suggests *Ṁ*O_2_ remained stable across the tested DO range, indicating oxyregulation without an identifiable critical oxygen threshold (O_2crit_) (Figure 2a). In contrast, first‐order relationships may suggest oxyconforming behaviour – though to qualify as evidence of oxyconformity, the regression slope must be positive and the 95% CrI must not include zero (Figure 2b). Of the 11 fish best fit by first‐order polynomials, only 3 met this criterion (see Table S4). Second‐ and third‐order polynomial fits may indicate the presence of an O_2crit_, where *Ṁ*O_2_ begins to decline after a period of oxyregulating behaviour (Figure 2c).

Among the 37 fish whose *Ṁ*O_2_–DO relationship was best modelled by a higher‐order polynomial, 16 met the numerical criteria for a potential O_2crit_ using the rule‐based regression method (*n* = 9 in 0 ppt, *n* = 7 in 9 ppt; Figure S6). These individuals had three consecutive *Ṁ*O_2_ values below both the SMR and the fifth percentile of normoxic *Ṁ*O_2_ values at the lowest recorded DO levels. From the visual‐based inspection of all *Ṁ*O_2_–DO relationships (i.e., all 58 fish), 10 fish were determined to have an O_2crit_ based on the majority decision from independent assessments of each author (see Figure S7 for a list of assessments and alignment among authors). A total of eight fish met both the numerical and visual criteria (*n* = 5 in 0 ppt, *n* = 3 in 9 ppt; shown in Figures S8 and S9). The mean values (and ranges) were 25.3% air saturation (19.1%–32.0% air saturation) in 0 ppt and 24.3% air saturation (21.7%–28.9% air saturation) in 9 ppt. Because so few fish exhibited any oxyconforming pattern in *Ṁ*O_2_ – despite most being taken to DO under 10% air saturation – we did not perform a statistical comparison of O_2crit_ between the two salinity groups. For all eight fish combined, the mean estimated O_2crit_ was 24.9% air saturation. In the fish where an O_2crit_ was not detected (i.e., no evidence of oxyconforming even in severe hypoxia), the lowest DO at which *Ṁ*O_2_ was measured (i.e., the lowest mean DO over a 20‐min interval) was 13.11% ± 3.23% (mean ± SD), with 8.8% being the lowest observed value. Therefore, the true among‐individual O_2crit_ range for *G. maculatus* is likely between <8.8% and 32%.

## 4 DISCUSSION

A notable feature of this study was the interindividual variability in *Ṁ*O_2_ responses in the face of declining oxygen, which was higher than in other species we have studied previously (e.g., Bowden et al., 2022; Collins et al., 2016). Such variability for *G. maculatus* appears to exist across the board, with interindividual differences in growth rate and size at maturity being extreme (Hoots et al., 2024; Skeeles & Clark, 2023). Despite the variability in *Ṁ*O_2_ responses, and in contrast with previous reports (Urbina et al., 2012; Urbina & Glover, 2013), it was clear that *G. maculatus* had a level of metabolic regulation and hypoxia tolerance comparable to other teleosts (Rogers et al., 2016). Indeed, although an O_2crit_ averaging 24.9% air saturation was detectable in 14% of individuals (8 out of 58), the vast majority of individuals did not have a detectable O_2crit_ because they maintained *Ṁ*O_2_ down to the lowest oxygen levels tested (~10% air saturation). Below, we discuss the probability for the existence of oxyconformity across fish species and then explore the osmorespiratory compromise in the context of altered oxygen demand.

### 4.1 The potential for oxyconformity in fishes

Reports of oxyconformity in fishes have existed for decades, and they have sparked enthusiastic discussions (Svendsen et al., 2019; Ultsch et al., 1981; Ultsch & Regan, 2019). Almost invariably, species that were once thought to be oxyconformers have proven to be oxyregulators upon deeper examination (Subrahmanyam, 1980; Ultsch & Regan, 2019; Virani & Rees, 2000). The reasons for the inconsistencies are likely to be diverse, but in most cases methodological artefacts rather than biological phenomena are likely to be responsible. In the case of *G. maculatus*, previous studies have used respirometry techniques that are known to yield unreliable data. For example, measuring oxygen from water samples taken sporadically from the respirometer causes two major issues: (1) slopes to calculate *Ṁ*O_2_ rely on very few data points, therefore lacking resolution and preventing contextualisation of the *Ṁ*O_2_ values relative to standard and maximum *Ṁ*O_2_ (see figure 2 in Clark et al., 2013) and (2) repeatedly disturbing fish to sample respirometer water causes elevated oxygen uptake rates and requires replacement of the removed water. Moreover, respirometers lacking a mixing mechanism typically result in heterogenous oxygen levels throughout the respirometer and thus unreliable slopes from which to calculate *Ṁ*O_2_ (see figure 4 in Clark et al., 2013; Rodgers et al., 2016).

**FIGURE 3:**
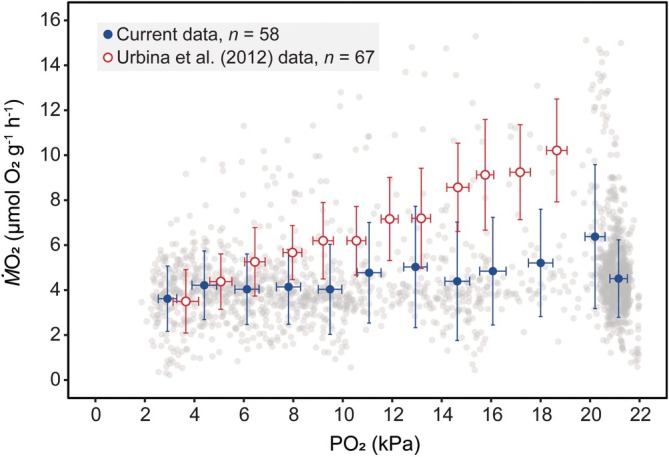
Across‐study comparison of aerobic metabolism of *Galaxias maculatus* as a function of dissolved oxygen tension. Mean and standard error of oxygen uptake rate (*Ṁ*O_2_, μmol O_2_ g^−1^ h^−1^) plotted against ambient oxygen partial pressure (PO_2_, kPa), calculated using 12 evenly spaced bins across the range of observed PO_2_ values in the current study (closed blue circles, *n* = 58). These are compared with binned mean and standard error values reported by Urbina et al. (2012) (open red circles, *n* = 67). Closed grey points represent all *Ṁ*O_2_ observations from the present study (raw values from Urbina et al. (2012) were not available). The comparison illustrates differences in the shape and magnitude of the *Ṁ*O_2_–PO_2_ relationship between studies.

To further investigate our hypoxia tolerance data in the context of what has been proposed previously, we aligned our *Ṁ*O_2_ units (μmol O_2_ g^−1^ h^−1^) and dissolved oxygen units (PO_2_ in kPa) with those used in Urbina et al. (2012) to enable a direct comparison (Figure 3). Data from Urbina et al. (2012) were obtained from their figure 1a using the metaDigitise package in R (Pick et al., 2019). This comparison shows that the resting/routine *Ṁ*O_2_ values reported for *G. maculatus* in normoxia (>18 kPa) at 14°C in previous studies (including Urbina & Glover, 2015) are twice as high as those measured here under the same conditions and in our previous research at 15°C (Skeeles et al., 2022; Skeeles & Clark, 2024). The apparent oxyconforming response to hypoxia in Urbina et al. (2012) seems to be a habituation/settling response to the respirometry protocols, or an experimental artefact resulting from repeated withdrawal of respirometer water, as the *Ṁ*O_2_ values trend down with progressive hypoxia to intersect with the values obtained across the PO_2_ range in the present study (Figure 3). Notably, the potential for cutaneous respiration in *G. maculatus* (Urbina et al., 2012) should not influence the observed patterns between whole‐animal *Ṁ*O_2_ and water PO_2_.

In sum, our results show that the criticism of our previous work on *G. maculatus* is unfounded (Müller & Pauly, 2024), adding to a growing consensus that oxyconforming teleosts do not exist (Svendsen et al., 2019; Ultsch & Regan, 2019). The agnathans (hagfish, lamprey) are thought to be oxyconformers due to their primitive physiology and gill morphology, but even that has been questioned (Perry et al., 2009).

### 4.2 Osmorespiratory compromise and its role in hypoxia tolerance

Fishes in fresh water must maintain their internal osmolality (~9–10 ppt) by actively absorbing ions across the gills and excreting dilute urine (Kültz, 2015). Adjustments in gill ventilation and perfusion to satisfy oxygen demands may inadvertently alter ion exchange in a phenomenon known as the osmorespiratory compromise. Although the mechanisms of osmorespiratory compromise remain incompletely understood (see Wood & Eom, 2021), it seems intuitive that a maintenance of internal homeostasis will be less challenging if the osmolality of the external environment matches the osmolality of the tissues. Contrary to expectations, the present study detected no differences in SMR or RMR between the two groups of fish held in 0 or 9 ppt, suggesting that any energetic benefit of being maintained in isosmotic conditions was not detectable at the whole animal level. This conclusion is supported by the majority of previous research in this field, whereby there is no clear pattern of resting *Ṁ*O_2_ being lowest under isosmotic conditions (Bœuf & Payan, 2001; Claireaux & Lagardere, 1999; Ern et al., 2014; Urbina & Glover, 2015) despite some evidence for reduced gill Na^+^/K^+^‐ATPase activities under isosmotic conditions being indicative of lowered energy requirements in coho salmon (*Oncorhynchus kisutch*) (Morgan & Iwama, 1998). On the contrary, it has been reported for salmonids following salinity acclimation that *Ṁ*O_2_ rises as salinity increases from 0 ppt to isosmotic conditions of ~9 ppt (Morgan & Iwama, 1991).

A novel aspect of the present study is that fish were transitioned to isosmotic conditions while they were in respirometers, rather than the more typical approach of acclimating fish to treatment salinities for multiple days in holding tanks before *Ṁ*O_2_ measurements [see Ern et al. (2014)]. This approach should maximise the probability of detecting any short‐term changes in *Ṁ*O_2_ associated with adjustments in gill Na^+^/K^+^‐ATPase activity and protein abundance, which are thought to take place within 3–12 h of exposure to a new salinity (Lin et al., 2006). Thus, our dataset adds to an emerging pattern of resting *Ṁ*O_2_ being largely unaffected by salinity changes within the tolerable range, although it should be noted that *G. maculatus* is a euryhaline fish with a level of environmental resilience that may exceed many other species.

It may be expected that any elevation in respiratory work – such as that required under severe hypoxia – will intensify the osmorespiratory compromise and magnify any differences between salinity treatments. Because so few of the animals in this study exhibited any oxyconforming patterns in *Ṁ*O_2_ (only 8 out of 58 fish had a detectable O_2crit_), despite being exposed to DO levels of ~10% air saturation, we cannot confidently assess differences in O_2crit_ across the two salinity treatments. Nevertheless, we can conclude that there were no obvious numerical differences in O_2crit_ between animals tested in 0 ppt vs. 9 ppt, and thus no discernible salinity‐induced changes in the osmorespiratory compromise. We are aware of only a handful of previous studies that have examined the interaction between water salinity and hypoxia tolerance. In a study of *F. heteroclitus*, there was evidence that ≥4 weeks of acclimation to 11 ppt significantly increased hypoxia tolerance (increased time to loss of equilibrium in ~3% air saturation, and decreased P_crit_) compared with fish acclimated to 0 ppt (Giacomin et al., 2019). The same directional change in P_crit_ was observed in *F. grandis* following >14 days of acclimation to 10 ppt in comparison with fish acclimated to 1 ppt (Reemeyer & Rees, 2020). No change in P_crit_ was detected in sheepshead minnow (*Cyprinodon variegatus*) following >14 days of acclimation to a broad range of salinities encompassing 0–40 ppt (~10 ppt increments), with P_crit_ only increasing once salinities of 40 ppt were exceeded (Haney & Nordlie, 1997). Finally, hypoxia tolerance (determined via loss of equilibrium upon exposure to 15% air saturation) did not change in *O. kisutch* following 120 days of acclimation to salinities ranging from 2.5 to 30 ppt (Fang et al., 2019). With such scant data available, generalisable conclusions remain elusive regarding the interactions between salinity and hypoxia tolerance, but an obvious area for attention is the role of exposure duration (acclimation *vs*. acute changes that characterise many estuaries) in governing the response across stenohaline and euryhaline species.

## 5 CONCLUSIONS

We have shown that *G. maculatus* has a capacity for oxyregulation that rivals most other teleosts, although the interindividual variation was much more dramatic than we have observed in other species (Bowden et al., 2022; Collins et al., 2016). Despite decades of research investigating aspects of the osmorespiratory compromise (Wood & Eom, 2021), the physiological mechanisms remain poorly understood and the role of hypoxia in the phenomenon has received little attention. We hope that the approaches and findings outlined here stimulate further research on these topics, including the mediating roles of temperature and body size.

## AUTHOR CONTRIBUTIONS

Timothy D. Clark: Conceptualisation, methodology, investigation, resources, data curation, writing – original draft, writing – review and editing, funding acquisition. Luis L. Kuchenmüller: Methodology, investigation, writing – review and editing. Elizabeth C. Hoots: Methodology, investigation, writing – review and editing. Maryane Gradito: Methodology, investigation, writing – review and editing. Jake M. Martin: Methodology, investigation, formal analysis, visualisation, data curation, writing – review and editing.

## FUNDING INFORMATION

This project was supported by Deakin University, including the Marine Research and Innovation Centre. L. L. K., E. C. H. and M. G. received Deakin University Postgraduate Research Scholarships, and M. G. was also supported by The Fonds de recherche du Quebec – Nature et technologies doctoral scholarships. J. M. M. received an Alfred Deakin Postdoctoral Research Fellowship.

## CONFLICT OF INTEREST STATEMENT

None of the authors have a conflict of interest to disclose.

## Supporting information


**File S1.** Supplementary Information.

## Data Availability

File S1 contains all supplementary information and can be found via the Open Science Framework at: https://doi.org/10.17605/OSF.IO/GFXCA.
