# Supplementary material for: The impact of isosmotic conditions on the metabolism and hypoxia tolerance of a reportedly oxyconforming teleost
Source: J Fish Biol. 2026 Mar 16;109(1):201–9. doi: 10.1111/jfb.70410 (PMC13397184; doi:10.1111/jfb.70410)
Supplement: Supplementary file 1 — File S1. Supplementary Information. [file JFB-109-201-s001.pdf]

The impact of isosmotic conditions on the metabolism and hypoxia tolerance of a reportedly oxyconforming teleost

Timothy D. Clark, Luis L. Kuchenmüller, Elizabeth C. Hoots, Maryane Gradito, Jake M. Martin

17 February 2026

Table of Contents

- READ ME ... 3
- Contact ... 3
- Sharing/accessing and citing ... 4
- Set up: Packages and knit settings ... 4
- Custom functions ... 5
- Directories ... 6
  - Input ... 6
  - Output ... 6
- Data ... 6
  - Slopes ($\dot{M}O$) ... 6
  - Fish metadata ... 6
  - Urbina, Glover, and Forster (2012) ... 7
  - O_2crit visual inspection ... 7
    - Combinding metadata ... 7
- Data tidy ... 7
  - Numbers ... 7
  - Fish size ... 7
  - Filtering trials ... 7
  - Filtering MO_2 estimates ... 8
  - Calculating SMR ... 9
  - Transforming MO_2 ... 9
  - Visualising MO2 corrections ... 9
    - Figure S1 ... 10
- Exploratory Data Analysis (EDA) ... 17
  - O_2 vs MO_2 ... 17

Figure S2 ... 17
Figure S3 ... 18
Figure S4 ... 18
Routine MO_2 ... 19
SMR ... 20
Individual O_2, MO_2, and SMR ... 21
🧮 Analysis ... 21
Routine MO2 ... 21
Scaling predictors ... 21
Model structure ... 22
Prior selection ... 22
Run model ... 22
Model diagnostics ... 23
📈 Results ... 26
SMR ... 28
Formating and scaling data ... 28
Model structure ... 29
Prior selection ... 29
Run model ... 29
Model diagnostics ... 29
📈 Results ... 31
Incremental regression analyses ... 33
Building Bayesian regressions ... 33
Model fits ... 34
Model predictions ... 34
📈 Results ... 35
Table S3 ... 38
Table S4 ... 38
O_2crit model ... 40
Ploting O2crit ... 41
O_2crit numerical rules ... 46
Visual inspection ... 49
O_2crit numbers ... 51

Figure S8 52
Figure S9 53
Figure 2 54
Comapring to past data 56
Figure 3 56
References 57
Session information 57

READ ME

SUMMARY This R code estimates the relationship between oxygen consumption (MO_2) and dissolved oxygen saturation (DO) in the Common Galaxias (*Galaxias maculatus*). It also estimates the critical oxygen saturation value for aerobic metabolism O2crit, commonly defined as the threshold below which the oxygen consumption rate can no longer be sustained. The associated article is titled *"The role of osmorespiratory compromise in metabolism and hypoxia tolerance of a purportedly oxyconforming teleost."* If you are reading the HTML version of this script, click the `Code` button in the top right to download the `.Rmd` file.

AIM The article aims to test whether *Galaxias maculatus* can maintain oxygen consumption (MO_2) as ambient DO declines and, if so, at what level it reaches the critical oxygen saturation value for aerobic metabolism (O2crit).

AUTHORS Timothy D. Clark [a] Luis L. Kuchenmüller [a] Elizabeth C. Hoots [a] Maryane Gradito [a] Jake M. Martin [a,b]

AFFILIATIONS [a] School of Life and Environmental Sciences, Deakin University, Geelong, VIC, Australia
[b] Department of Wildlife, Fish and Environmental Studies, Swedish University of Agricultural Sciences, Umeå, Sweden

DISCLAIMER I (Jake Martin) am dyslexic. I have made an effort to review the script for grammatical errors, but some will likely remain. I apologise. Please feel free to reach out using the contact details below if anything is unclear.

Contact

Jake M. Martin

📧 **Email:** jake.martin@deakin.edu.au

📧 **Alt Email:** jake.martin.research@gmail.com

🌐 **Web:** jakemartin.org

🐙 **GitHub**: JakeMartinResearch

📝 Sharing/accessing and citing

1. **Licenses/restrictions placed on the data:** CC-BY 4.0

2. **Link to the associated publication:**
   🚧 ***To be added*** 🚧

3. **Recommended citation for this data:**

Clark, T. D., Kuchenmüller, L. L., Hoots, E. C., Gradito, M., & Martin, J. M. (2025, May 28). The impact of isosmotic conditions on the metabolism and hypoxia tolerance of a reportedly oxyconforming teleost. https://doi.org/10.17605/OSF.IO/GFXCA

🔧 Set up: Packages and knit settings

A list of the required packages to run the script.

```
# ----- Packages ------
if (!requireNamespace("pacman", quietly = TRUE)) install.packages("pacman")

# ---- List of required packages ----
pkgs <- c(
  # ----- Data Visualisation -----
  "ggthemes", "bayesplot", "gt", "gtsummary", "plotly", "qqplotr",
"gridExtra", "flextable",

  # ----- Tidy Data and Wrangling -----
  "tidyverse", "janitor", "readxl", "broom.mixed", "data.table", "hms",
"devtools",
  "mclust", "officer", "here",

  # ----- Modelling and Statistical Analysis -----
  "brms", "rstan", "marginaleffects", "performance", "emmeans",
  "tidybayes", "respirometry", "future"
)

# ---- Install and load all packages using pacman ----
suppressPackageStartupMessages(
  pacman::p_load(char = pkgs, install = TRUE)
```

```
)

#-------kniter seetting-----------

knitr::opts_chunk$set(
  echo = FALSE, # only for PDF knit
  message = FALSE,
  warning = FALSE, # no warnings
  cache = TRUE,# cacheing to save time when kniting
  tidy = TRUE,
  fig.align = "center"
)
```

Custom functions

Here are some custom functions used within this script.

`bayes_incremental_regression_by_id()`: A custom function to build Bayesian incremental regressions. It is designed to run a list of subgroup models (IDs) in parallel using 4 cores. The function uses `brm()` with a Gaussian error distribution.

Use:
The function accepts the following arguments:
- `id_i`: A grouping factor or ID used to filter the data for each regression. If none is provided, the function uses the entire dataset.
- `id_name`: The column name (as a character string) corresponding to the grouping factor in the data frame. If not provided, the function uses all data.
- `data`: The data frame containing the variables for analysis.
- `predictor`: The predictor variable of interest.
- `response`: The response variable of interest.
- `seed_number`: A random seed value for model reproducibility.
- `save_models`: A logical argument indicating whether to save the model outputs (`TRUE` or `FALSE`).
- `mod_output_wd`: The output directory where model `.rds` files should be saved (used only if `save_models = TRUE`).

`load_rds9()`: A custom function to load all rds models in a directory and store in a list. Takes a directory with `.rds` files

`incremental_regression_bayes_fits()`: A custom function for pulling model fits, loo and r2 using `loo()` and `bayes_R2()`, respectively. Takes a list of `brm` models.

`bayes_mod_predictions()`: This function extracts the predicted values using `fitted()` from a list of models and combines them with the original data file used for the model. These are the **posterior mean fitted values** (i.e. the expected value of the response variable given the predictor variables and the estimated posterior distributions of the parameters) for each observation in the dataset, along with **95% credible intervals**.

`calcSMR()`: **authored by Denis Chabot** used to estimate SMR with several different methods Claireaux and Chabot (2016) [1]

[1] Claireaux, G. and Chabot, D. (2016) Responses by fishes to environmental hypoxia: integration through Fry's concept of aerobic metabolic scope. Journal of Fish Biology https://doi.org/10.1111/jfb.12833

`calcO2crit()`: **authored by Denis Chabot** used to estimate O2crit (Pcript). Claireaux and Chabot (2016) [1]

Note: O2 is assumed to be in percentage of dissolved oxygen (DO)

`plotO2crit()`: **authored by Denis Chabot**, used to plot the modes used for the `calcO2crit()` function. Claireaux and Chabot (2016) [1]

Note: I added abline(h=lowestMO2, col="pink") so that I could visualise the lowestMO2 position

Directories

Input

input_data_wd: Directory for the metadata

mod_data_wd: Directory for model output data estimated slopes

Output

output_fig_wd: this is where we will put the figures

output_mods_wd: this is where we will put the models

Data

Slopes (ṀO)

slope_df: We have imported the slopes extracted in LabChart during each phase of the experiment

Fish metadata

metadata: This is the meta data for each chamber

Note: We are also adding volume based on chamber type.

Urbina, Glover, and Forster (2012)

💿 **urbina_et_al_2012**: This is the mean level data extracted from Urbina, Glover, and Forster (2012)[2] Figure 1a. We used the metaDigitise package in R to extract the data [3].

O_2crit viusal inspection

💿 **o2crit_check**: This data frame includes the visual based assessment for O_2crit for all fish included in the analysis (n = 58). The inspections were made using figures genbrated by the `plotO2crit()` function, and can be viewed in `combined_chabot_plots.pdf`. The visual assessment was done independently by all authors, and the presence of an O_2crits allocated to 'yes', 'no', 'maybe'. If yes, or maybe, an estimate of the dissolved oxygen percentage was given.

Directory to the figures

📁 gmac-mo2-24/ └── 📁 output-fig/ └── 📁 model_chabot/ └── 📄 combined_chabot_plots.pdf

Combinding metadata

Adding the meta data to the slopes data frame

Data tidy

Numbers

We have **64 fish** with MO_2 data

```
## [1] "n = 64"
```

With 48 from the 0 ppt and 48 from 9 ppt groups

Salinity group	n total
0	33
9	31

Fish size

Here we calculate the mean length and size of fish used in the experiment.

```
## [1] "The mean mass of fish was 0.532 g (range: 0.21-1.6), and the mean
length was 50.41 mm (range: 40-70)"
```

Filtering trials

We will remove 6 trials which had errors. These are as follows:

- a_0_25nov_3 fish died during trial

- b_0_26nov_4 flat lined early
- c_0_22nov_2 chamber was opened early
- c_9_26nov_2 chamber was opened early
- c_9_26nov_4 chamber was opened early
- d_9_27nov_3 sensor was jumpy and end points were hard to confidently ID visually

We now have **58 fish** with MO2 data

```
## [1] "n = 58"
```

With 30 in the 0 ppt group and 28 in the 9 ppt group

Salinity group	n total
0	30
9	28

Mass and size of fish after removing 6 trials which had errors

```
## [1] "The mean mass of fish was 0.546 g (range: 0.21-1.6), and the mean
length was 50.9 mm (range: 40-70)"
```

Chamber size breakdown

Chamber type	n total	Median mass	Standard deviation mass
0.058	36	0.43	0.1496554
0.105	21	0.61	0.2561871
0.300	1	1.60	0.0000000

Numbers per trial approach

Type of trial	n total
100c	19
50c	39

Filtering MO_2 estimates

Here we apply the following filters to the MO_2 data:

- Remove the first 5 SMR cycles (burn in)
- Remove all positive raw slopes
- Remove all MO_2 calculated using less then 60 data points (5 min at 0.2 Hz)
- Remove all MO_2 calculated if O_2 increases in a closed phase (i.e. trial has ended)

Check positive values for MO_2 before removing.

```
## [1] "There are 13 fish with postive slopes. These fish are: a_0_24nov_1,
b_0_24nov_3, b_9_21nov_2, b_9_21nov_3, b_9_22nov_1, b_9_22nov_2, b_9_22nov_3,
c_0_21nov_1, c_0_21nov_2, c_0_22nov_4, c_9_24nov_3, d_0_22nov_2, d_9_25nov_3.
For all estimated slopes (n = 1105) 1.27% were postive (n = 14)"
```

Filtering the MO_2 data

Calculating SMR

We will calculate SMR using `calcSMR` function by Chabot, Steffensen and Farrell (2016)[1]. Specifically, we use mean of the lowest normal distribution (MLND) where CVmlnd < 5.4 and the mean of the lower 20% quantile (q0.2) were CVmlnd > 5.4. If CVmlnd is not calculated we have used q0.2.

Transforming MO_2

Here we are transforming the MO_2 units. The resulting values are as follows:

- **MO2** is absolute value of the background and leak corrected MO_2 slope from Labchart (mo2corr) times the net volume of the chamber (volume - fish mass), × 60, × 60, to achieve MO_2 as mg^{-1} O_2 h^{-1}
- **MO2_g** is MO2 divided by fish mass to achieve MO_2 as mg^{-1} O_2 g^{-1} h^{-1} (i.e. mass standardised)
- **SMR** absolute value of the SMR estimates using methods described by Chabot, Steffensen and Farrell (2016)[1] times the net volume of the chamber (volume - fish mass), × 60, × 60, to achieve SMR as mg^{-1} O_2 h^{-1})
- **SMR_g** is SMR divided by fish mass to achieve SMR as mg^{-1} O_2 g^{-1} h^{-1} (i.e. mass standardised)
- **DO** is dissolved oxygen percentage calculated from O_2 values (mg^{-1} L^{-1}) using the recorded temperature, salinity, and a constant atmospheric pressure (Pa; 1013.25)
- **o2_kpa** is the O_2 concentration in kilopascal (kpa). This is used to make a comparative figure only.

Trial lengths

Approach	Minimum duration	Maximum duration	Median duration
Closed at 100% air saturation	3	13	7
Closed at 75% and 50% air saturation	2.91	8.02	5.96

Visualising MO2 corrections

Let's check how data corrections change the appearance of out data MO2

Let's compare raw MO2 calculations (mo2), MO2 calculations after correcting background respiration (mo2_bground), and MO2 calculations after correcting for background respiration and oxygen diffusion dynamics (mo2corr). First we will need to make the mo2_bground vaule as it's not present in the datafile, and transform the data to long formate, so we can plot it more easly.

Figure S1

Now let's plot them in the same graph for each fish: raw MO_2 calculations are shown in pink, MO_2 calculations after correcting for background respiration are shown in grey, and MO_2 calculations after correcting for background respiration and oxygen diffusion dynamics are shown in green.

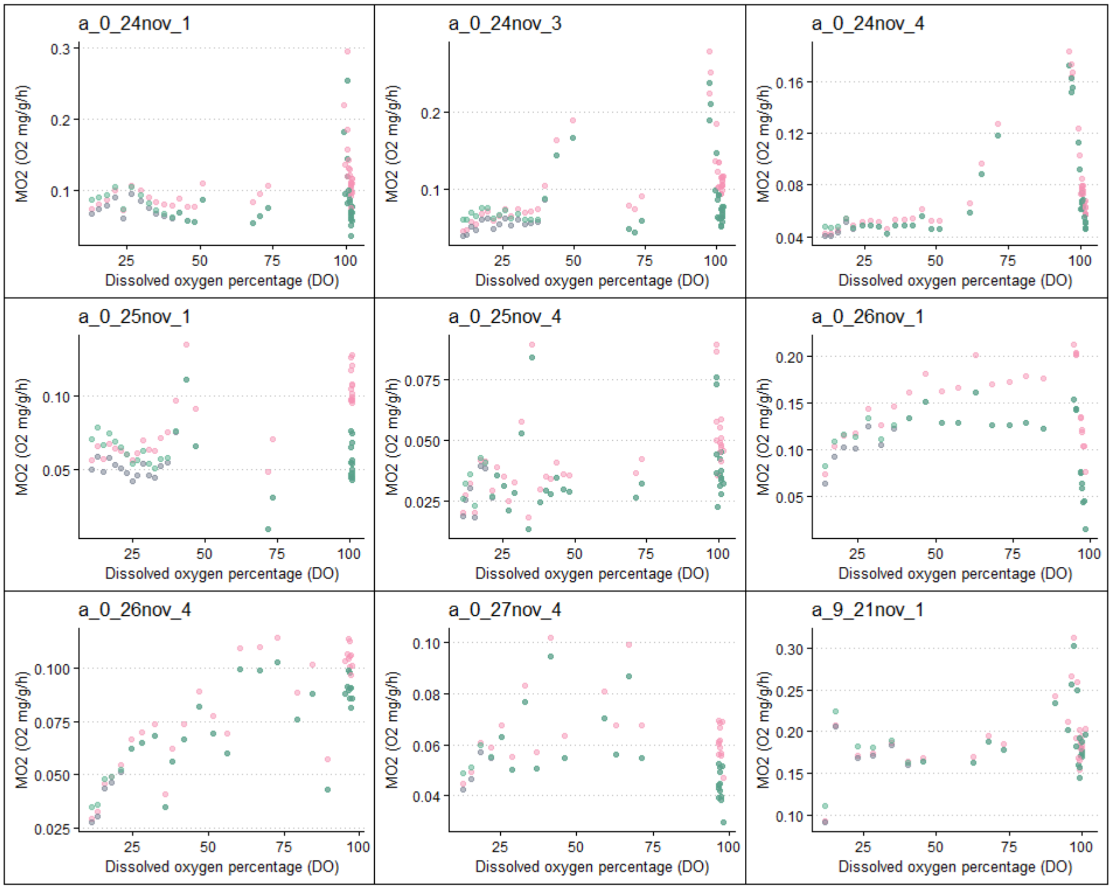

a_0_24nov_1
a_0_24nov_3
a_0_24nov_4
a_0_25nov_1
a_0_25nov_4
a_0_26nov_1
a_0_26nov_4
a_0_27nov_4
a_9_21nov_1
MO2 (O2 mg/g/h)
Dissolved oxygen percentage (DO)

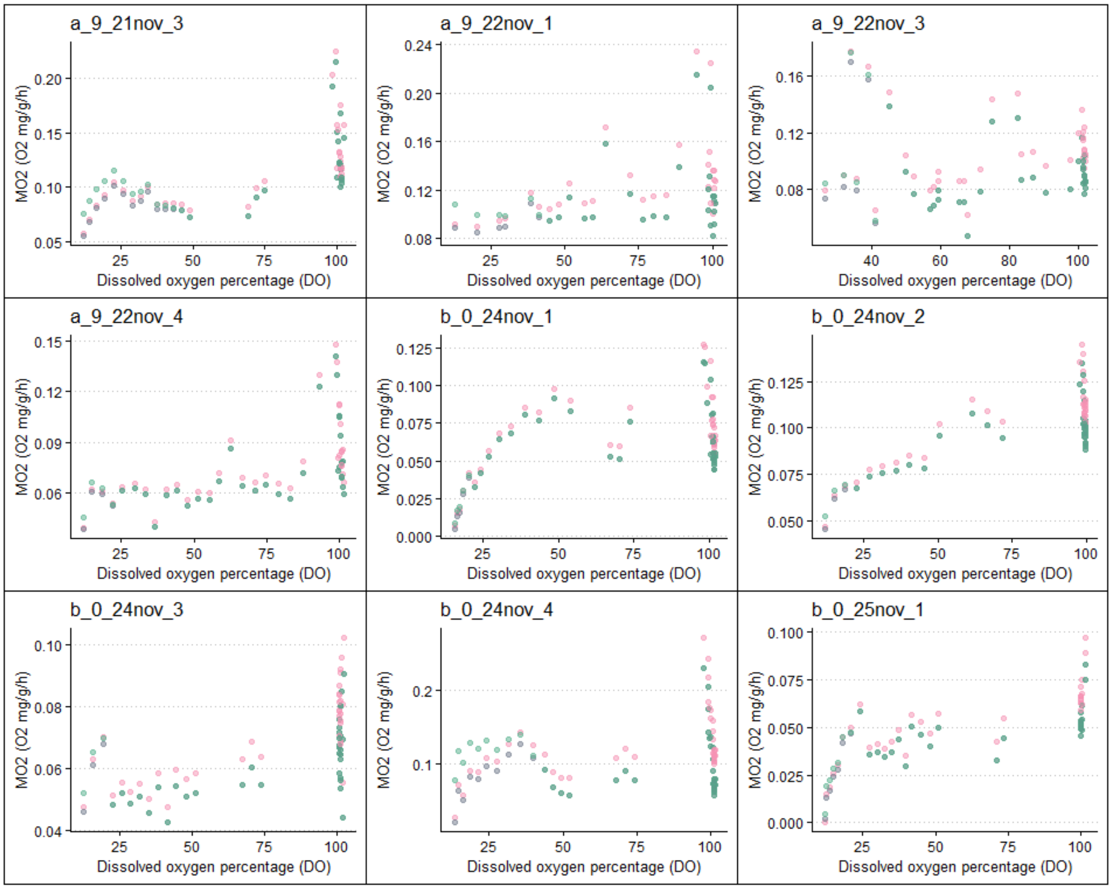
a_9_21nov_3
a_9_22nov_1
a_9_22nov_3
a_9_22nov_4
b_0_24nov_1
b_0_24nov_2
b_0_24nov_3
b_0_24nov_4
b_0_25nov_1
MO2 (O2 mg/g/h)
Dissolved oxygen percentage (DO)

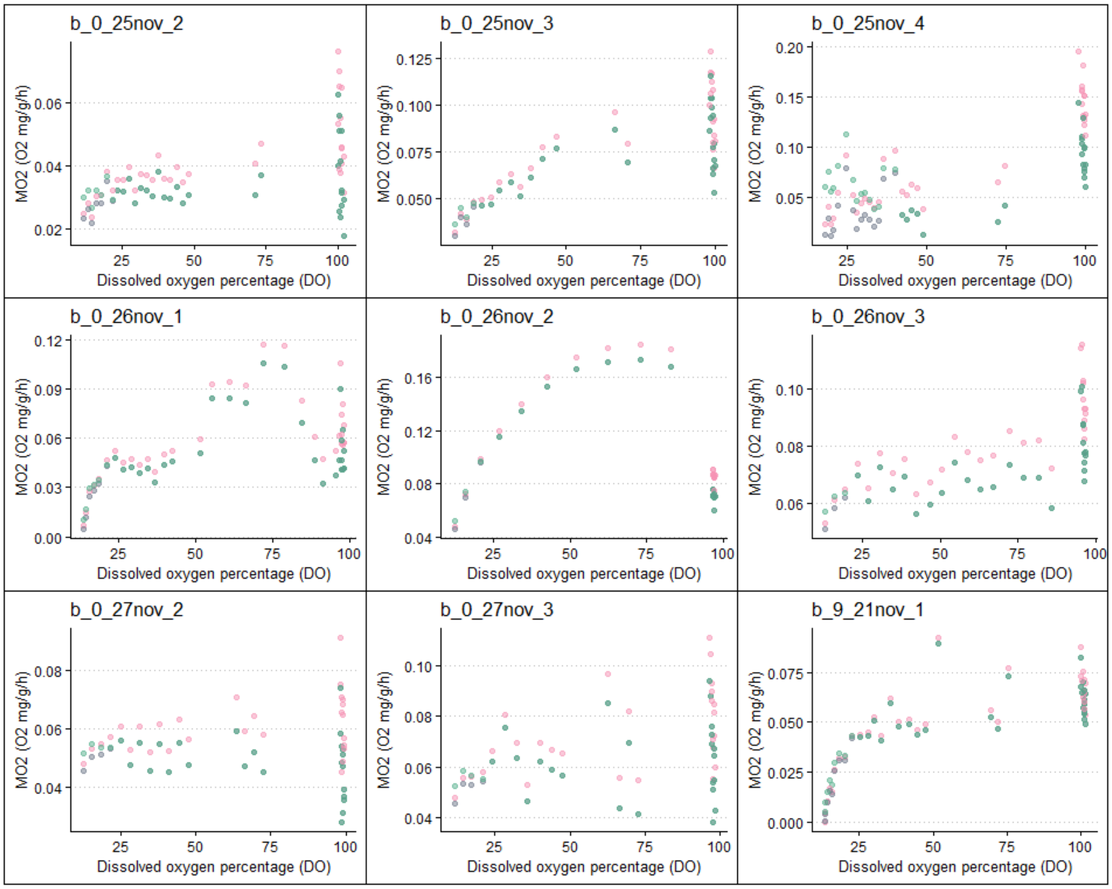

b_0_25nov_2
b_0_25nov_3
b_0_25nov_4
b_0_26nov_1
b_0_26nov_2
b_0_26nov_3
b_0_27nov_2
b_0_27nov_3
b_9_21nov_1
MO2 (O2 mg/g/h)
Dissolved oxygen percentage (DO)

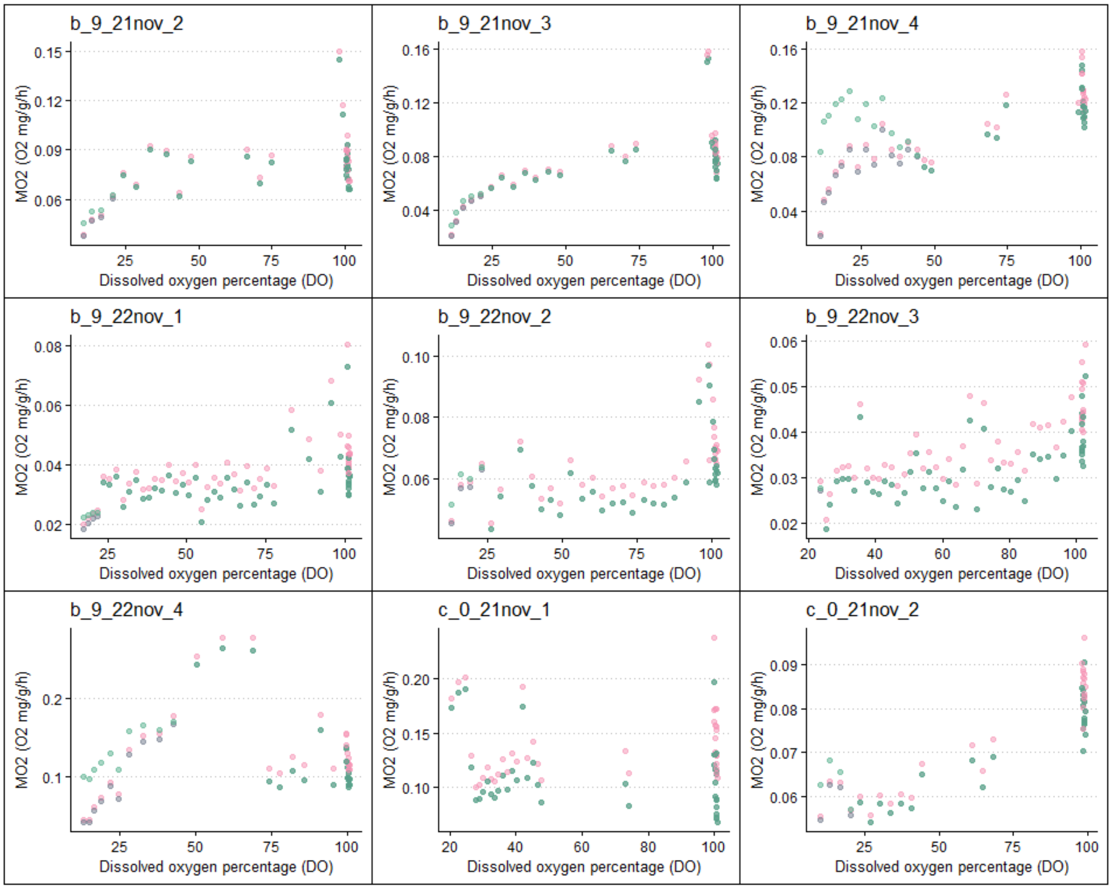
b_9_21nov_2
b_9_21nov_3
b_9_21nov_4
b_9_22nov_1
b_9_22nov_2
b_9_22nov_3
b_9_22nov_4
c_0_21nov_1
c_0_21nov_2
MO2 (O2 mg/g/h)
Dissolved oxygen percentage (DO)

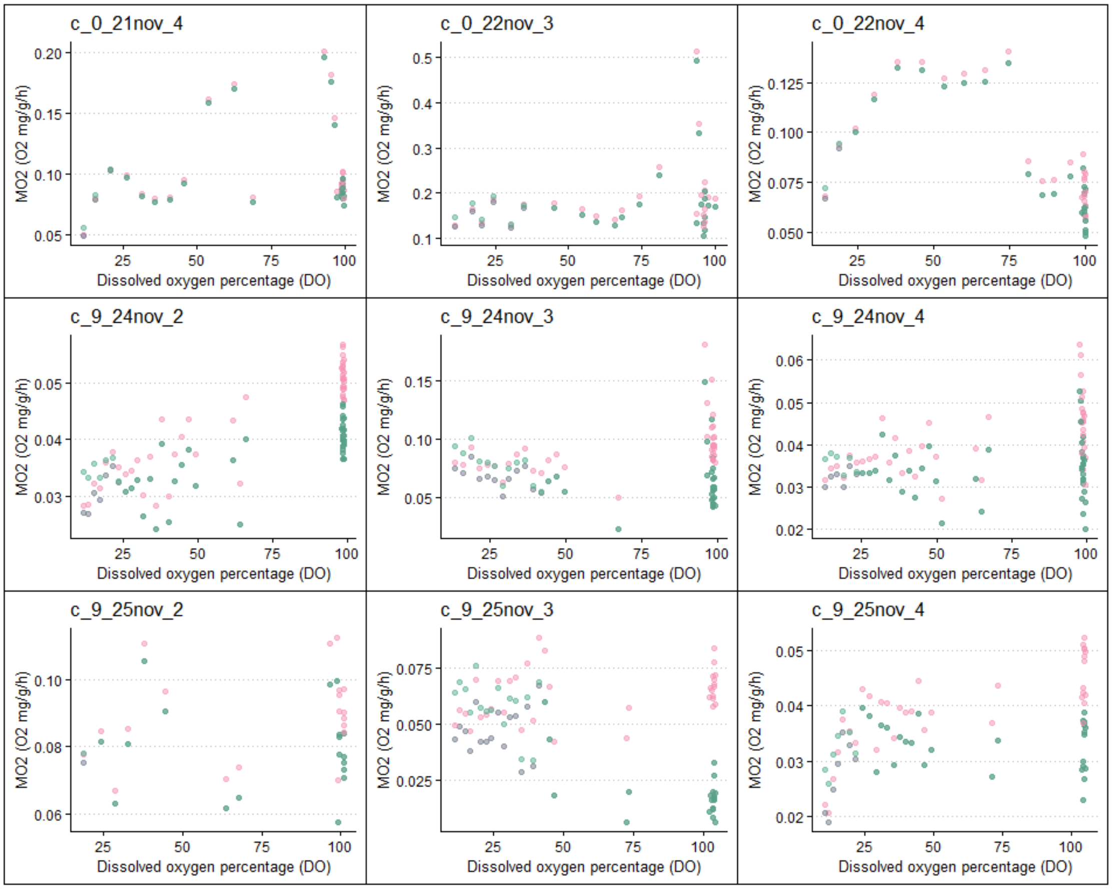
c_0_21nov_4
c_0_22nov_3
c_0_22nov_4
c_9_24nov_2
c_9_24nov_3
c_9_24nov_4
c_9_25nov_2
c_9_25nov_3
c_9_25nov_4
MO2 (O2 mg/g/h)
Dissolved oxygen percentage (DO)

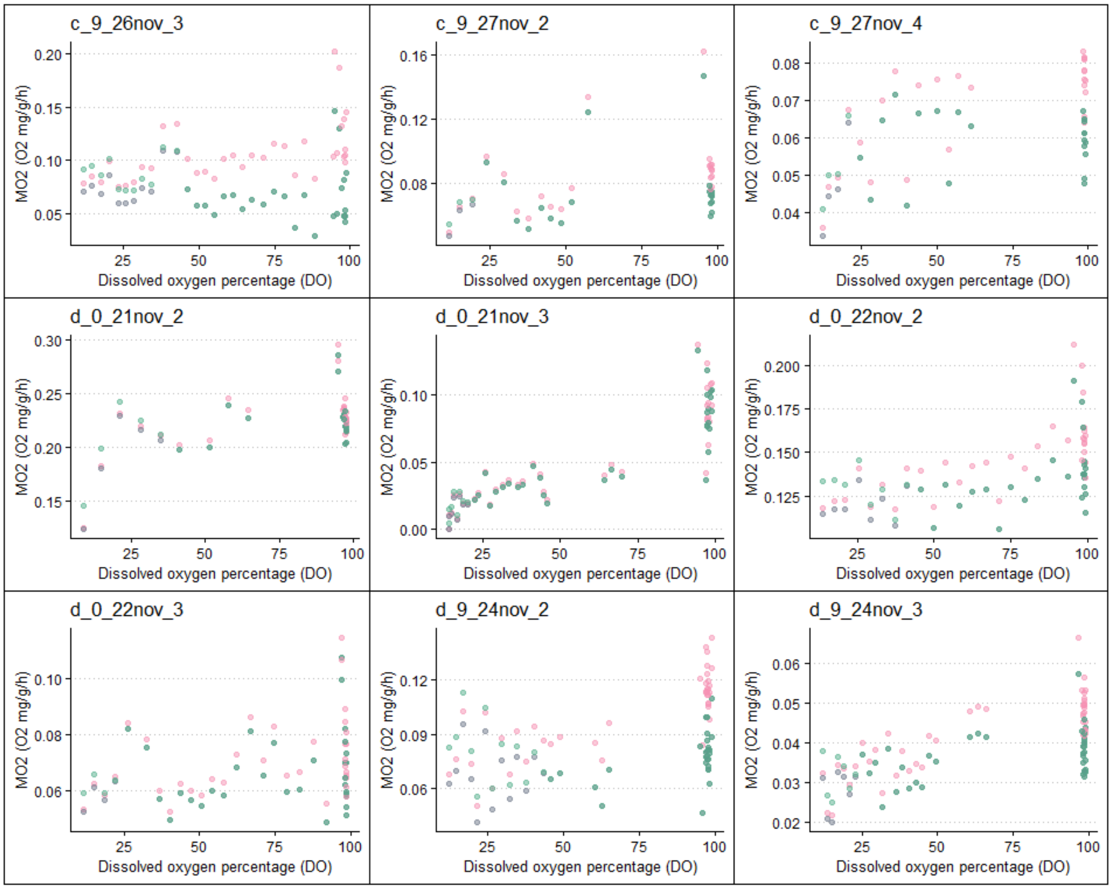

c_9_26nov_3
c_9_27nov_2
c_9_27nov_4
d_0_21nov_2
d_0_21nov_3
d_0_22nov_2
d_0_22nov_3
d_9_24nov_2
d_9_24nov_3
MO2 (O2 mg/g/h)
Dissolved oxygen percentage (DO)

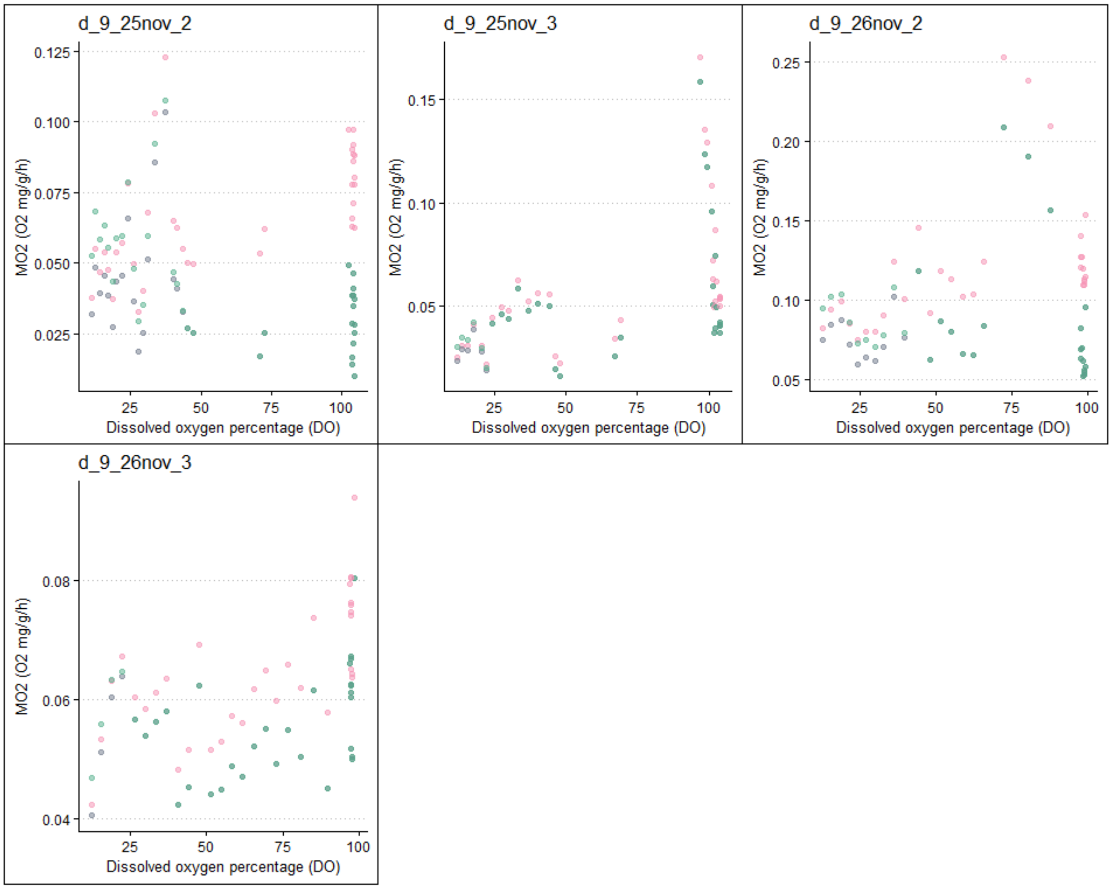

Figure S1: Individual fish-level plots showing the relationship between dissolved oxygen (DO) percentage and metabolic oxygen consumption rate (MO_2, in mg O_2/g/h) for each 20-min interval. Each panel displays data for a single fish (fish ID in the top left of the plot). Points are colour-coded by the type of MO_2 calculation: raw MO_2 (pink, #f692B4), background-corrected MO_2 (grey, #6E7889), and fully corrected MO_2 (accounting for background respiration and oxygen diffusion dynamics) (green, #53af8b). Each point represents a measurement at a specific DO level, and the slight transparency (alpha = 0.5) highlights overlapping observations.

Exploratory Data Analysis (EDA)

O_2 vs MO_2

Figure S2

This interactive was used to identify any outliers, or potential errors. This has not been generated in the PDF `Supplementary file 1`, as it is not compatible.

Figure S2: interactive plot of metabolic rate measurements (MO_2; mg O_2 $g^{-1}h^{-1}$) by dissolved oxygen percentage (DO) for all fish, including all estimates during the SMR phase

(i.e. intermittent phase). Individual linear regression were fitted for visual reference, and do not represent the best fitting regression.

Figure S3

Looking at the difference responses in the two salinity groups.

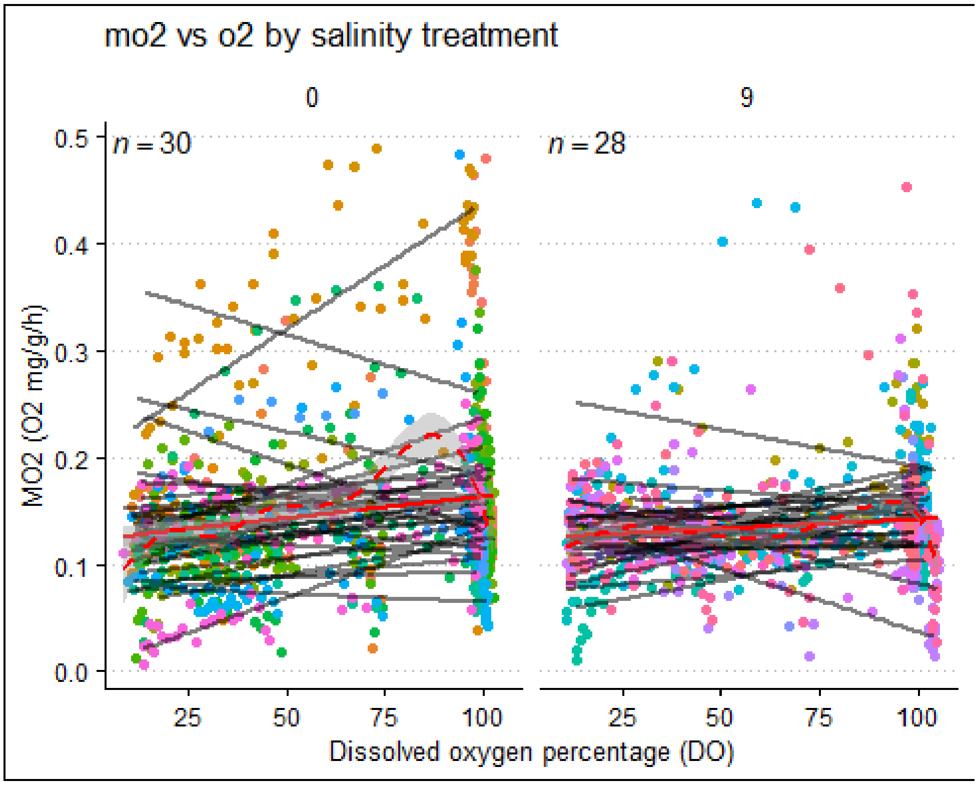

Figure S3: Metabolic rate measurements (MO_2; mg O_2 $g^{-1}h^{-1}$) by dissolved oxygen percentage (DO) for fish from the two salinity treatments, including all estimates during the SMR phase (i.e. intermittent phase). Individual linear regression were fitted for visual reference, and do not represent the best fitting regression. The solid red and dashed red line represents the global trend in each of the treatments, both a linear (solid red) and non-linear (dashed red; Generalized Additive Model fitted with `geom_smooth`).

Figure S4

A plot to look at the different chamber types

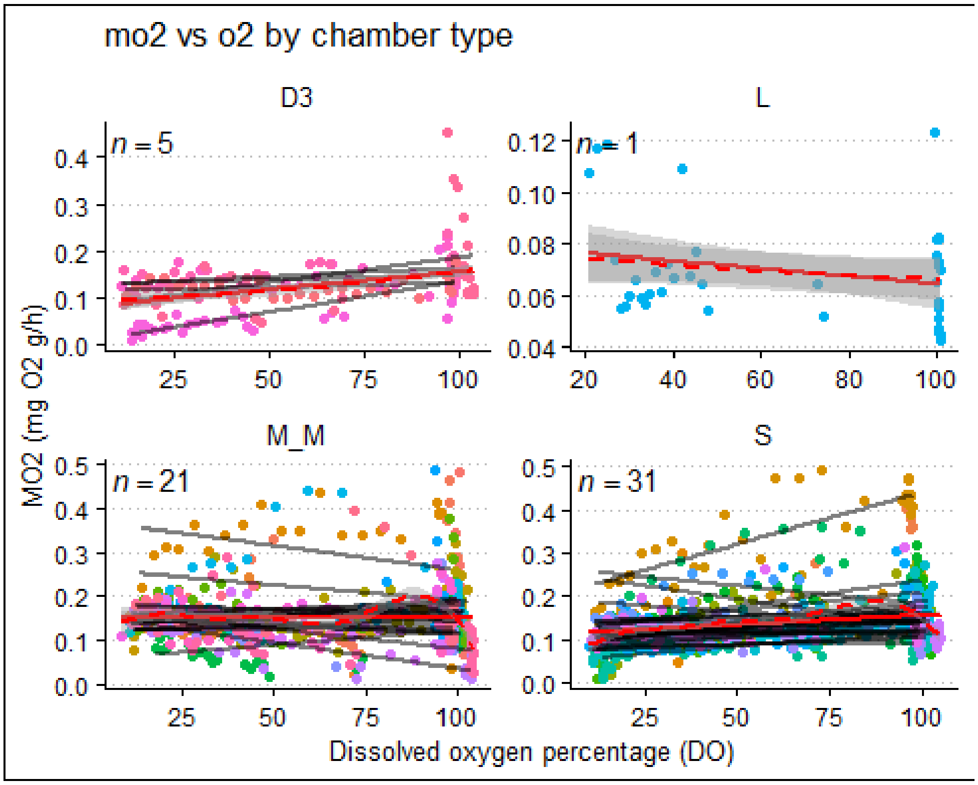

Figure S4: Metabolic rate measurements (MO_2; mg O_2 $g^{-1}h^{-1}$) by dissolved oxygen percentage (DO) for fish tested in the 4 different chamber types, including all estimates during the SMR phase (i.e. intermittent phase). Individual linear regression were fitted for visual reference, and do not represent the best fitting regression. The solid red and dashed red line represents the global trend in each of the treatments, both a linear (solid red) and non-linear (dashed red; Generalized Additive Model fitted with `geom_smooth`).

Routine MO_2

Making an SMR phase only data frame

Routine MO2 by salinity. Plot of all MO2 measures during SMR phase by salinity treatment. The small points are the raw observed values, the shaded area behind the points is the a kernel density of the observed data, the box plot shows the median and interquartile range (IQR), and the large point shows the mean.

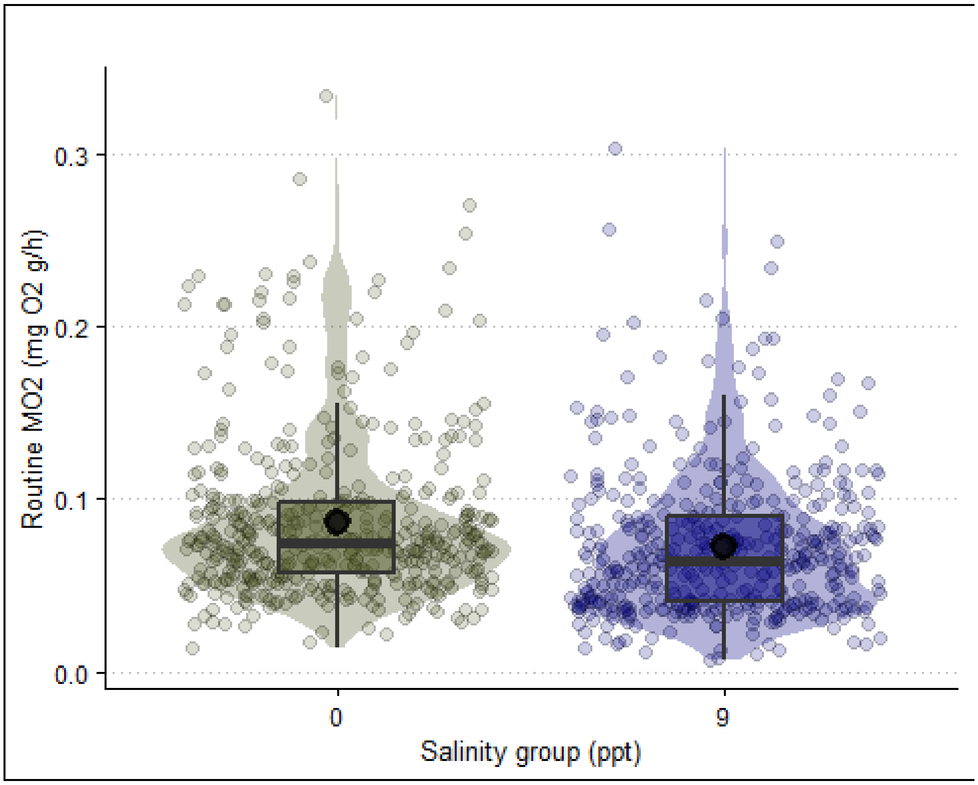

SMR

Here's the same plot but for only the SMR, as estimated with calcSMR function by Chabot, Steffensen and Farrell (2016)[1]. Specifically, we use mean of the lowest normal distribution (MLND) where CVmlnd < 5.4 and the mean of the lower 20% quantile (q0.2) were CVmlnd > 5.4. If CVmlnd is not calculated we have used q0.2.

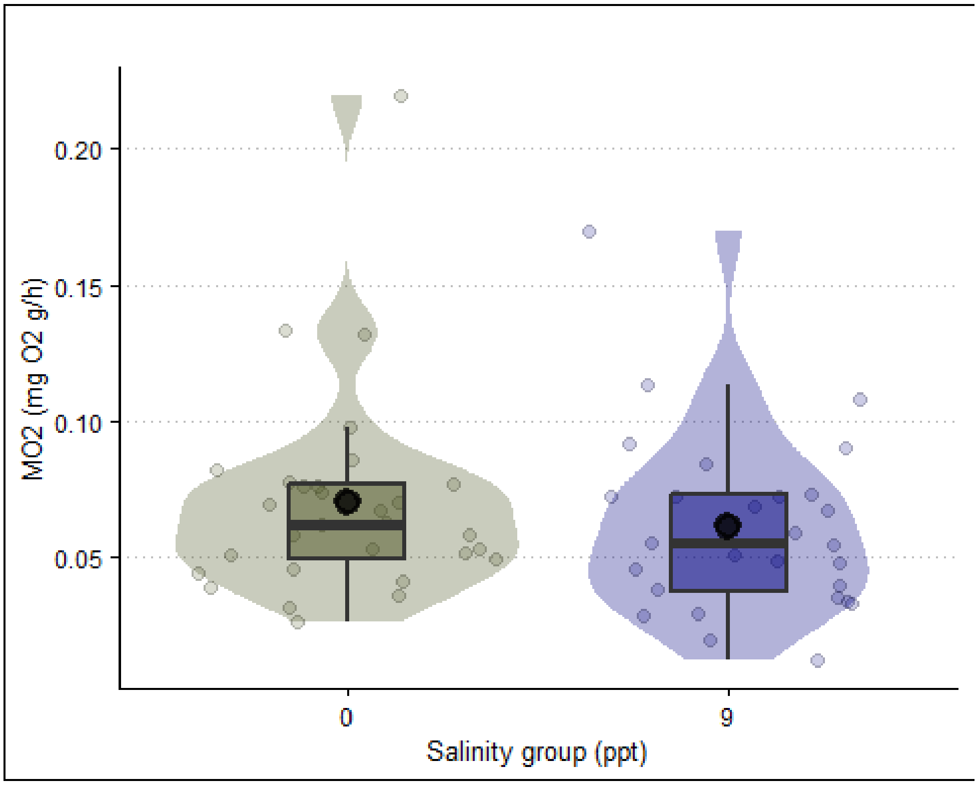

Individual O_2, MO_2, and SMR

Here we will plot the individual relationship between O_2, MO_2, and SMR for all fish

Create output directory if needed

Loop through each fish ID to create a plot, save these to a single PDF file, this is called `combined_slopes.pdf`. It can be found in this path `output-fig/slopes/combined_slopes.pdf` These plots show Metabolic rate measurements (MO_2; mg O_2 $g^{-1}h^{-1}$ by O2) at each of the four experimental phase (the over night SMR intermittent-flow respirometry phase, closed phases at 50%, 75% or 100% O2). The estimated SMR value is represented as a dashed red line.

```
## png
##   2
```

🧮 Analysis

Routine MO2

Scaling predictors

Here we scale our predictors for the model. We have first applied an log transformation to mass, so its directly interpretable as the mass-scaling exponent, just like in metabolic theory.

Summary of predictors used in the model below

temp_range	mass_range	cycle_range	number_fish
13.84–14.38	0.21–1.6	5–27	58

Model structure

Here we will use a Bayesian Generalised Linear Mixed Model (GLMM) with a Gamma distribution and a log link, where the shape parameter (α) is also modelled as a function of predictors. This models MO2 by salinity during the SMR phase to see if the fish held at different salinities have different RMRs. We have also added a few scaled predictors, that may help describe variation in the data, such as mass (g; 0.21–1.6) temperature (°C; 13.84–14.38), cycle order (5–27), and light/dark cycle (light or dark; light between 07:00:00 and 19:00:00), we also include a random effect for fish id to account for multiple MO2 measures on each fish (1–58). We allowed the the shape parameter (α) to vary as a function of some of the predictors (e.g. salinity_group, order_z) to improve fit.

Prior selection

These are the default priors. We will use these.

prior	class	coef	group	resp	dpar	nlpar	lb	ub	source
	b								default
	b	light_dark_c							default
	b	log_mass_c							default
	b	order_c							default
	b	salinity_group9							default
	b	temp_c							default
student_t(3, -2.7, 2.5)	Intercept								default
student_t(3, 0, 2.5)	sd						0		default
	sd		id						default
	sd	Intercept	id						default
	b				shape				default
	b	order_c			shape				default
	b	salinity_group9			shape				default
student_t(3, 0, 2.5)	Intercept				shape				default

Run model

Here we run the model, I have hashed this out because I have saved the model for quick reloading.

⏭️ **Skip this step** if you have downloaded the saved model `mo2_mod_gamma.rds` from GitHub (or pulled the entire GitHub project)

Here we reload the model

Model diagnostics

Checking model convergence

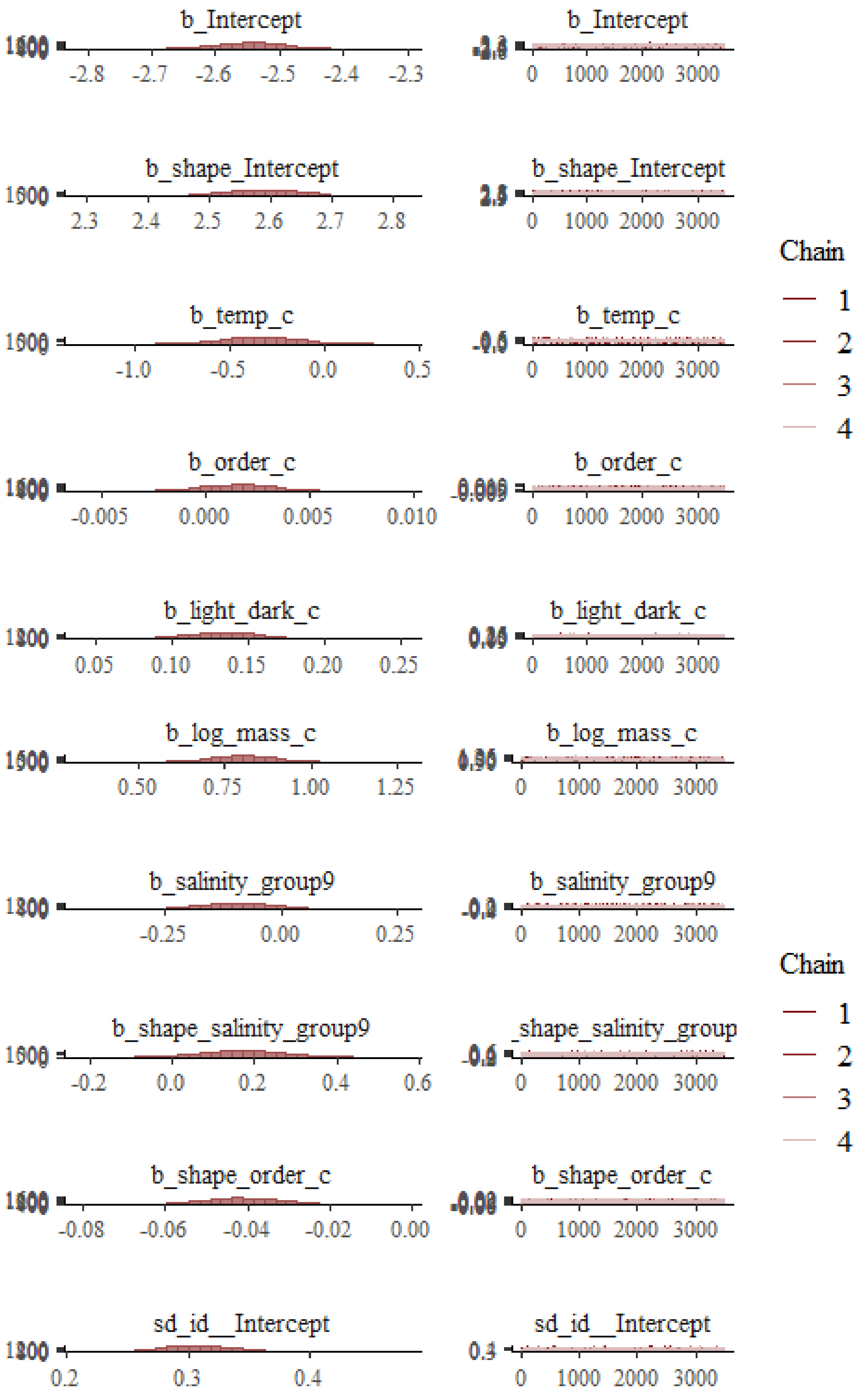

Checking rhat are equal to one

```
##  Family: gamma
##   Links: mu = log; shape = log
```

```
## Formula: MO2 ~ temp_c + order_c + light_dark_c + log_mass_c + 
salinity_group + (1 | id) 
##          shape ~ salinity_group + order_c
##    Data: slope_tidy_smr (Number of observations: 893) 
##   Draws: 4 chains, each with iter = 8000; warmup = 1000; thin = 2;
##          total post-warmup draws = 14000
## 
## Multilevel Hyperparameters:
## ~id (Number of levels: 58) 
##               Estimate Est.Error l-95% CI u-95% CI Rhat Bulk_ESS Tail_ESS
## sd(Intercept)     0.31      0.03     0.26     0.38 1.00     4013     6644
## 
## Regression Coefficients:
##                       Estimate Est.Error l-95% CI u-95% CI Rhat Bulk_ESS
## Intercept                -2.54      0.06    -2.66    -2.43 1.00     2241
## shape_Intercept           2.58      0.07     2.45     2.72 1.00    12143
## temp_c                   -0.33      0.21    -0.75     0.09 1.00     6431
## order_c                   0.00      0.00    -0.00     0.01 1.00    11445
## light_dark_c              0.13      0.02     0.09     0.18 1.00    12311
## log_mass_c                0.81      0.10     0.61     1.01 1.00     2943
## salinity_group9          -0.10      0.08    -0.26     0.07 1.00     2304
## shape_salinity_group9     0.18      0.10    -0.03     0.37 1.00    12404
## shape_order_c            -0.04      0.01    -0.06    -0.02 1.00    11624
##                       Tail_ESS
## Intercept                 4183
## shape_Intercept          12331
## temp_c                    9409
## order_c                  12155
## light_dark_c             12266
## log_mass_c                5032
## salinity_group9           4394
## shape_salinity_group9    12389
## shape_order_c            12394
## 
## Draws were sampled using sampling(NUTS). For each parameter, Bulk_ESS
## and Tail_ESS are effective sample size measures, and Rhat is the potential
## scale reduction factor on split chains (at convergence, Rhat = 1).
```

Using leave one out (loo) measure of fit, the model appears to preform well, all, but one Pareto k estimates are good ($k < 0.7$)

```
## 
## Computed from 14000 by 893 log-likelihood matrix.
## 
##          Estimate   SE
## elpd_loo   2256.8 38.9
## p_loo        71.6  7.1
## looic     -4513.5 77.8
## ------
## MCSE of elpd_loo is NA.
```

```
## MCSE and ESS estimates assume MCMC draws (r_eff in [0.7, 1.0]).
##
## Pareto k diagnostic values:
##                          Count Pct.    Min. ESS
## (-Inf, 0.7]   (good)     892   99.9%   765
##    (0.7, 1]   (bad)        1    0.1%   <NA>
##    (1, Inf)   (very bad)   0    0.0%   <NA>
## See help('pareto-k-diagnostic') for details.
```

Model predictions generally align with the observed data

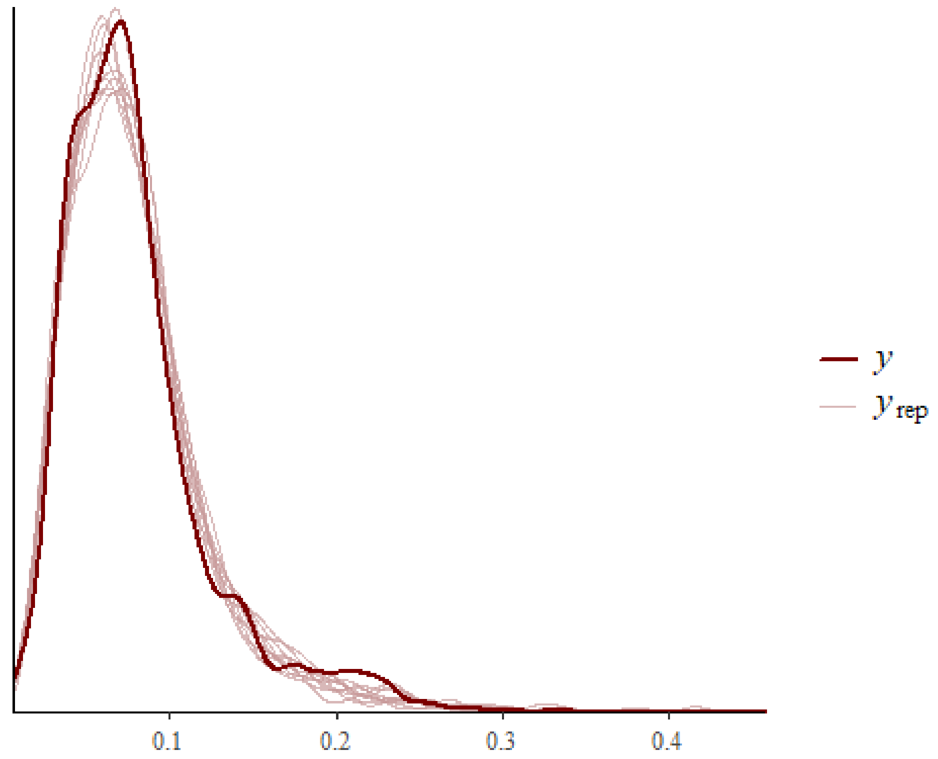

Results

We did not see a meaningful difference between the routine metabolic rate for fish from the two salinity treatments.

Table S1

Table S1: Fixed effect Estimates (β) and 95% Credible Intervals (95% CI)

Predictor	β	95% CI
Intercept	-2.545	[-2.659, -2.428]
shape_Intercept	2.585	[2.45, 2.715]
temp_c	-0.335	[-0.755, 0.087]
order_c	0.002	[-0.002, 0.005]
light_dark_c	0.134	[0.086, 0.182]
log_mass_c	0.805	[0.609, 1.006]
salinity_group9	-0.096	[-0.259, 0.066]
shape_salinity_group9	0.177	[-0.025, 0.372]
shape_order_c	-0.041	[-0.059, -0.023]

Looking at the marginal mean MO2 for each salinity treatment

salinity_group	emmean	lower.HPD	upper.HPD
0	0.079	0.070	0.088
9	0.071	0.064	0.080

These are the estimated mean constrast, we have exponentiated the log estimate to get the fold-change. This means the model predicts that sailianty group 0 has ~12% higher metabolic rate than group 9 (1.12)—but this difference is not credible, because the interval for that difference includes no difference (i.e. a ratio of 1.0).

contrast	estimate	lower.HPD	upper.HPD
salinity_group0 - salinity_group9	1.1	0.94	1.29

EEM contrasts for the two light phases, where -0.5 is dark, and 0.5 is light

contrast	estimate	lower.HPD	upper.HPD
light_dark_c0.5 - (light_dark_c-0.5)	1.14	1.09	1.2

Here we we calculate the model-estimated marginal means for MO2 for a unit increase in mass and transform this onto a fold-change scale. Where log(mass) has be centered, a vaule of 0 represents the mean mass of fish, and 1 corresponds to a fish with log(mass) = 1 unit above the mean (i.e. 2.7 times large).

$\log(mass2) = \log(mass1)+1$

If you exponentiate both sides:

$mass2 = mass1 \times \exp(1)$

$mass2 \approx mass1 \times 2.718$

contrast	estimate	lower.HPD	upper.HPD
log_mass_c1 - log_mass_c0	2.23	1.86	2.76

Pulling the emmeans draws for our plot

Figure 1a

NOTE: This plot is in the main text of the manuscript, which is way it is called Figure 1a (not Figure Si)

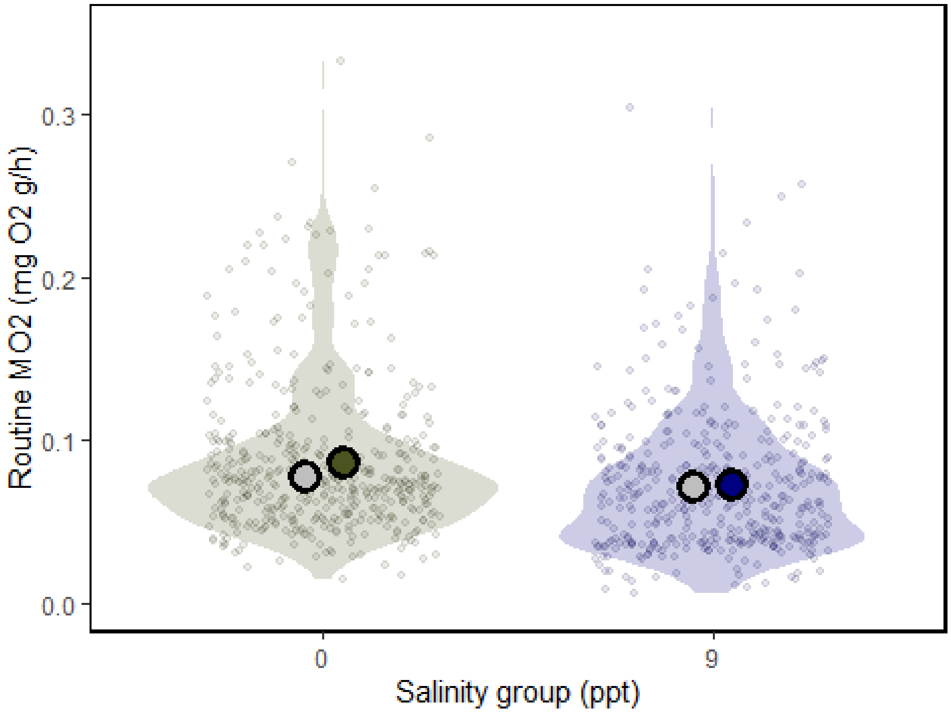

Figure 1a: Routine metabolic rate (i.e. MO2 (mg^{-1} O2 h^{-1}) measured during SMR meassurments) plotted by salinity treatment. The small transparent points are the observed values, the shaded area behind the points is the a kernel density of the observed data, the large coloured point (to the right) is the observed mean, the large grey point with error bars (to the left) is the model estimated marginal mean (eemean) 95% Credible Intervals (95% CI).

There was moderate but meaningful variation in RMR between individuals, even after accounting for mass, temperature, salinity, and light phase. The estimated standard deviation of individual RMR was 0.31 [0.26 to 0.38], indicating that individual identity explained a meaningful proportion of the observed variation.

Estimate	Est.Error	l-95% CI	u-95% CI	Rhat	Bulk_ESS	Tail_ESS
0.3109591	0.0317821	0.2555882	0.3785216	1.000639	4013.05	6644.265

SMR

Formating and scaling data

Here we are filtering the data frame to have only measure per fish for the SMR estimate

Summary of predictors used in the model below

temp_range	mass_range	cycle_range	number_fish
13.87–14.26	0.21–1.6	10–23	58

Model structure

Here we will use a Bayesian Generalised Linear Mixed Model (GLMM) with a Gamma distribution and a log link `Gamma(link = "log")`, where the shape parameter (α) is also modelled as a function of the salinity group, `shape ~ salinity_group`. This models MO2 by salinity during the SMR phase to see if the fish held at different salinities have different SMRs. We have also added a few scaled predictors, that may help describe variation in the data, such as fish mass (g; 0.21–1.6) mean temperature (°C; 13.841–14.277), and the number of cycles over which SMR was estimated (1–23). We allowed the the shape parameter (α) to vary as a function of salinity_group to improve fit.

Prior selection

These are the default priors for the model. We will use these.

prior	class	coef	group	resp	dpar	nlpar	lb	ub	source
	b								default
	b	cycles_c							default
	b	log_mass_c							default
	b	salinity_group9							default
	b	temp_mean_c							default
student_t(3, -2.8, 2.5)	Intercept								default
	b				shape				default
	b	salinity_group9			shape				default
student_t(3, 0, 2.5)	Intercept				shape				default

Run model

Here we run the model, I have hashed this out because I have saved the model for quick reloading.

⏭️ **Skip this step** if you have downloaded the saved model 'smr_mod_gamma.rds'

Here we reload the model

Model diagnostics

Checking model convergence

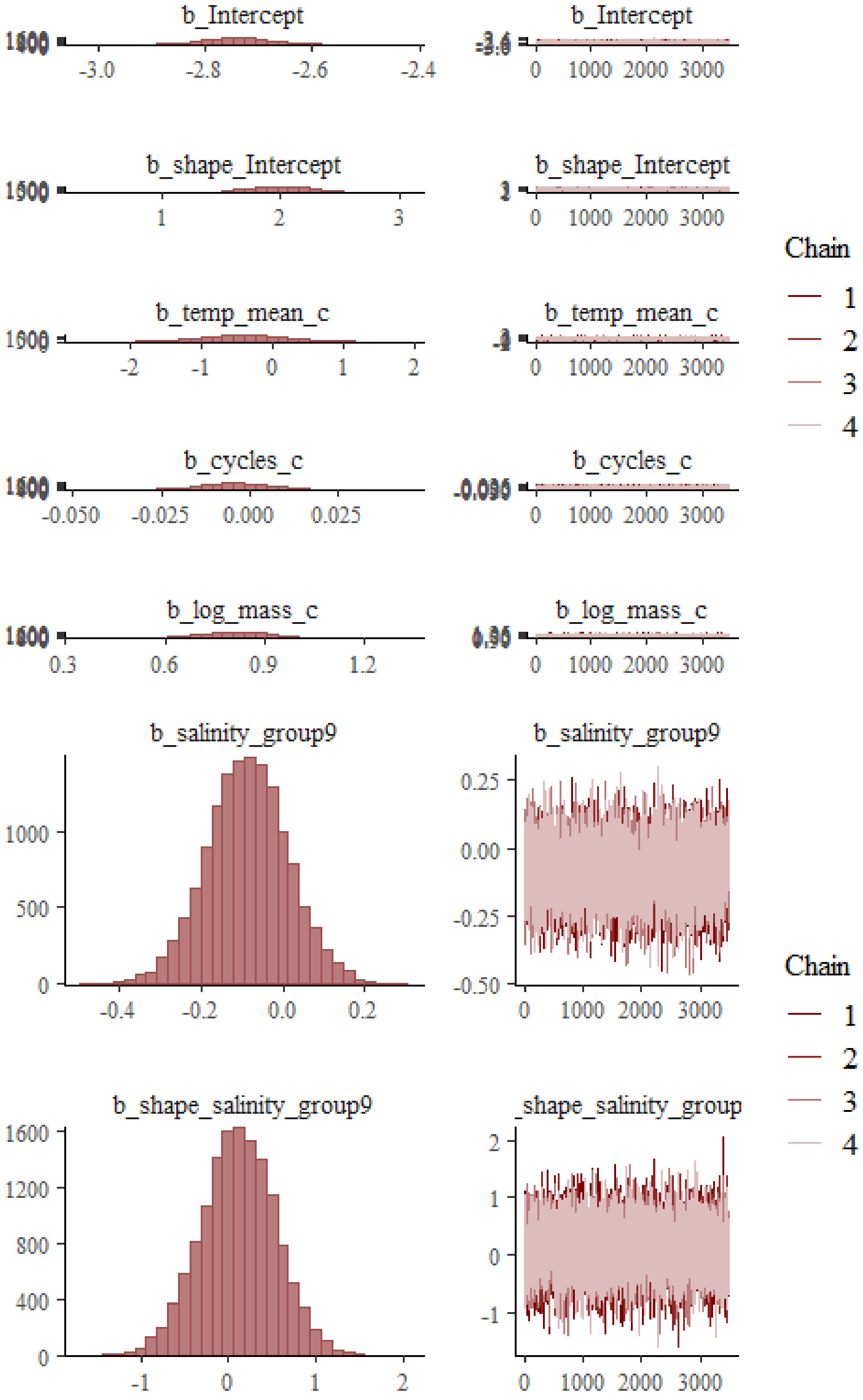

Using leave one out (loo) measure of fit, the model appears to preform well, two Pareto k estimates falls outside the good range (0.7, 1]

```
##
## Computed from 14000 by 58 log-likelihood matrix.
##
##          Estimate   SE
## elpd_loo    134.6 11.0
## p_loo        11.0  4.8
## looic      -269.2 22.0
## ------
## MCSE of elpd_loo is NA.
## MCSE and ESS estimates assume MCMC draws (r_eff in [0.8, 0.9]).
##
## Pareto k diagnostic values:
##                          Count Pct.    Min. ESS
## (-Inf, 0.7]   (good)     56    96.6%   4028
##    (0.7, 1]   (bad)       1     1.7%   <NA>
##    (1, Inf)   (very bad)  1     1.7%   <NA>
## See help('pareto-k-diagnostic') for details.
```

Model predictions generally align with the observed data, but there is a lot of uncertainty around this estimate.

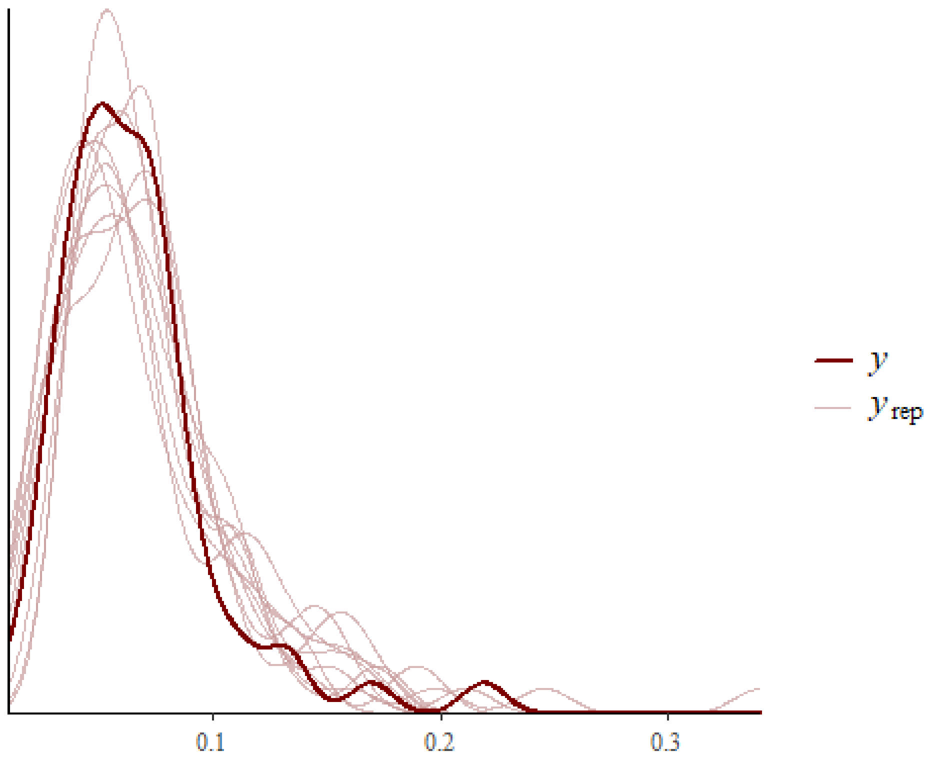

Results

We did not see a meaningful difference between the routine metabolic rate for fish from the two salinity treatments.

Table S2

Table S2: Fixed effect Estimates (β) and 95% Credible Intervals (95% CI) from a Bayesian Generalised Linear Mixed Model (GLMM) with a Gamma distribution and a log link

Predictor	β	95% CI
Intercept	-2.740	[-2.879, -2.603]
shape_Intercept	2.010	[1.428, 2.532]
temp_mean_c	-0.364	[-1.51, 0.802]
cycles_c	-0.005	[-0.026, 0.018]
log_mass_c	0.816	[0.597, 1.034]
salinity_group9	-0.086	[-0.283, 0.111]
shape_salinity_group9	0.117	[-0.733, 0.945]

Looking at the marginal mean difference between salinity groups

salinity_group	emmean	lower.HPD	upper.HPD
0	0.065	0.056	0.074
9	0.059	0.052	0.067
contrast	estimate	lower.HPD	upper.HPD
salinity_group0 - salinity_group9	1.09	0.89	1.32

Here we we calculate the model-estimated marginal means for SMR for a unit increase in mass and transform this onto a fold-change scale. Where log(mass) has be centered, a vaule of 0 represents the mean mass of fish, and 1 corresponds to a fish with log(mass) = 1 unit above the mean (i.e. 2.7 times large).

contrast	estimate	lower.HPD	upper.HPD
log_mass_c1 - log_mass_c0	2.26	1.84	2.84

Pulling the emmeans draws for our plot

Figure 1b

NOTE: This plot is in the main text of the manuscript which is why it is Figure 1b (not Figure Si)

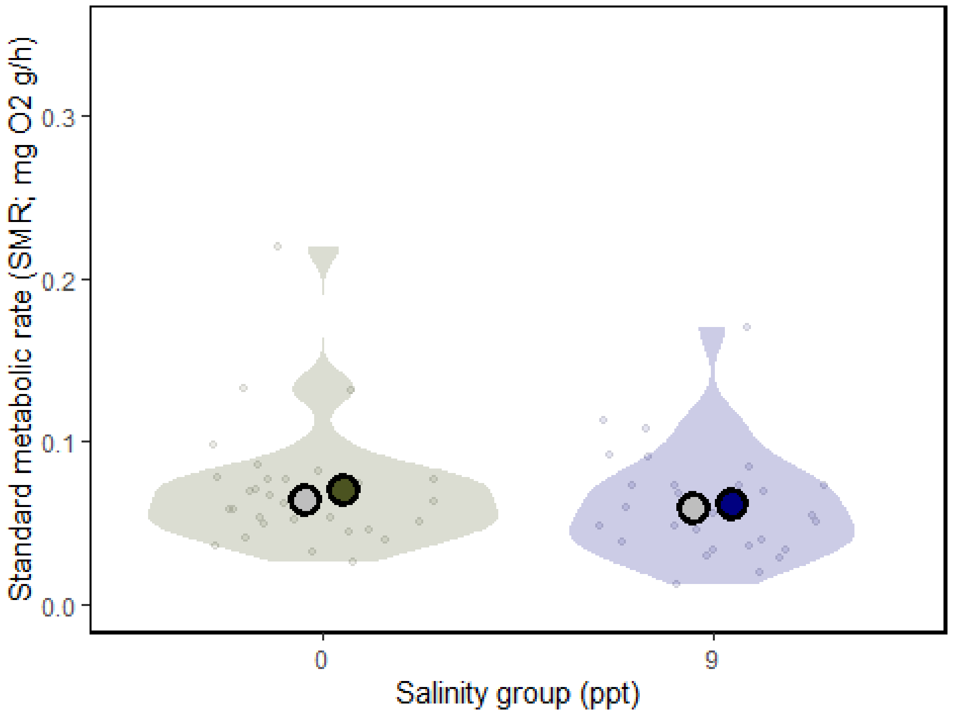

Figure 1b: The standard metabolic rate estimate (mg^{-1} O2 h^{-1}) plotted by salinity treatment. The small transparent points are the observed values, the shaded area behind the points is the a kernel density of the observed data, the large coloured point (to the right) is the observed mean, the large grey point with error bars (to the left) is the model estimated marginal mean (eemean) 95% Credible Intervals (95% CI).

Incremental regression analyses

Here we are following the methods Urbina et al. (2012)[1] with an incremental regression analyses, in order to determine the best fit for the MO2 vs O2 data

This analysis approach evaluates the relative 'fit' of each polynomial order equation starting at zero and increasing to the third order, permitting a mathematical assessment of whether fish were oxyconforming or oxyregulatoring. If the data is best fitted/predicted by a single linear relationship (1st-order polynomial) with a positive slope, this would suggests the fish were oxyconforming. Alternately, if the relationship is best modelled by a flat regression (0th-order polynomial), or a higher order polynomial (2nd or 3rd-order polynomial) the fish is likely oxyregulatoring.

Building Bayesian regressions

Here we are using a Bayesian approach to model fitting with brm. These models take a long time to run, so I have saved them and re-loaded them to save time. I have also saved the summary data produced from the models, to save time, you can simply skip the hashed code and input the resulting summary data.

We will run our custom function, `bayes_incremental_regression_by_id`.

Let's make define the output directory

⏭ **Skip this step** if you have already run this once, or have downloaded the saved models or saved data files from GitHub (that's why its hashed out). ⌛*This code takes a while to run* ⌛

During this part of the script I received an error, 'Caused by error in `socketConnection()`:'. I think the system may be hitting a limit on the number of simultaneous socket connections. If you this part of the code and also get this issue, try reducing the number of parallel workers: plan(multisession, workers = 2), or run again after the error.

Load all models and store in a list, will use a lot of memory. You can also skip this step and load the resulting data frames below. I am using the custom function `load_rds`, so we can compare them and generate predictions.

⏭ **Skip this step** if you have downloaded the saved data pulled from these models have also hased this out, bayes_reg_mods_fit.csv' and 'bayes_reg_mods_predictions.csv'. These are in the mod-data directory.

Model fits

⏭ **Skip this step** if you have downloaded 💿 **bayes_reg_mods_fit.csv**.

It gets model fit parameters loo and r2 using the custom function, `incremental_regression_bayes_fits`. ⌛*This code takes a while to run* ⌛

Reading in this model fit data frame, in the case you did not load in all the models.

Selecting the best fitting model

elpd_loo, or the expected log pointwise predictive density for leave-one-out cross-validation, is a metric used in Bayesian model evaluation to assess the predictive accuracy of a model. The elpd_loo is an approximation of how well the model is expected to predict new data, based on leave-one-out cross-validation. Higher elpd_loo values indicate better predictive performance.

Model predictions

⏭ **Skip this step** if you have downloaded 💿 **bayes_reg_mods_predictions.csv**.

It pulls our model predictions using a custom function `bayes_mod_predictions`.

Reading in the predicted data

We are going to combined this with our best fitting model df, so we know how they ranks for LOO.

Results

Model selection summary

In most cases, the relationship between MO2 and DO was best modelled with a 2nd-order polynomial (*n* = 22, 38% of fish), followed by a 3rd-order polynomial (*n* = 15, 26%), 1st-order polynomial (*n* = 11, 19%), and finally 0th-order polynomial (*n* = 10, 17%).

For the two most common models, 2nd- and 3rd-order polynomials, this could suggest the presence of a critical oxygen threshold (Pcrit) where the relationship between MO2 and O2 changes. To confirm the presence of a Pcrit, we need to validated the shape of the polynomials (see Pcrit model below). In any case, these type of models are indicative of **oxyregulator**.

The next most common model was is 1st-order polynomial. In the case of the 1st-order polynomials, it suggest the presences of linear relationship between o2 and MO2, which is indicative of **oxyconformer**. However, to be true evidence of a oxyconformer this relationship should be positive (i.e. as O2 falls MO2 also falls). 13 of the 18 individuals best modelled with a linear function had positive estimates with credible intervals that did not overlap with zero (Table S3).

Lastly, 0th-order was the least common (*n* = 3, 5%), it suggests that MO2 does not show a statistically significant dependence on the O2. In other words, the metabolic rate does not adjust based on oxygen availability, and there is no clear critical oxygen threshold (Pcrit) where the relationship changes. This is indicative of a **oxyregulator**.

best_model_name	n	percent
0th-order polynomial	10	17.24
1st-order polynomial	11	18.97
2nd-order polynomial	22	37.93
3rd-order polynomial	15	25.86

Summary of fish best model with a linear function.

Mass effect

Checking if this pattern is driven by mass, as request by the reviewer.

Best polynomial fit	Mean mass (g)	SD mass (g)	n
0th-order polynomial	0.56	0.32	10
1st-order polynomial	0.50	0.23	11
2nd-order polynomial	0.61	0.32	22
3rd-order polynomial	0.47	0.13	15

Modling

Prior selection

These are the default priors for the model. We will use these.

prior	class	coef	group	resp	dpar	nlpar	lb	ub	source
	b								default
	b	model_typelm_1							default
	b	model_typelm_2							default
	b	model_typelm_3							default
student_t(3, 0.5, 2.5)	Intercept								default
student_t(3, 0, 2.5)	sigma						0		default

Run model

Here we run the model, I have hashed this out because I have saved the model for quick reloading.

⏭️ **Skip this step** if you have downloaded the saved model ‘mass_mod.rds’

Here we reload the model

Model diagnostics

Checking model convergence

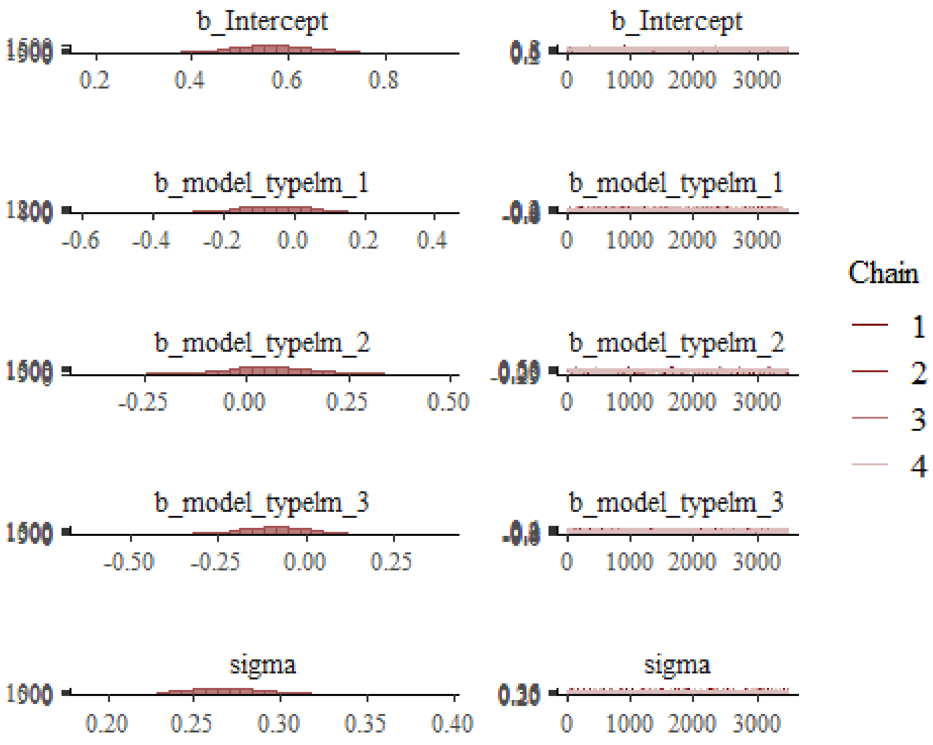

Using leave one out (loo) measure of fit, the model appears to preform well, two Pareto k estimates falls outside the good range (0.7, 1]

```
##
## Computed from 14000 by 58 log-likelihood matrix.
##
##          Estimate   SE
## elpd_loo    -10.2 10.3
## p_loo         6.8  2.8
## looic        20.5 20.6
## ------
## MCSE of elpd_loo is 0.1.
## MCSE and ESS estimates assume MCMC draws (r_eff in [0.8, 1.0]).
##
## All Pareto k estimates are good (k < 0.7).
## See help('pareto-k-diagnostic') for details.
```

Model predictions generally align with the observed data, but there is a lot of uncertainty around this estimate.

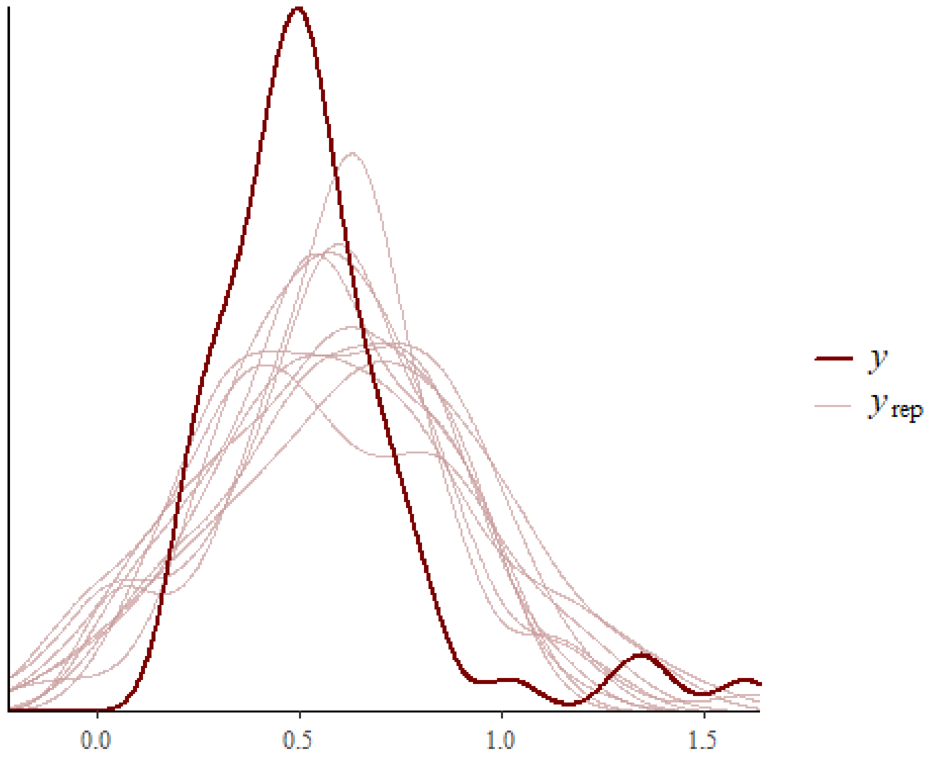

Results

Predictor	β	95% CI
Intercept	0.561	[0.39, 0.729]
model_typelm_1	-0.057	[-0.294, 0.179]
model_typelm_2	0.051	[-0.156, 0.256]
model_typelm_3	-0.090	[-0.314, 0.131]

Looking at the marginal mean difference between salinity groups

model_type	emmean	lower.HPD	upper.HPD
lm_0	0.560	0.389	0.728
lm_1	0.504	0.342	0.668
lm_2	0.611	0.504	0.733
lm_3	0.470	0.330	0.609

Table S3

Table S3: Posterior mean differences in mass (g) (estimate) and 95% highest posterior density intervals (95% CrI) are shown for all pairwise comparisons among model types (0th- to 3rd-order polynomial). Estimates are derived from a Gaussian Bayesian model and represent differences in marginal posterior means on the response scale. A meaningful difference was inferred when the 95% CrI did not overlap zero.

Contrast	Estimate (g)	95% CrI
0th-order polynomial − 1st-order polynomial	0.06	-0.18 to 0.29
0th-order polynomial − 2nd-order polynomial	-0.05	-0.26 to 0.15
0th-order polynomial − 3rd-order polynomial	0.09	-0.12 to 0.32
1st-order polynomial − 2nd-order polynomial	-0.11	-0.3 to 0.1
1st-order polynomial − 3rd-order polynomial	0.03	-0.19 to 0.24
2nd-order polynomial − 3rd-order polynomial	0.14	-0.04 to 0.32

Table S4

Table S4: Ten fish that were best modelled with a linear function, showing r^2, and estimate (). Only two fish had positive estimates with credible intervals that did not overlap with zero, which are highlighted as conforming in the table. Thus in total, 13 of 58 fish showed sufficient evidence to be conforming.

Fish ID	r2 (CI)	Estimate (CI)	Percentage change	Evidence of oxyconforming
d_9_25nov_3	0.197 (0.012 to 0.394)	0.0011 (0.0003 to 0.0019)	0.7437151	Conforming
a_9_22nov_4	0.312 (0.094 to 0.491)	0.0008 (0.0004 to 0.0013)	0.5739880	Conforming
b_9_22nov_1	0.231 (0.055 to 0.396)	0.0005 (0.0002 to 0.0008)	0.4310066	Conforming
d_9_25nov_2	0.330 (0.102 to 0.507)	-0.0009 (-0.0013 to -0.0004)	-0.7599381	Not conforming
a_0_25nov_1	0.137 (0.002 to 0.331)	-0.0004 (-0.0007 to 0.0000)	-0.2788575	Not conforming
c_0_22nov_3	0.072 (0.000 to 0.256)	0.0005 (-0.0005 to 0.0016)	0.3013443	Not conforming
a_0_24nov_3	0.058 (0.000 to 0.202)	0.0005 (-0.0003 to 0.0012)	0.2832654	Not conforming
c_9_27nov_2	0.068 (0.000 to 0.255)	0.0003 (-0.0003 to 0.0008)	0.1697994	Not conforming
a_9_22nov_1	0.084 (0.000 to 0.266)	0.0003 (-0.0002 to 0.0008)	0.2272965	Not conforming
b_0_25nov_2	0.108 (0.001 to 0.290)	0.0003 (0.0000 to 0.0006)	0.2275231	Not conforming
a_0_24nov_1	0.029 (0.000 to 0.134)	0.0002 (-0.0005 to 0.0009)	0.1072286	Not conforming

Ploting all models

Data set with all slopes and which model was best

Saving all regression, and highlighting the model that has the best fit, based on AIC values. This is called `combined_reg_plots.pdf`

Getting a plot for each best fit regression, and overlaying a global model for that model type.

Here we are grouping fish by best fitting model and getting an average trend. I have hashed the code so you don't need to re-run the models

⏭ **Skip this step** if you have downloaded the global models **'.rds'** in the 'bayes-regs-global' folder

⏭ **Skip this step** if you have downloaded the global_models_pred_df.csv

Load data frame

Figure S5

Best fit regressions with global models based on that polynomial order.

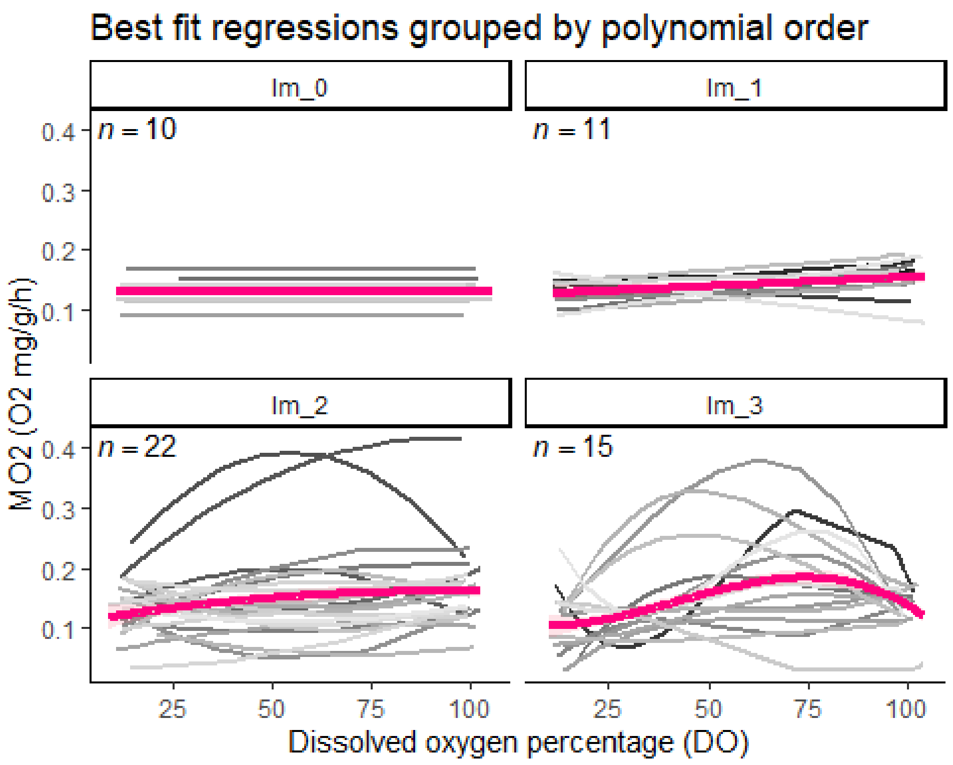

Figure S5: Individual regression curves showing the relationship between mass-specific oxygen consumption rate ($\dot{M}O_2$; mg^{-1} O_2 h^{-1}) and ambient dissolved oxygen (DO; % saturation), grouped by the best-fitting polynomial order (0th to 3rd). Each panel represents fish whose $\dot{M}O_2$–DO relationship was best modelled by that polynomial order, based on leave-one-out cross-validation (LOO-CV). Grey lines represent individual model fits; the bold pink line shows the group-level trend within each polynomial class. Fish were most commonly best fit by a 2nd-order polynomial (n = 22), followed by 3rd-order (*n* = 15), 1st-order (*n* = 11), and 0th-order (**n = 10). Higher-order fits (2nd and 3rd) and 0th-order fits are generally indicative of oxyregulating responses, while 1st-order fits may suggest oxyconforming behaviour when positively sloped.

O_2crit model

We will calculate O_2crit using Chabot method and function `calcO2crit`.

This function uses the fifth percentile of the MO2 values observed at dissolved oxygen levels ≥ 80% air saturation as the criterion to assess low MO2 values. The algorithm then identifies all the MO2 measurements greater than this minimally acceptable MO2 value. Within this sub-set, it identifies the MO22 measurement made at the lowest DO and thereafter considers this DO as candidate for breakpoint (named pivotDO in the script). A regression is then calculated using observations at DO levels < pivotDO, and a first estimate of O2crit is calculated as the intersection of this regression line with the horizontal line representing SMR. The script then goes through validation steps to ensure that the slope of the regression is not so low that the line, projected to normoxic DO levels, passes below any

MO2 values observed in normoxia. It also ensures that the intercept is not greater than zero. Corrective measures are taken if such problems are encountered.

lowestMO2 default is the `quantile(MO2[DO >= 80], p=0.05)`. It is used to segment the data and locate the pivotDO.

Ploting O2crit

Here's we are saving all O2crit models, even those MO2-O2 relationships best modelled by 0th- and 1st-order polynomials.

```
## png
##   2
```

Now printing the fish that were fitted with higher order-polynomials in the htlm document

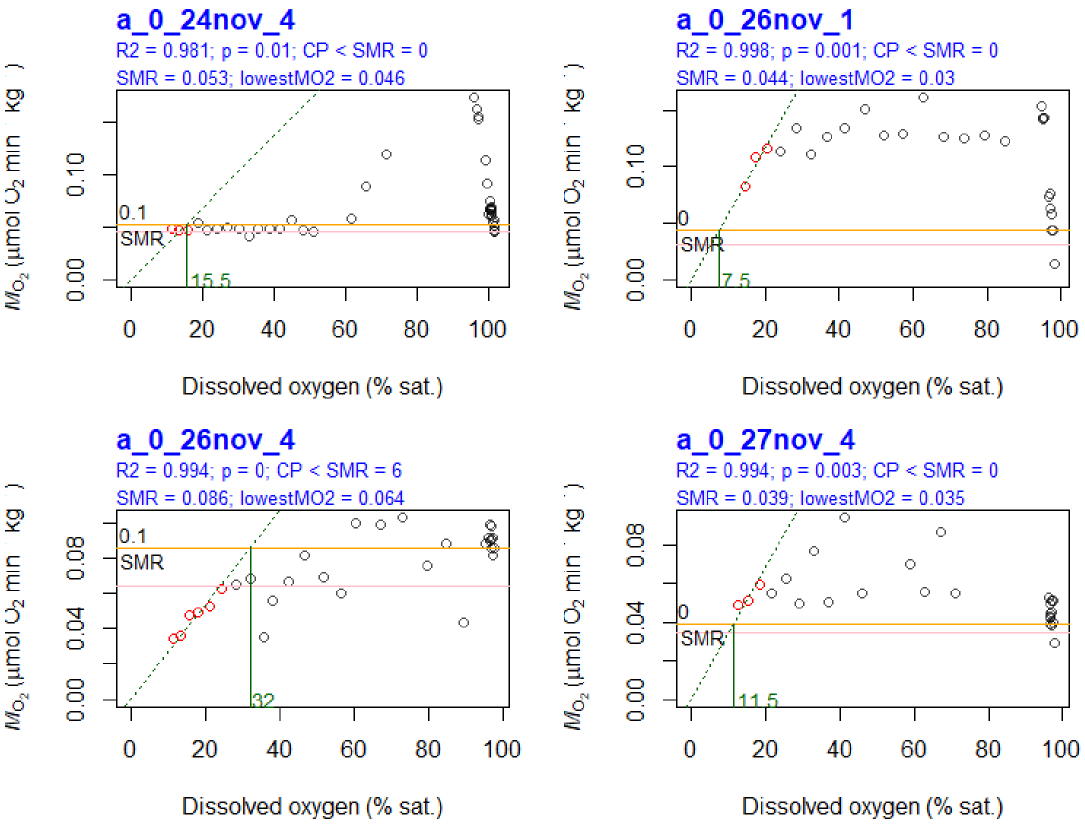

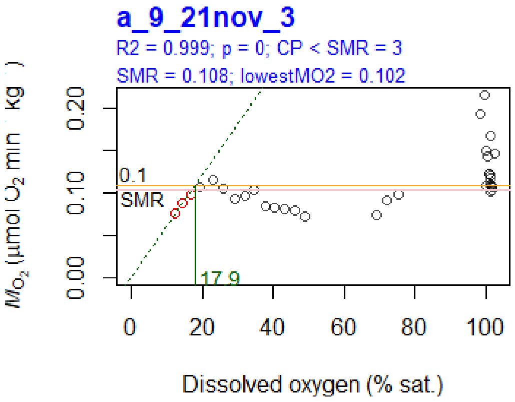
a_9_21nov_3
R2 = 0.999; p = 0; CP < SMR = 3
SMR = 0.108; lowestMO2 = 0.102
0.1
SMR
17.9
Dissolved oxygen (% sat.)

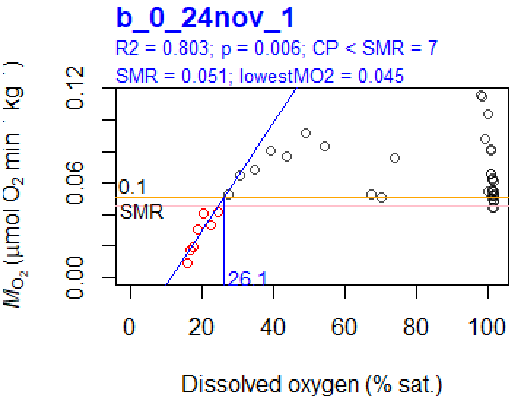
b_0_24nov_1
R2 = 0.803; p = 0.006; CP < SMR = 7
SMR = 0.051; lowestMO2 = 0.045
0.1
SMR
26.1
Dissolved oxygen (% sat.)

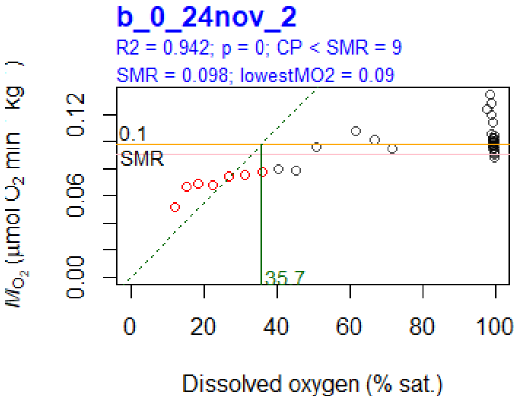
b_0_24nov_2
R2 = 0.942; p = 0; CP < SMR = 9
SMR = 0.098; lowestMO2 = 0.09
0.1
SMR
35.7
Dissolved oxygen (% sat.)

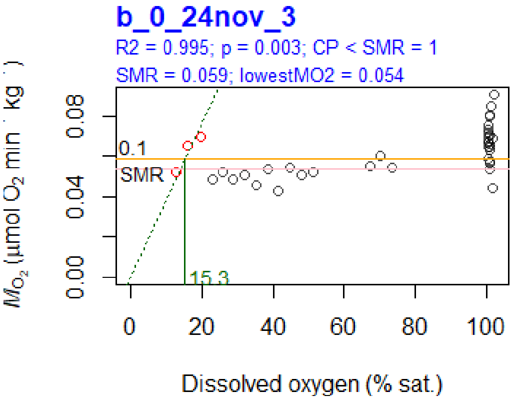
b_0_24nov_3
R2 = 0.995; p = 0.003; CP < SMR = 1
SMR = 0.059; lowestMO2 = 0.054
0.1
SMR
15.3
Dissolved oxygen (% sat.)

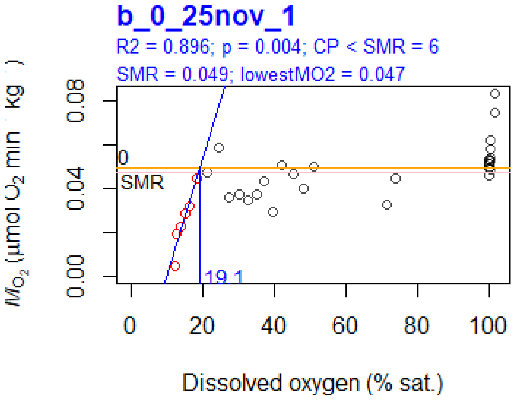
b_0_25nov_1
R2 = 0.896; p = 0.004; CP < SMR = 6
SMR = 0.049; lowestMO2 = 0.047
0
SMR
19.1
Dissolved oxygen (% sat.)

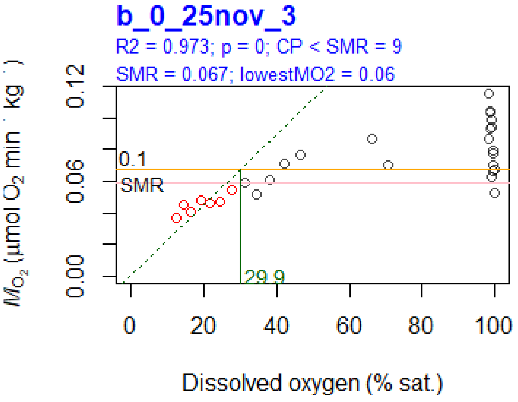
b_0_25nov_3
R2 = 0.973; p = 0; CP < SMR = 9
SMR = 0.067; lowestMO2 = 0.06
0.1
SMR
29.9
Dissolved oxygen (% sat.)

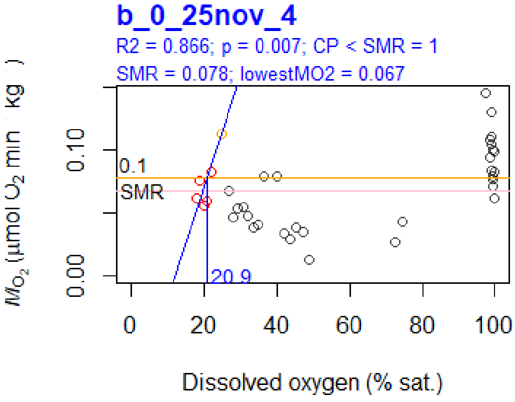
b_0_25nov_4
R2 = 0.866; p = 0.007; CP < SMR = 1
SMR = 0.078; lowestMO2 = 0.067
0.1
SMR
20.9
Dissolved oxygen (% sat.)

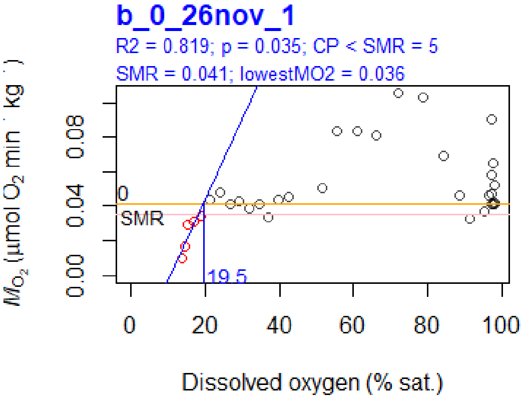
b_0_26nov_1
R2 = 0.819; p = 0.035; CP < SMR = 5
SMR = 0.041; lowestMO2 = 0.036
0
SMR
19.5
Dissolved oxygen (% sat.)

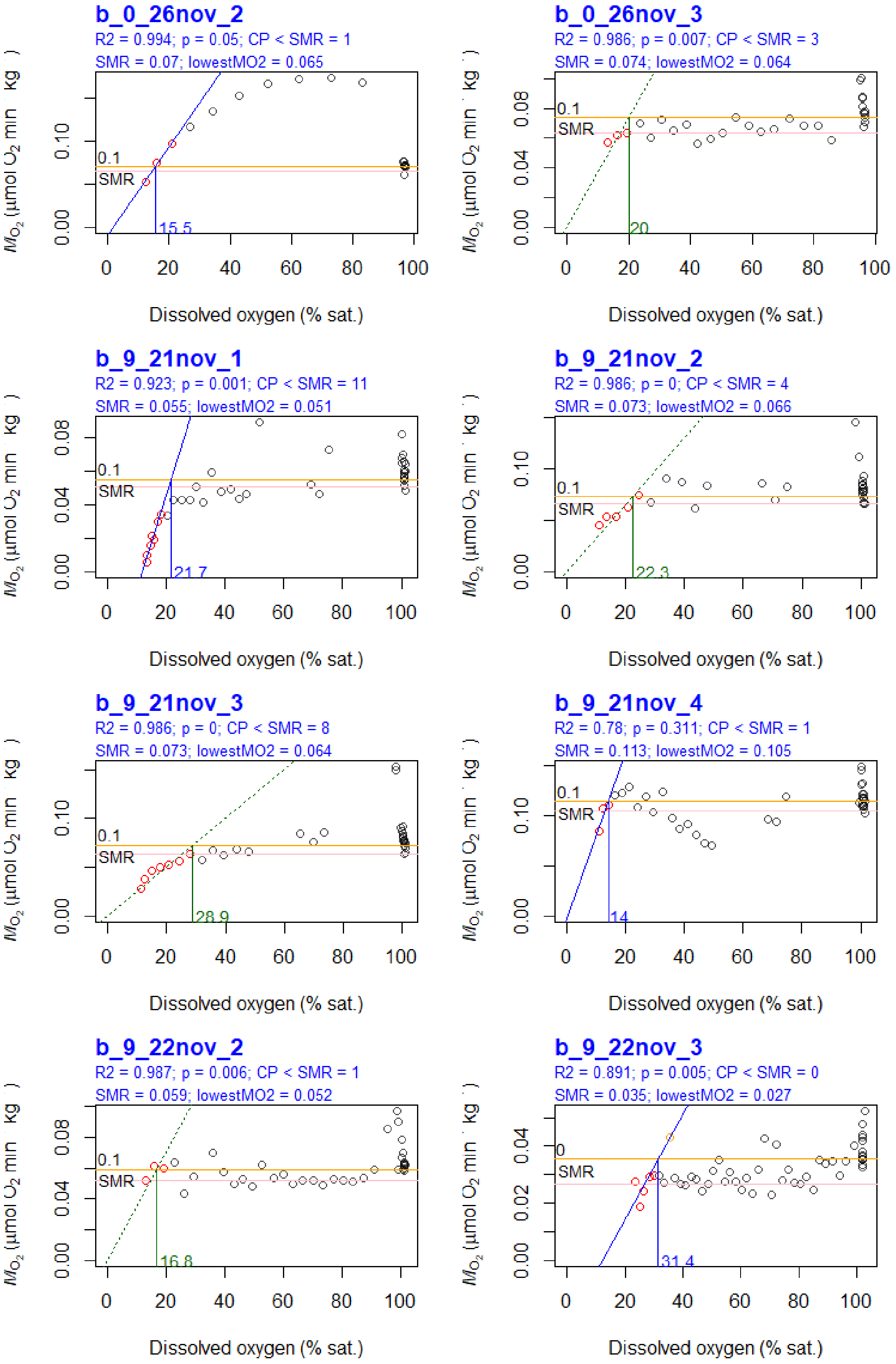
b_0_26nov_2
R2 = 0.994; p = 0.05; CP < SMR = 1
SMR = 0.07; lowestMO2 = 0.065
15.5
b_0_26nov_3
R2 = 0.986; p = 0.007; CP < SMR = 3
SMR = 0.074; lowestMO2 = 0.064
20
b_9_21nov_1
R2 = 0.923; p = 0.001; CP < SMR = 11
SMR = 0.055; lowestMO2 = 0.051
21.7
b_9_21nov_2
R2 = 0.986; p = 0; CP < SMR = 4
SMR = 0.073; lowestMO2 = 0.066
22.3
b_9_21nov_3
R2 = 0.986; p = 0; CP < SMR = 8
SMR = 0.073; lowestMO2 = 0.064
28.9
b_9_21nov_4
R2 = 0.78; p = 0.311; CP < SMR = 1
SMR = 0.113; lowestMO2 = 0.105
14
b_9_22nov_2
R2 = 0.987; p = 0.006; CP < SMR = 1
SMR = 0.059; lowestMO2 = 0.052
16.8
b_9_22nov_3
R2 = 0.891; p = 0.005; CP < SMR = 0
SMR = 0.035; lowestMO2 = 0.027
31.4
0.1
SMR
Dissolved oxygen (% sat.)

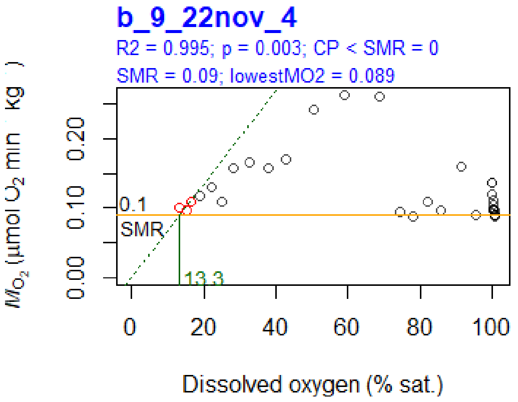
b_9_22nov_4
R2 = 0.995; p = 0.003; CP < SMR = 0
SMR = 0.09; lowestMO2 = 0.089
0.1
SMR
13.3
Dissolved oxygen (% sat.)

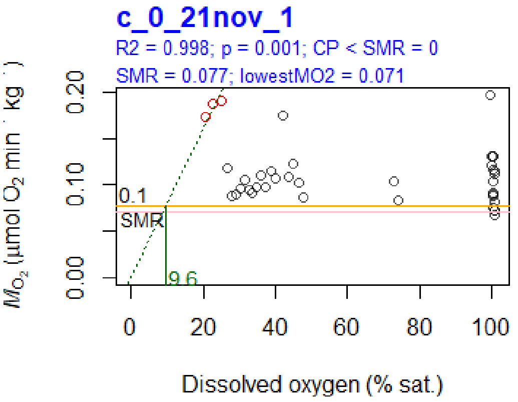
c_0_21nov_1
R2 = 0.998; p = 0.001; CP < SMR = 0
SMR = 0.077; lowestMO2 = 0.071
0.1
SMR
9.6
Dissolved oxygen (% sat.)

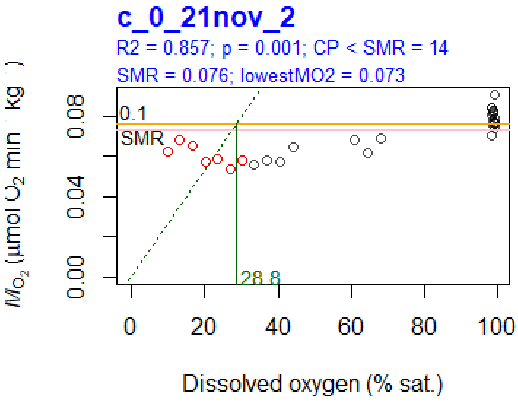
c_0_21nov_2
R2 = 0.857; p = 0.001; CP < SMR = 14
SMR = 0.076; lowestMO2 = 0.073
0.1
SMR
28.8
Dissolved oxygen (% sat.)

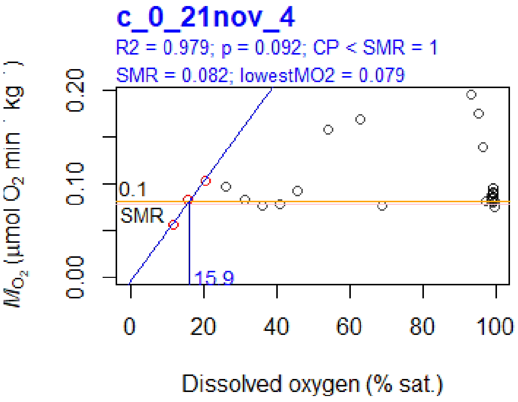
c_0_21nov_4
R2 = 0.979; p = 0.092; CP < SMR = 1
SMR = 0.082; lowestMO2 = 0.079
0.1
SMR
15.9
Dissolved oxygen (% sat.)

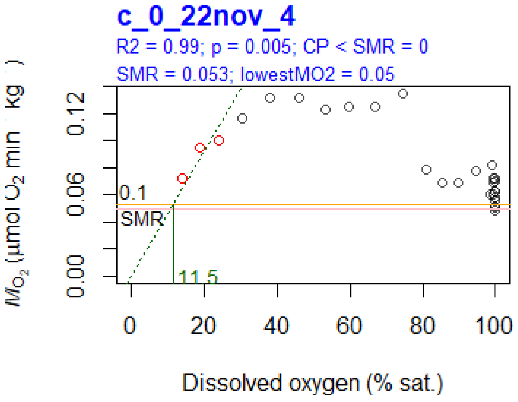
c_0_22nov_4
R2 = 0.99; p = 0.005; CP < SMR = 0
SMR = 0.053; lowestMO2 = 0.05
0.1
SMR
11.5
Dissolved oxygen (% sat.)

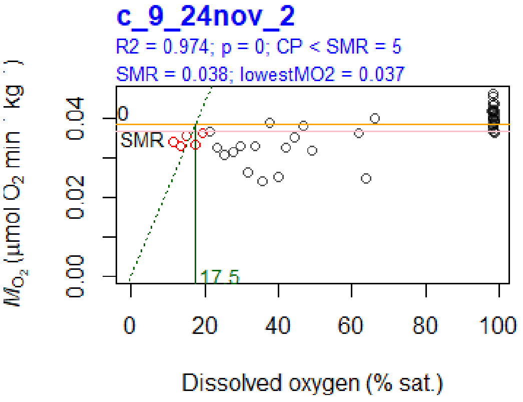
c_9_24nov_2
R2 = 0.974; p = 0; CP < SMR = 5
SMR = 0.038; lowestMO2 = 0.037
0
SMR
17.5
Dissolved oxygen (% sat.)

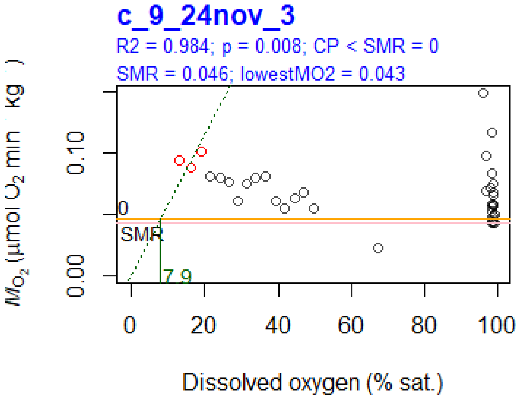
c_9_24nov_3
R2 = 0.984; p = 0.008; CP < SMR = 0
SMR = 0.046; lowestMO2 = 0.043
0
SMR
7.9
Dissolved oxygen (% sat.)

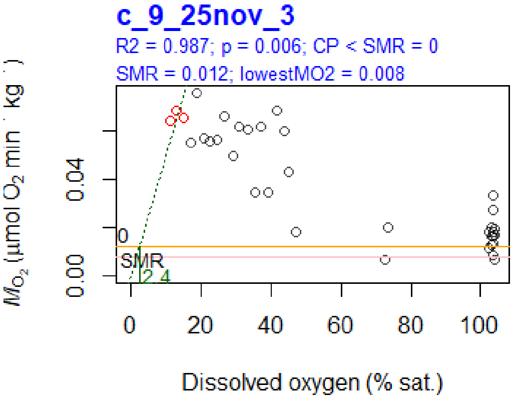
c_9_25nov_3
R2 = 0.987; p = 0.006; CP < SMR = 0
SMR = 0.012; lowestMO2 = 0.008
0
SMR
2.4
Dissolved oxygen (% sat.)

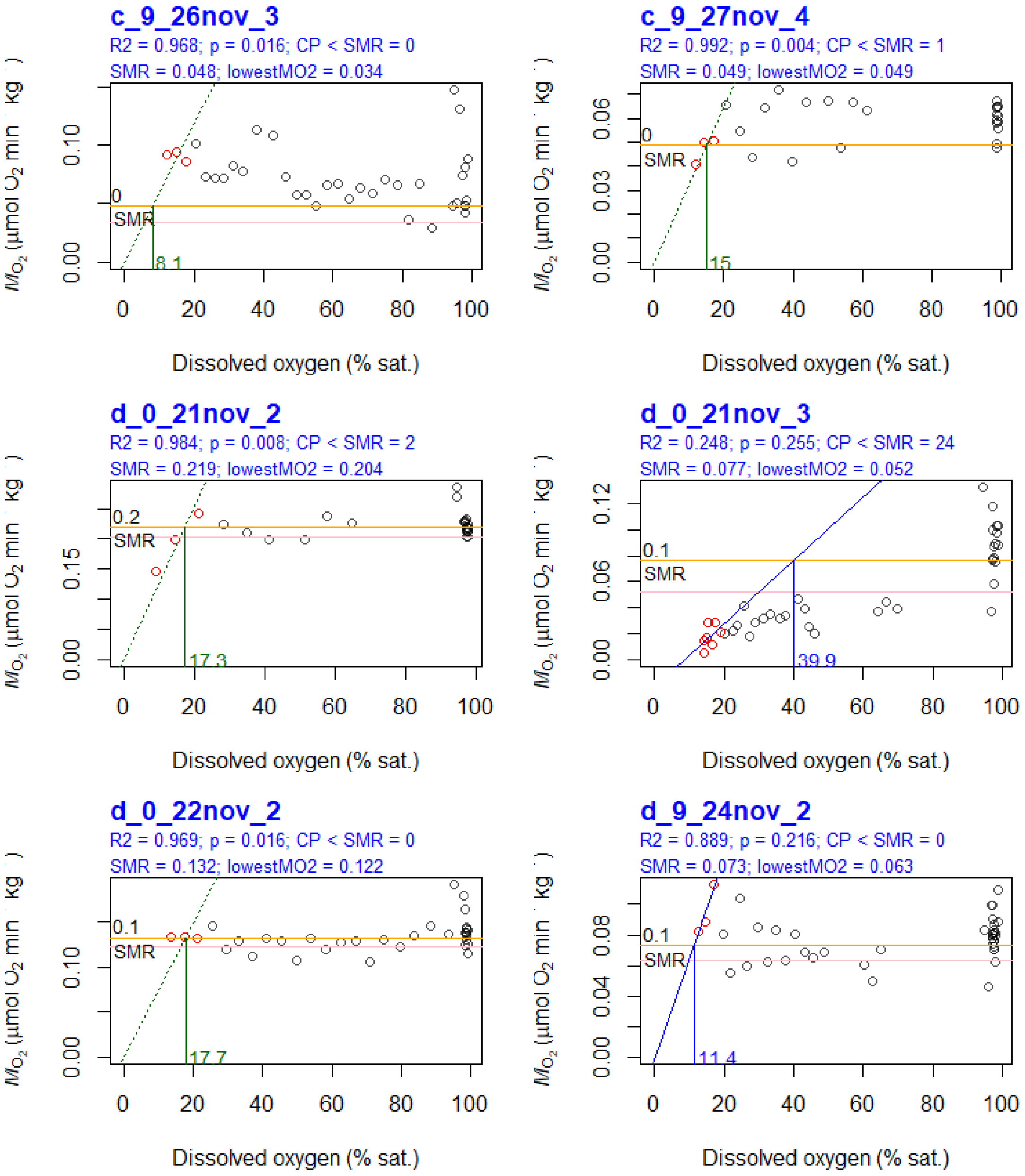

c_9_26nov_3
R2 = 0.968; p = 0.016; CP < SMR = 0
SMR = 0.048; lowestMO2 = 0.034
SMR
8.1
c_9_27nov_4
R2 = 0.992; p = 0.004; CP < SMR = 1
SMR = 0.049; lowestMO2 = 0.049
SMR
15
d_0_21nov_2
R2 = 0.984; p = 0.008; CP < SMR = 2
SMR = 0.219; lowestMO2 = 0.204
SMR
17.3
d_0_21nov_3
R2 = 0.248; p = 0.255; CP < SMR = 24
SMR = 0.077; lowestMO2 = 0.052
SMR
39.9
d_0_22nov_2
R2 = 0.969; p = 0.016; CP < SMR = 0
SMR = 0.132; lowestMO2 = 0.122
SMR
17.7
d_9_24nov_2
R2 = 0.889; p = 0.216; CP < SMR = 0
SMR = 0.073; lowestMO2 = 0.063
SMR
11.4
Dissolved oxygen (% sat.)

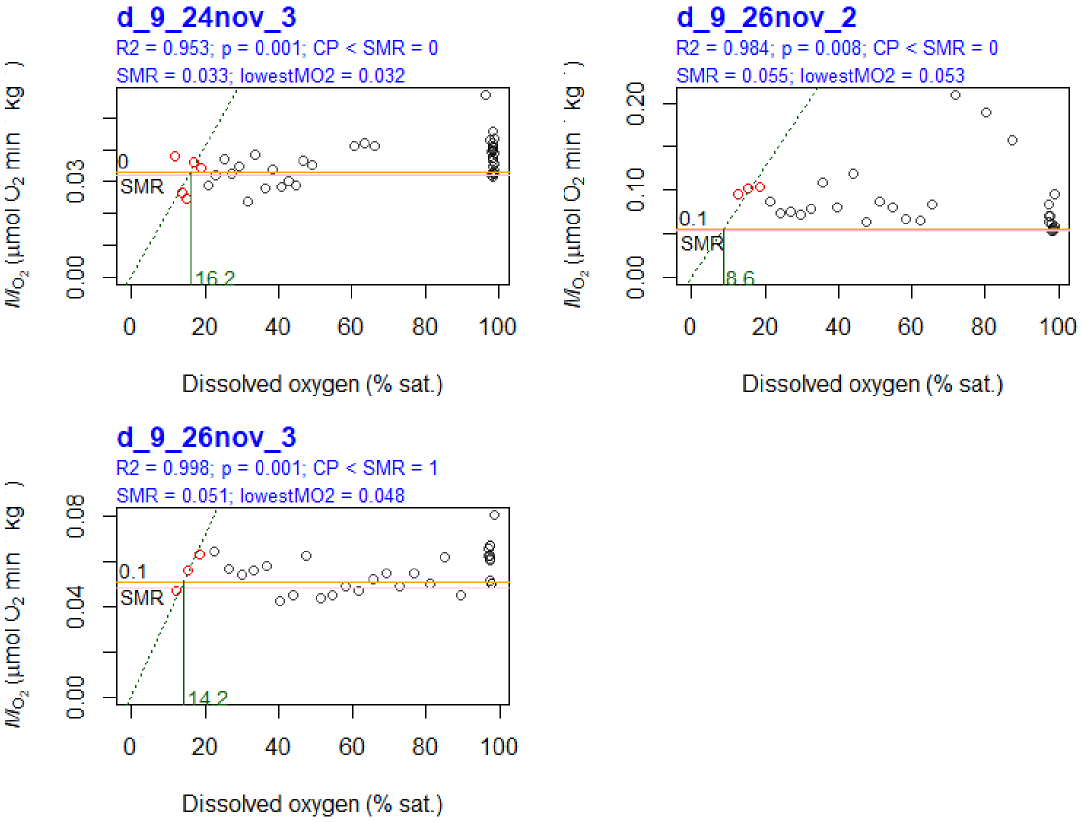

O_2crit numerical rules

We applied a numerical rule to asses if a O2crit was observed. Specifically, we filtered for only cases were at the lowest O_2 value three consecutive MO_2 measures full below our SMR and fifth percentile of the MO2 values observed at dissolved O2 levels > 80%. In the model output these are called nb_mo2_conforming points.

```
## [1] "Based on this rule there are 16 fish with possible O₂crits."
```

Figure S6

Plotting fish that meet our numerical rule for O2crit

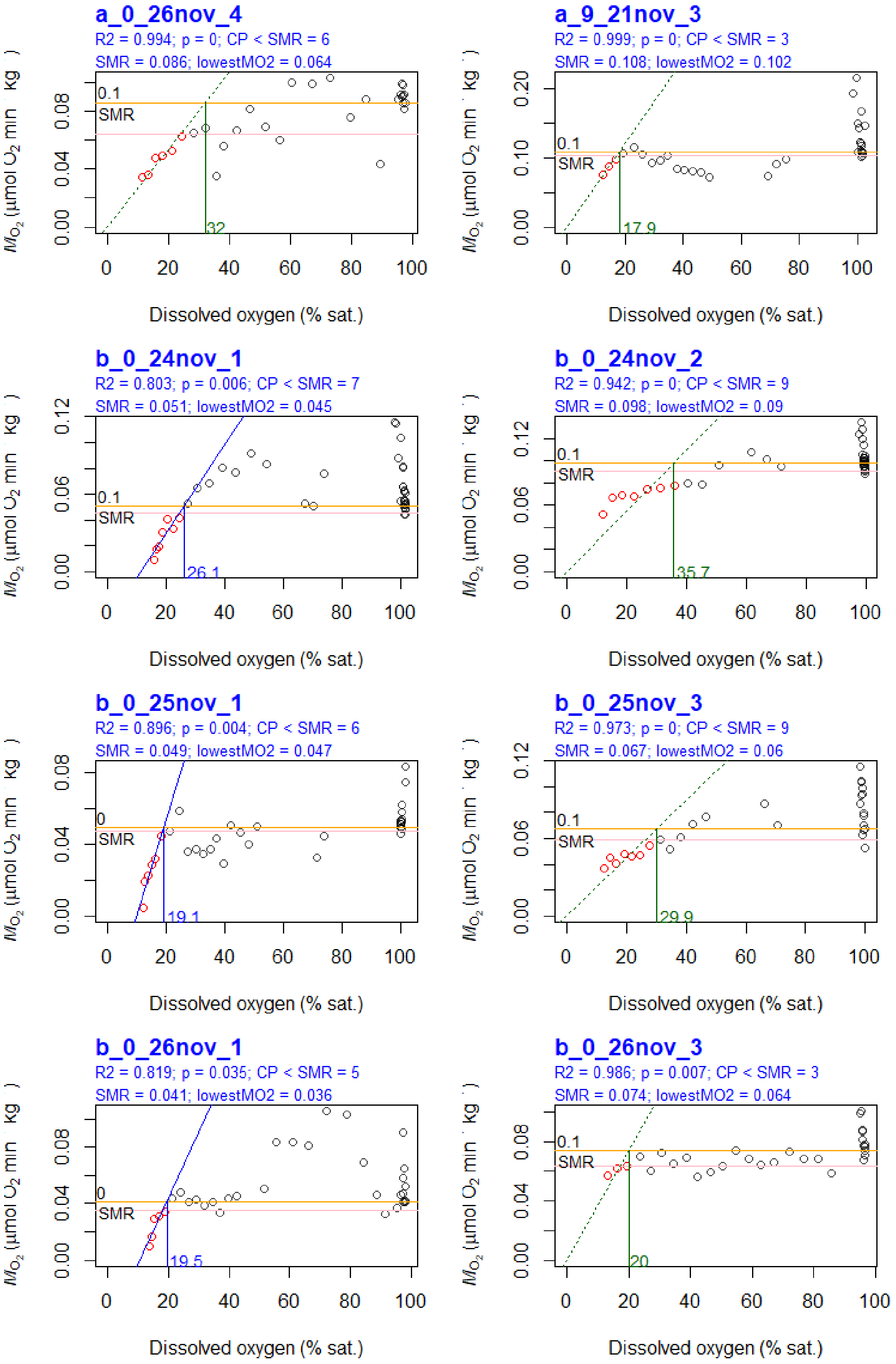
a_0_26nov_4
R2 = 0.994; p = 0; CP < SMR = 6
SMR = 0.086; lowestMO2 = 0.064
32
a_9_21nov_3
R2 = 0.999; p = 0; CP < SMR = 3
SMR = 0.108; lowestMO2 = 0.102
17.9
b_0_24nov_1
R2 = 0.803; p = 0.006; CP < SMR = 7
SMR = 0.051; lowestMO2 = 0.045
26.1
b_0_24nov_2
R2 = 0.942; p = 0; CP < SMR = 9
SMR = 0.098; lowestMO2 = 0.09
35.7
b_0_25nov_1
R2 = 0.896; p = 0.004; CP < SMR = 6
SMR = 0.049; lowestMO2 = 0.047
19.1
b_0_25nov_3
R2 = 0.973; p = 0; CP < SMR = 9
SMR = 0.067; lowestMO2 = 0.06
29.9
b_0_26nov_1
R2 = 0.819; p = 0.035; CP < SMR = 5
SMR = 0.041; lowestMO2 = 0.036
19.5
b_0_26nov_3
R2 = 0.986; p = 0.007; CP < SMR = 3
SMR = 0.074; lowestMO2 = 0.064
20
SMR
Dissolved oxygen (% sat.)

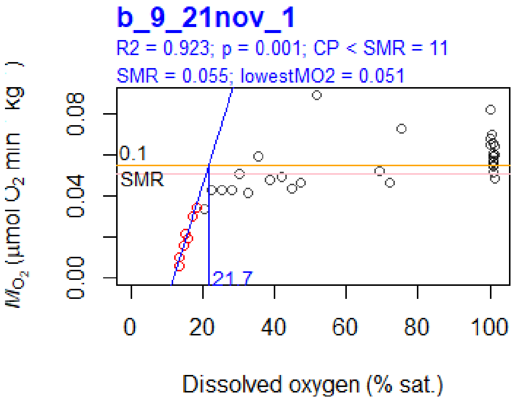
b_9_21nov_1
R2 = 0.923; p = 0.001; CP < SMR = 11
SMR = 0.055; lowestMO2 = 0.051
0.1
SMR
21.7
Dissolved oxygen (% sat.)

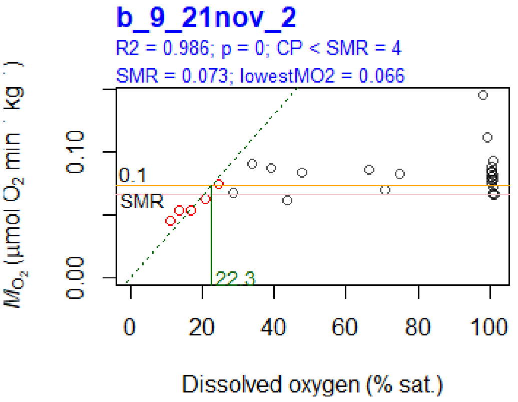
b_9_21nov_2
R2 = 0.986; p = 0; CP < SMR = 4
SMR = 0.073; lowestMO2 = 0.066
0.1
SMR
22.3
Dissolved oxygen (% sat.)

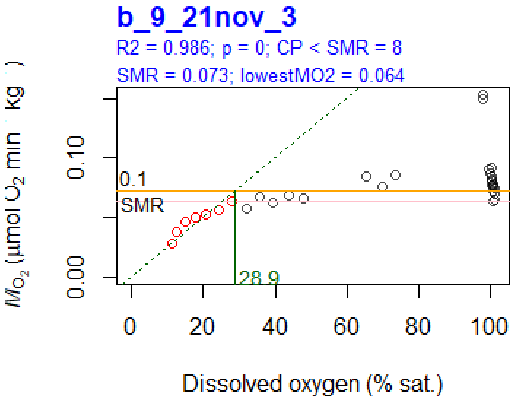
b_9_21nov_3
R2 = 0.986; p = 0; CP < SMR = 8
SMR = 0.073; lowestMO2 = 0.064
0.1
SMR
28.9
Dissolved oxygen (% sat.)

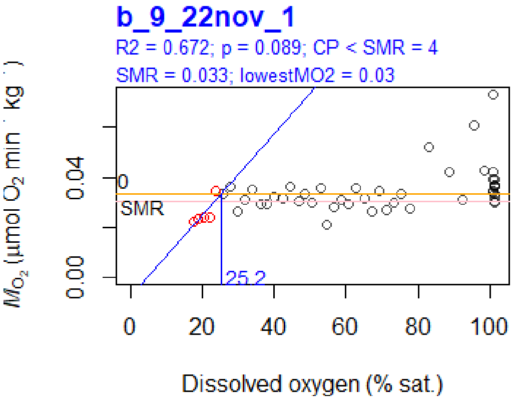
b_9_22nov_1
R2 = 0.672; p = 0.089; CP < SMR = 4
SMR = 0.033; lowestMO2 = 0.03
0
SMR
25.2
Dissolved oxygen (% sat.)

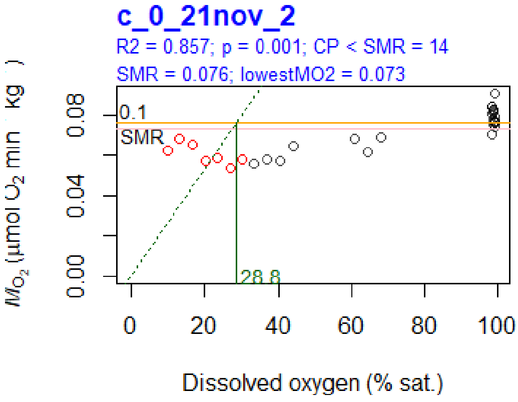
c_0_21nov_2
R2 = 0.857; p = 0.001; CP < SMR = 14
SMR = 0.076; lowestMO2 = 0.073
0.1
SMR
28.8
Dissolved oxygen (% sat.)

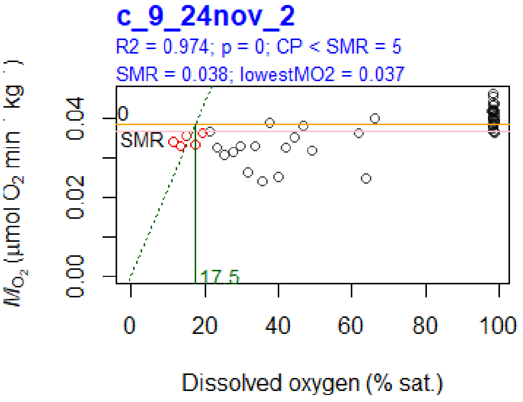
c_9_24nov_2
R2 = 0.974; p = 0; CP < SMR = 5
SMR = 0.038; lowestMO2 = 0.037
0
SMR
17.5
Dissolved oxygen (% sat.)

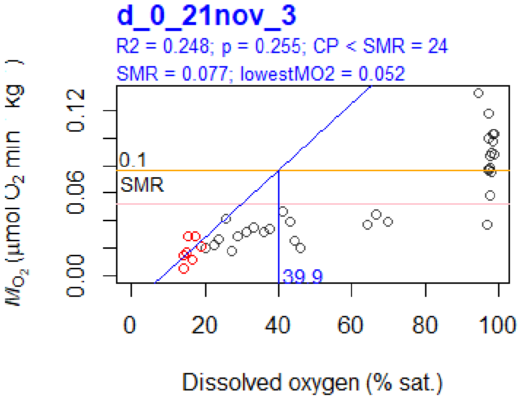
d_0_21nov_3
R2 = 0.248; p = 0.255; CP < SMR = 24
SMR = 0.077; lowestMO2 = 0.052
0.1
SMR
39.9
Dissolved oxygen (% sat.)

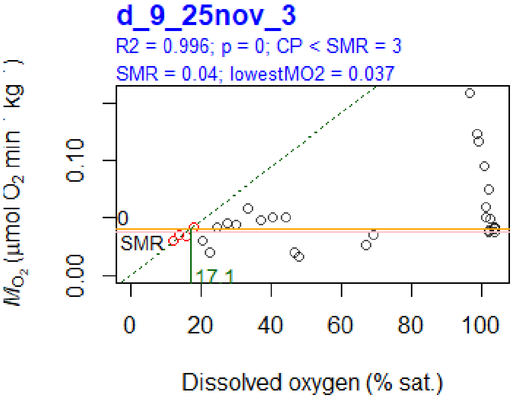
d_9_25nov_3
R2 = 0.996; p = 0; CP < SMR = 3
SMR = 0.04; lowestMO2 = 0.037
0
SMR
17.1
Dissolved oxygen (% sat.)

Figure S6: Representative examples of fish that were best modelled by a 2nd- or 3rd-order polynomial and meet the numerical inclusion criteria. The horizontal solid orange line shows the standard metabolic rate (SMR), the horizontal solid pink line shows thelowestMO2 (as defined by Claireaux and Chabot (2016)), the solid/dashed blue/green slope line shows the O2 regulation failure regression (based on the rule-based linear regression method of Claireaux and Chabot (2016); where the colour and line type indicate the type of regression fitted, through orgin or not), and the solid vertical blue/green line shows the O2crit estimate (intersection of O2 failure regression and SMR).

How many from each salinity treatment

salinity_group	n
0	9
9	7

Visual inspection

We conducted visual inspection of all 58 fish to determine if O2crits were present. We have loaded this as a data frame 💿 **o2crit_check**

For this visual inspection, we grouped fish into “no”, “maybe” or “yes”, based on the certainty at which we observed a O2crits. In the case of yes or maybe, we also estimate dissolved oxygen percentage. All authors did this independently.

Results

Let’s figure out how consistent the categorisation was, and what the majority response was. There were 35 cases with no disagreements (60.34%), 23 cases with one or more disagreements (39.66%).

cat_disagreement	n	percent
0	35	60.34
1	23	39.66

Figure S7

Here’s a heat map to show decisions for each fish

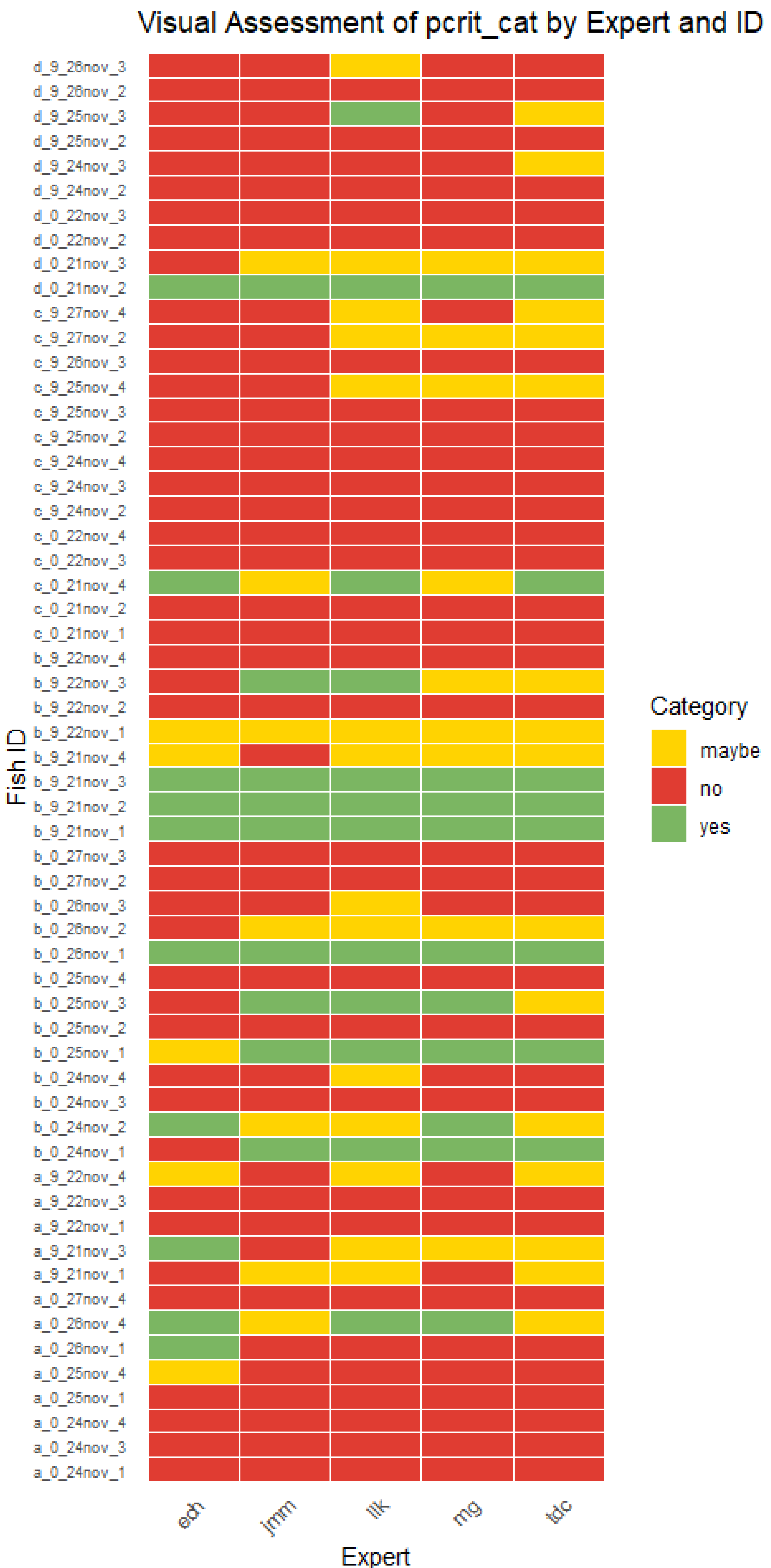

Visual Assessment of pcrit_cat by Expert and ID
Fish ID
d_9_26nov_3
d_9_26nov_2
d_9_25nov_3
d_9_25nov_2
d_9_24nov_3
d_9_24nov_2
d_0_22nov_3
d_0_22nov_2
d_0_21nov_3
d_0_21nov_2
c_9_27nov_4
c_9_27nov_2
c_9_26nov_3
c_9_25nov_4
c_9_25nov_3
c_9_25nov_2
c_9_24nov_4
c_9_24nov_3
c_9_24nov_2
c_0_22nov_4
c_0_22nov_3
c_0_21nov_4
c_0_21nov_2
c_0_21nov_1
b_9_22nov_4
b_9_22nov_3
b_9_22nov_2
b_9_22nov_1
b_9_21nov_4
b_9_21nov_3
b_9_21nov_2
b_9_21nov_1
b_0_27nov_3
b_0_27nov_2
b_0_26nov_3
b_0_26nov_2
b_0_26nov_1
b_0_25nov_4
b_0_25nov_3
b_0_25nov_2
b_0_25nov_1
b_0_24nov_4
b_0_24nov_3
b_0_24nov_2
b_0_24nov_1
a_9_22nov_4
a_9_22nov_3
a_9_22nov_1
a_9_21nov_3
a_9_21nov_1
a_0_27nov_4
a_0_26nov_4
a_0_26nov_1
a_0_25nov_4
a_0_25nov_1
a_0_24nov_4
a_0_24nov_3
a_0_24nov_1
ech
jmm
llk
mg
tdc
Expert
Category
maybe
no
yes

Figure S7: Heatmap showing the visual assessment of critical oxygen saturation (O2crits) category ("no", "maybe" or "yes") for each fish by expert assessor (Elizabeth C. Hoots [ech], Jake M. Martin [jmm], Luis L. Kuchenmülle [llk] Maryane Gradito [mg], Timothy D. Clark [tdc]). Each tile represents one fish-expert pairing, colour-coded by assigned category: no (red, #E03C32), maybe (yellow, #FFD301), and yes (green, #7BB662).

Expert deviation from the majority assessment ranged from 22.41 to 6.90%.

expert	deviations	deviation_rate
ech	13	22.41
jmm	8	13.79
llk	6	10.34
tdc	5	8.62
mg	4	6.90

Let's make a list of those that had majority assigned as yes for an O2crit. There was 10 total.

```
## [1] "Based on the majority visual assessment 10 fish have possible
O₂crit."
```

O_2crit numbers

We will now check to see which fish have O2crit based on the numerical and visual inspections.

There are eight fish that both numerically and visually were determined to have a O2crit. Two fish with a visually confirmed O2crit, that did not meet the numerical criteria. Eight fish meet the numerical criteria, but did not pass the visual inspection.

```
## $Shared
## [1] "a_0_26nov_4" "b_0_24nov_1" "b_0_25nov_1" "b_0_25nov_3" "b_0_26nov_1"
## [6] "b_9_21nov_1" "b_9_21nov_2" "b_9_21nov_3"
##
## $Unique_to_visual
## [1] "c_0_21nov_4" "d_0_21nov_2"
##
## $Unique_to_numerical
## [1] "a_9_21nov_3" "b_0_24nov_2" "b_0_26nov_3" "b_9_22nov_1" "c_0_21nov_2"
## [6] "c_9_24nov_2" "d_0_21nov_3" "d_9_25nov_3"
```

How many per salinity treatment

salinity_group	n
0	5
9	3

Results

Looking at mean O2crit estimates.

n_o2crit	mean_o2crit	min_o2crit	max_o2crit	mean_mass	mean_rel_mass	sd_mass	sd_rel_mass
8.00	24.94	19.10	32.00	0.46	0.85	0.18	0.34

n	mean_min_do	sd_min_do	min_min_do
50	13.12	3.23	8.82

salinity_group	n_o2crit	mean_o2crit	min_o2crit	max_o2crit	mean_mass
0	5	25.32	19.1	32.0	0.3540000
9	3	24.30	21.7	28.9	0.6466667

Figure S8

These are the 8 fish that meet numerical criteria and passed visual inspection.

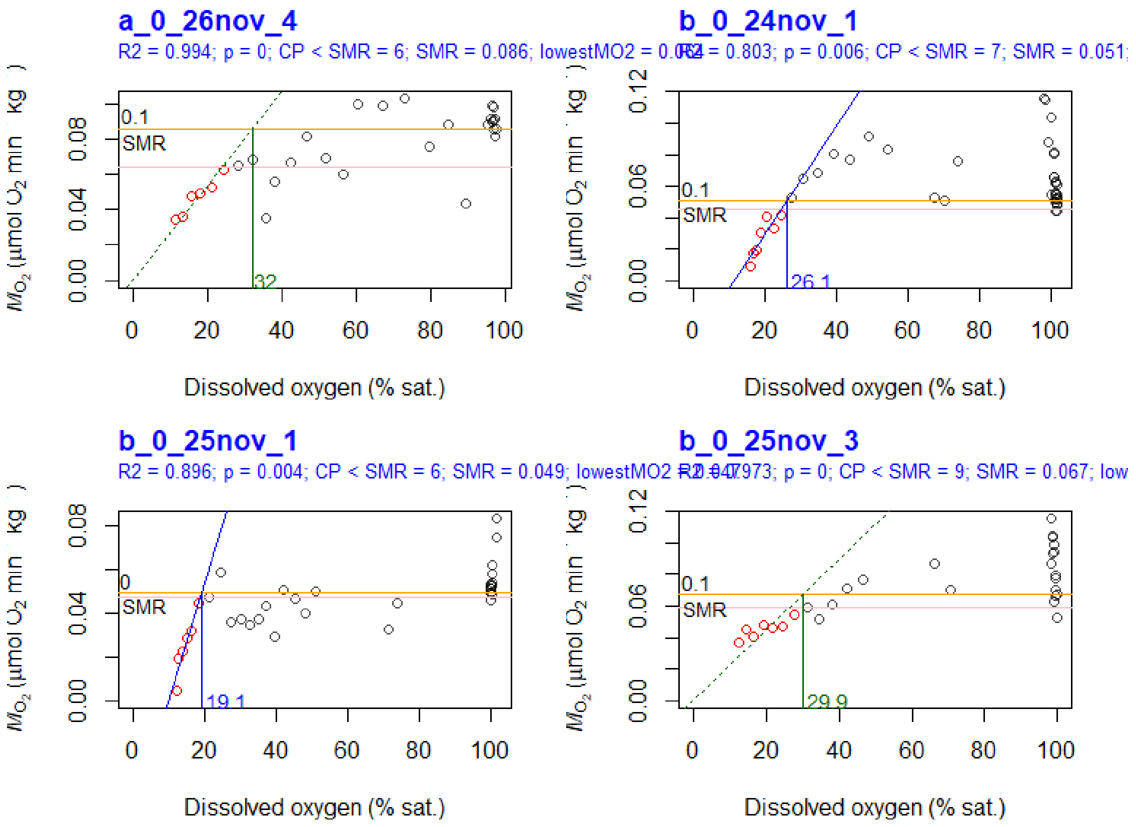

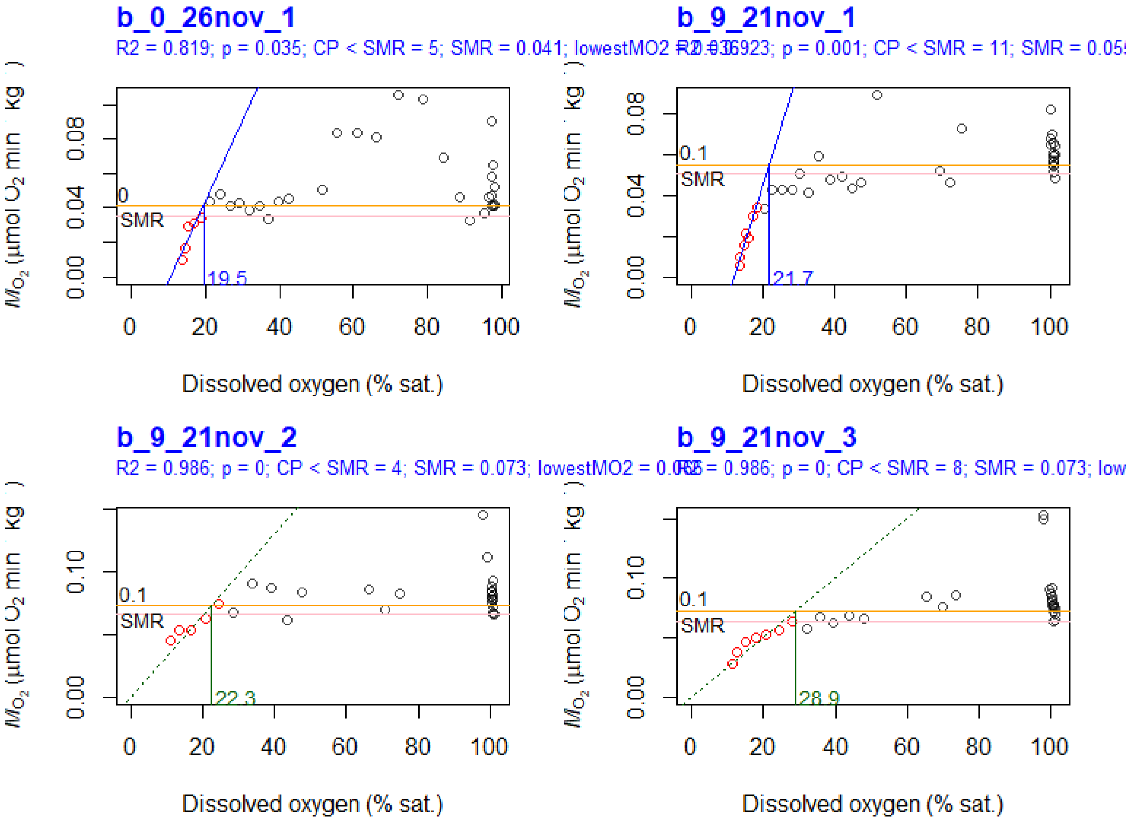

Figure S8: Representative examples of fish that were best modelled by a 2nd- or 3rd-order polynomial and meet the numerical inclusion criteria. The horizontal solid orange line shows the standard metabolic rate (SMR), the horizontal solid pink line shows thelowestMO2 (as defined by Claireaux and Chabot (2016)), the solid/dashed blue/green slope line shows the O2 regulation failure regression (based on the rule-based linear regression method of Claireaux and Chabot (2016); where the colour and line type indicate the type of regression fitted, through orgin or not), and the solid vertical blue/green line shows the O2crit estimate (intersection of O2 failure regression and SMR).

Pulling model method and lethal points

Figure S9

Making a O2crit plot for each of the eight fish that past visual and numerical assessment on the same axis.

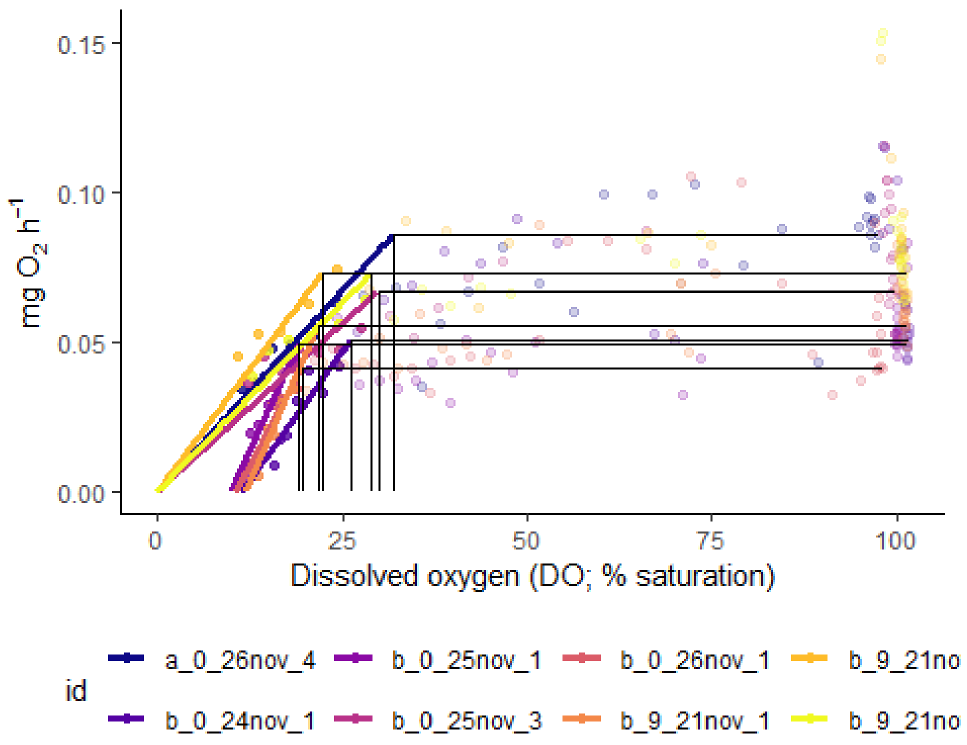

Figure S9: Oxygen consumption rate ($\dot{M}O_2$; mg^{-1} O_2 h^{-1}) and ambient dissolved oxygen (DO; % saturation) plotted for each fish that passed both past visual and numerical assessment for O2crit. The horizontal dashed lines show SMR, the solid sloped lines represent the linear best fit for $\dot{M}O$ values below regulatory failure. The vertical soild lines represent O2crit (the intersection of the linear best fit for $\dot{M}O$ values below regulatory failure and SMR).

Figure 2

Let's make some example plots for each major types of O2-MO2 relationships (i.e. 0th-, 1st-, and $2^{nd}/3^{rd}$- order polynomial).

First an example of a 0th-order polynomial fit (c_9_24nov_4).

Second an example of a 1st-order polynomial best fit (d_9_25nov_3).

Last an example of a fish best fit with a higher order polynomial (2nd-order).

Let's combined them in one plot

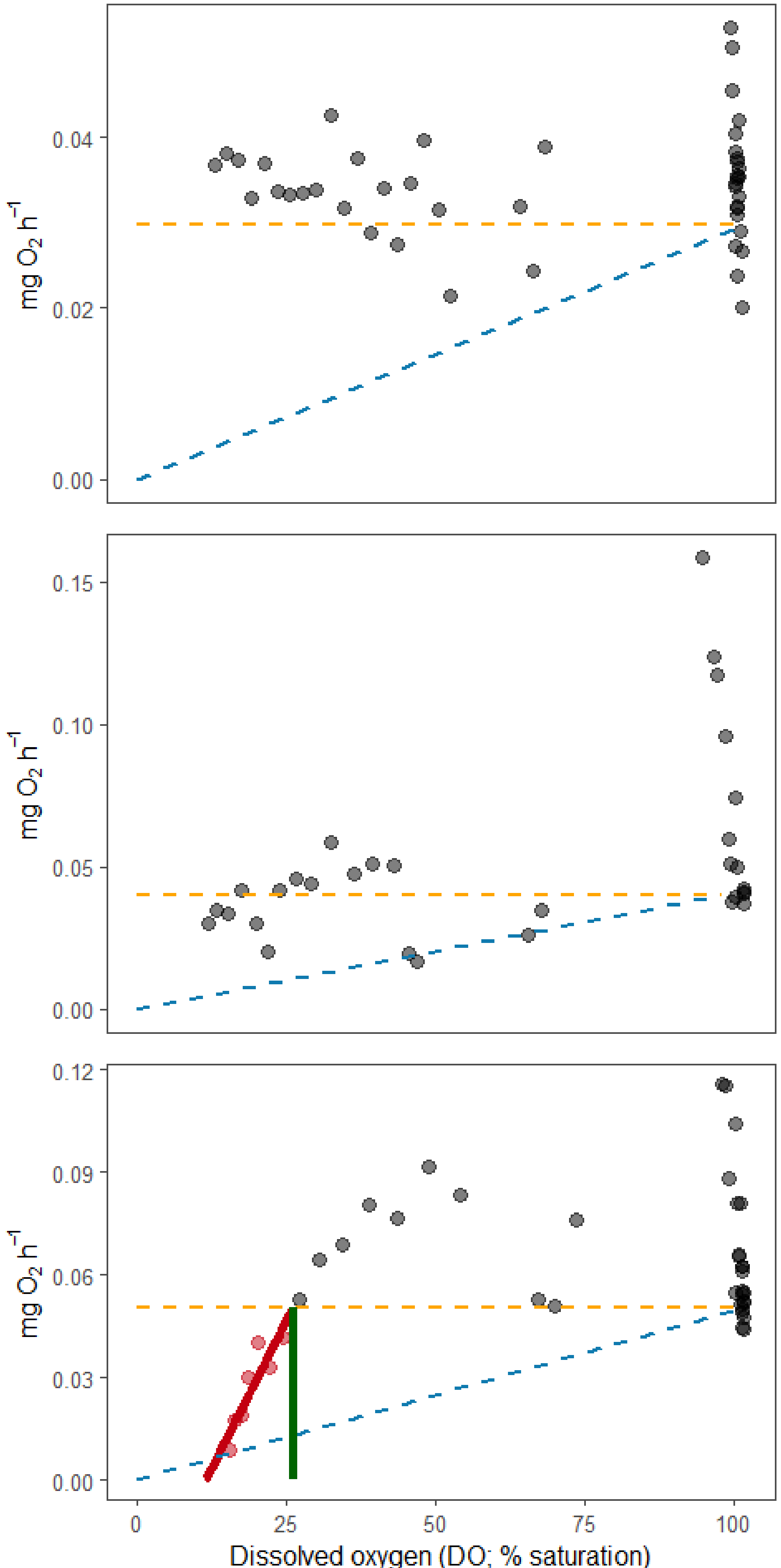

mg O2 h-1
0.00
0.02
0.04
0.00
0.05
0.10
0.15
0.00
0.03
0.06
0.09
0.12
0
25
50
75
100
Dissolved oxygen (DO; % saturation)

Figure 2: Figure 2. Representative examples of fish that were best modelled by a 0th-order polynomial (A), 1st-order polynomial (B), and 2nd-order polynomial (C). The orange dashed line shows the SMR, while the blue dashed line shows the theoretical oxyconfroming regression. For plot (C), the red sloped line shows the O2 regulation failure regression (based on the rule-based linear regression method of Claireaux and Chabot, 2016), and the solid horizontal green line shows the O2crit estimate (intersection of O2 failure regression and SMR).

Comapring to past data

Here, we have recreated a similar plot to that presented in Urbina, Glover, and Forster (2012)[1] and have extracted the mean level data from Figure 1a using the metaDigitise package in R[3]. This data is called urbina_et_al_2012. This allows us to compare the differences in Mo2 and the relationship between Mo2 and O2.

First making a binned data frame to match Urbina, Glover, and Forster (2012) as closely as possible.

Figure 3

Now the plot with our mean data and the mean data from Urbina, Glover, and Forster (2012)[1]

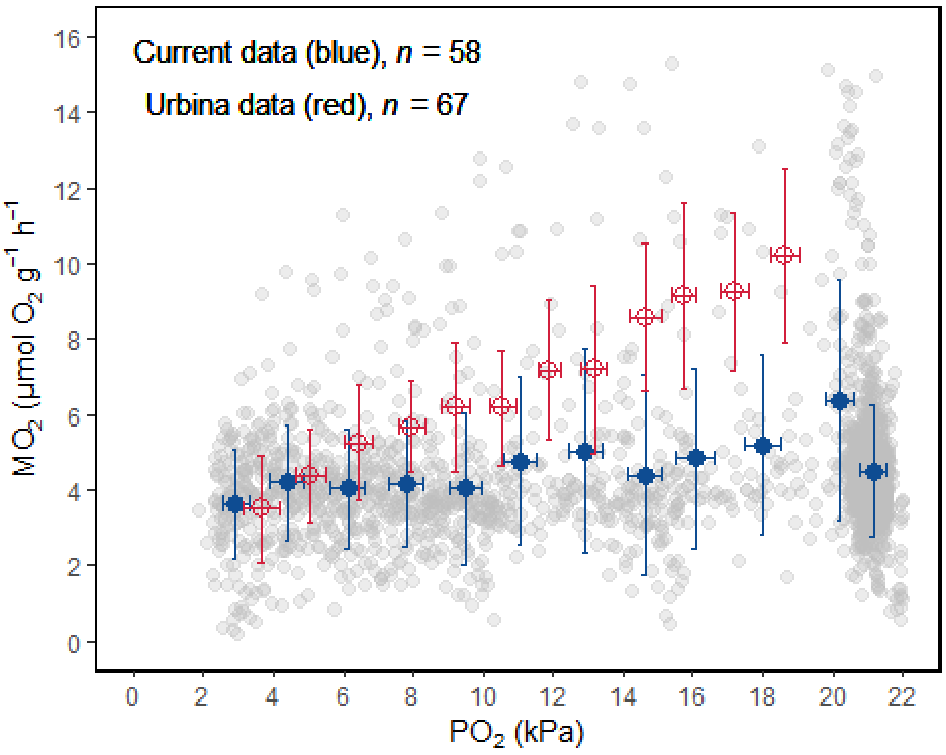

Figure 3: Mean and standard error of metabolic rate (MO2 μmol O2 g^{-1} h^{-1}) and oxygen concentration (PO2 kPa) using 12 evenly spaced bins over the O2 range of observed data

(blue filled dots). Compared against the mean and standard error reported in Urbina (2012)[1] (red open dots). The grey dots are the raw observed data form the present study.

References

[1] Claireaux, G. and Chabot, D. (2016) Responses by fishes to environmental hypoxia: integration through Fry's concept of aerobic metabolic scope. Journal of Fish Biology https://doi.org/10.1111/jfb.12833

[2] Urbina MA, Glover CN, and Forster ME, (2012) A novel oxyconforming response in the freshwater fish *Galaxias maculatus*. Comparative Biochemistry and Physiology Part A: Molecular & Integrative Physiology. https://doi.org/10.1016/j.cbpa.2011.11.011

[3] Pick JL, Nakagawa S, and Noble DWA (2018) Reproducible, flexible and high-throughput data extraction from primary literature: The metaDigitise r package. https://doi.org/10.1111/2041-210X.13118

Session information

```
##
## To cite R in publications use:
##
##   R Core Team (2023). R: A language and environment for statistical
##   computing. R Foundation for Statistical Computing, Vienna, Austria.
##   URL https://www.R-project.org/.
##
## A BibTeX entry for LaTeX users is
##
##   @Manual{,
##     title = {R: A Language and Environment for Statistical Computing},
##     author = {{R Core Team}},
##     organization = {R Foundation for Statistical Computing},
##     address = {Vienna, Austria},
##     year = {2023},
##     url = {https://www.R-project.org/},
##   }
##
## We have invested a lot of time and effort in creating R, please cite it
## when using it for data analysis. See also 'citation("pkgname")' for
## citing R packages.
```

Here is a detailed list of the session information

```
## ─ Session info
───────────────────────────────────────────────────────────────
##  setting  value
##  version  R version 4.2.3 (2023-03-15 ucrt)
##  os       Windows 10 x64 (build 19044)
```

```
##  system   x86_64, mingw32
##  ui       RTerm
##  language (EN)
##  collate  English_Australia.utf8
##  ctype    English_Australia.utf8
##  tz       Australia/Sydney
##  date     2026-02-13
##  pandoc   3.1.1 @ C:/Program
Files/RStudio/resources/app/bin/quarto/bin/tools/ (via rmarkdown)
##
## ─ Packages
─────────────────────────────────────────────────────────────────
##  package           * version  date (UTC) lib source
##  abind               1.4-5    2016-07-21 [1] CRAN (R 4.2.0)
##  arrayhelpers        1.1-0    2020-02-04 [1] CRAN (R 4.2.3)
##  askpass             1.2.0    2023-09-03 [1] CRAN (R 4.2.3)
##  backports           1.4.1    2021-12-13 [1] CRAN (R 4.2.0)
##  bayesplot         * 1.11.1   2024-02-15 [1] CRAN (R 4.2.3)
##  bitops              1.0-7    2021-04-24 [1] CRAN (R 4.2.0)
##  bridgesampling      1.1-2    2021-04-16 [1] CRAN (R 4.2.3)
##  brms              * 2.21.0   2024-03-20 [1] CRAN (R 4.2.3)
##  Brobdingnag         1.2-9    2022-10-19 [1] CRAN (R 4.2.3)
##  broom               1.0.4    2023-03-11 [1] CRAN (R 4.2.3)
##  broom.helpers       1.15.0   2024-04-05 [1] CRAN (R 4.2.3)
##  broom.mixed       * 0.2.9.5  2024-04-01 [1] CRAN (R 4.2.3)
##  cachem              1.0.7    2023-02-24 [1] CRAN (R 4.2.3)
##  caTools             1.18.2   2021-03-28 [1] CRAN (R 4.2.3)
##  cellranger          1.1.0    2016-07-27 [1] CRAN (R 4.2.3)
##  checkmate           2.3.1    2023-12-04 [1] CRAN (R 4.2.3)
##  cli                 3.6.4    2025-02-13 [1] CRAN (R 4.2.3)
##  coda                0.19-4.1 2024-01-31 [1] CRAN (R 4.2.3)
##  codetools           0.2-19   2023-02-01 [2] CRAN (R 4.2.3)
##  colorspace          2.1-0    2023-01-23 [1] CRAN (R 4.2.3)
##  crayon              1.5.3    2024-06-20 [1] CRAN (R 4.2.3)
##  crul                1.4.2    2024-04-09 [1] CRAN (R 4.2.3)
##  curl                5.2.1    2024-03-01 [1] CRAN (R 4.2.3)
##  data.table        * 1.17.0   2025-02-22 [1] CRAN (R 4.2.3)
##  DEoptimR            1.1-3    2023-10-07 [1] CRAN (R 4.2.3)
##  devtools          * 2.4.5    2022-10-11 [1] CRAN (R 4.2.3)
##  digest              0.6.35   2024-03-11 [1] CRAN (R 4.2.3)
##  distributional      0.4.0    2024-02-07 [1] CRAN (R 4.2.3)
##  doParallel          1.0.17   2022-02-07 [1] CRAN (R 4.2.3)
##  dplyr             * 1.1.4    2023-11-17 [1] CRAN (R 4.2.3)
##  ellipsis            0.3.2    2021-04-29 [1] CRAN (R 4.2.3)
##  emmeans           * 1.11.0   2025-03-20 [1] CRAN (R 4.2.3)
##  estimability        1.5.1    2024-05-12 [1] CRAN (R 4.2.3)
##  evaluate            0.24.0   2024-06-10 [1] CRAN (R 4.2.3)
##  fansi               1.0.4    2023-01-22 [1] CRAN (R 4.2.3)
##  farver              2.1.1    2022-07-06 [1] CRAN (R 4.2.3)
##  fastmap             1.1.1    2023-02-24 [1] CRAN (R 4.2.3)
```

```
##  flextable         * 0.9.6    2024-05-05 [1] CRAN (R 4.2.3)
##  fontBitstreamVera   0.1.1    2017-02-01 [1] CRAN (R 4.2.3)
##  fontLiberation      0.1.0    2016-10-15 [1] CRAN (R 4.2.3)
##  fontquiver          0.2.1    2017-02-01 [1] CRAN (R 4.2.3)
##  forcats           * 1.0.0    2023-01-29 [1] CRAN (R 4.2.3)
##  foreach             1.5.2    2022-02-02 [1] CRAN (R 4.2.3)
##  fs                  1.6.4    2024-04-25 [1] CRAN (R 4.2.3)
##  furrr               0.3.1    2022-08-15 [1] CRAN (R 4.2.3)
##  future            * 1.33.2   2024-03-26 [1] CRAN (R 4.2.3)
##  gdtools             0.3.7    2024-03-05 [1] CRAN (R 4.2.3)
##  generics            0.1.3    2022-07-05 [1] CRAN (R 4.2.3)
##  gfonts              0.2.0    2023-01-08 [1] CRAN (R 4.2.3)
##  ggdist              3.3.2    2024-03-05 [1] CRAN (R 4.2.3)
##  ggplot2           * 3.5.2    2025-04-09 [1] CRAN (R 4.2.3)
##  ggthemes          * 5.1.0    2024-02-10 [1] CRAN (R 4.2.3)
##  globals             0.16.3   2024-03-08 [1] CRAN (R 4.2.3)
##  glue                1.8.0    2024-09-30 [1] CRAN (R 4.2.3)
##  gridExtra         * 2.3      2017-09-09 [1] CRAN (R 4.2.3)
##  gsw                 1.2-0    2024-08-19 [1] CRAN (R 4.2.3)
##  gt                * 0.11.0   2024-07-09 [1] CRAN (R 4.2.3)
##  gtable              0.3.5    2024-04-22 [1] CRAN (R 4.2.3)
##  gtsummary         * 1.7.2    2023-07-15 [1] CRAN (R 4.2.3)
##  here              * 1.0.1    2020-12-13 [1] CRAN (R 4.2.3)
##  hms               * 1.1.3    2023-03-21 [1] CRAN (R 4.2.3)
##  htmltools           0.5.8.1  2024-04-04 [1] CRAN (R 4.2.3)
##  htmlwidgets         1.6.4    2023-12-06 [1] CRAN (R 4.2.3)
##  httpcode            0.3.0    2020-04-10 [1] CRAN (R 4.2.3)
##  httpuv              1.6.9    2023-02-14 [1] CRAN (R 4.2.3)
##  httr                1.4.7    2023-08-15 [1] CRAN (R 4.2.3)
##  inline              0.3.19   2021-05-31 [1] CRAN (R 4.2.3)
##  insight             1.1.0    2025-03-01 [1] CRAN (R 4.2.3)
##  iterators           1.0.14   2022-02-05 [1] CRAN (R 4.2.3)
##  janitor           * 2.2.0    2023-02-02 [1] CRAN (R 4.2.3)
##  jsonlite            1.8.8    2023-12-04 [1] CRAN (R 4.2.3)
##  knitr               1.42     2023-01-25 [1] CRAN (R 4.2.3)
##  labeling            0.4.3    2023-08-29 [1] CRAN (R 4.2.3)
##  later               1.3.0    2021-08-18 [1] CRAN (R 4.2.3)
##  lattice             0.20-45  2021-09-22 [2] CRAN (R 4.2.3)
##  lazyeval            0.2.2    2019-03-15 [1] CRAN (R 4.2.3)
##  lifecycle           1.0.4    2023-11-07 [1] CRAN (R 4.2.3)
##  listenv             0.9.1    2024-01-29 [1] CRAN (R 4.2.3)
##  loo                 2.8.0    2024-07-03 [1] CRAN (R 4.2.3)
##  lubridate         * 1.9.2    2023-02-10 [1] CRAN (R 4.2.3)
##  magrittr            2.0.3    2022-03-30 [1] CRAN (R 4.2.3)
##  marginaleffects   * 0.21.0   2024-06-14 [1] CRAN (R 4.2.3)
##  MASS                7.3-58.2 2023-01-23 [2] CRAN (R 4.2.3)
##  Matrix              1.5-3    2022-11-11 [2] CRAN (R 4.2.3)
##  matrixStats         1.3.0    2024-04-11 [1] CRAN (R 4.2.3)
##  mclust            * 6.1.1    2024-04-29 [1] CRAN (R 4.2.3)
##  memoise             2.0.1    2021-11-26 [1] CRAN (R 4.2.3)
```

```
##   mime                 0.12     2021-09-28 [1] CRAN (R 4.2.0)
##   miniUI               0.1.1.1  2018-05-18 [1] CRAN (R 4.2.3)
##   multcomp             1.4-25   2023-06-20 [1] CRAN (R 4.2.3)
##   munsell              0.5.1    2024-04-01 [1] CRAN (R 4.2.3)
##   mvtnorm              1.2-5    2024-05-21 [1] CRAN (R 4.2.3)
##   nlme                 3.1-162  2023-01-31 [2] CRAN (R 4.2.3)
##   oce                  1.8-3    2024-08-17 [1] CRAN (R 4.2.3)
##   officer            * 0.6.6    2024-05-05 [1] CRAN (R 4.2.3)
##   opdisDownsampling    1.0.1    2024-04-15 [1] CRAN (R 4.2.3)
##   openssl              2.2.0    2024-05-16 [1] CRAN (R 4.2.3)
##   pacman               0.5.1    2019-03-11 [1] CRAN (R 4.2.3)
##   parallelly           1.37.1   2024-02-29 [1] CRAN (R 4.2.3)
##   pbmcapply            1.5.1    2022-04-28 [1] CRAN (R 4.2.0)
##   performance        * 0.12.0   2024-06-08 [1] CRAN (R 4.2.3)
##   pillar               1.9.0    2023-03-22 [1] CRAN (R 4.2.3)
##   pkgbuild             1.4.4    2024-03-17 [1] CRAN (R 4.2.3)
##   pkgconfig            2.0.3    2019-09-22 [1] CRAN (R 4.2.3)
##   pkgload              1.3.4    2024-01-16 [1] CRAN (R 4.2.3)
##   plotly             * 4.10.4   2024-01-13 [1] CRAN (R 4.2.3)
##   plyr                 1.8.8    2022-11-11 [1] CRAN (R 4.2.3)
##   posterior            1.6.0    2024-07-03 [1] CRAN (R 4.2.3)
##   pracma               2.4.4    2023-11-10 [1] CRAN (R 4.2.3)
##   profvis              0.3.8    2023-05-02 [1] CRAN (R 4.2.3)
##   promises             1.2.0.1  2021-02-11 [1] CRAN (R 4.2.3)
##   purrr              * 1.0.1    2023-01-10 [1] CRAN (R 4.2.3)
##   qqconf               1.3.2    2023-04-14 [1] CRAN (R 4.2.3)
##   qqplotr            * 0.0.6    2023-01-25 [1] CRAN (R 4.2.3)
##   QuickJSR             1.3.0    2024-07-08 [1] CRAN (R 4.2.3)
##   R6                   2.5.1    2021-08-19 [1] CRAN (R 4.2.3)
##   ragg                 1.2.5    2023-01-12 [1] CRAN (R 4.2.3)
##   Rcpp               * 1.0.10   2023-01-22 [1] CRAN (R 4.2.3)
##   RcppParallel         5.1.8    2024-07-06 [1] CRAN (R 4.2.3)
##   readr              * 2.1.4    2023-02-10 [1] CRAN (R 4.2.3)
##   readxl             * 1.4.3    2023-07-06 [1] CRAN (R 4.2.3)
##   remotes              2.5.0    2024-03-17 [1] CRAN (R 4.2.3)
##   reshape2             1.4.4    2020-04-09 [1] CRAN (R 4.2.3)
##   respirometry       * 2.0.0    2024-07-18 [1] CRAN (R 4.2.3)
##   rlang                1.1.5    2025-01-17 [1] CRAN (R 4.2.3)
##   rmarkdown            2.21     2023-03-26 [1] CRAN (R 4.2.3)
##   robustbase           0.99-4-1 2024-09-27 [1] CRAN (R 4.2.3)
##   rprojroot            2.0.4    2023-11-05 [1] CRAN (R 4.2.3)
##   rstan              * 2.32.6   2024-03-05 [1] CRAN (R 4.2.3)
##   rstantools           2.4.0    2024-01-31 [1] CRAN (R 4.2.3)
##   rstudioapi           0.14     2022-08-22 [1] CRAN (R 4.2.3)
##   sandwich             3.1-0    2023-12-11 [1] CRAN (R 4.2.3)
##   scales               1.3.0    2023-11-28 [1] CRAN (R 4.2.3)
##   sessioninfo          1.2.2    2021-12-06 [1] CRAN (R 4.2.3)
##   shiny                1.8.1.1  2024-04-02 [1] CRAN (R 4.2.3)
##   snakecase            0.11.1   2023-08-27 [1] CRAN (R 4.2.3)
##   StanHeaders        * 2.32.9   2024-05-29 [1] CRAN (R 4.2.3)
```

```
##  stringi             1.7.12   2023-01-11 [1] CRAN (R 4.2.2)
##  stringr           * 1.5.1    2023-11-14 [1] CRAN (R 4.2.3)
##  survival            3.5-3    2023-02-12 [2] CRAN (R 4.2.3)
##  svUnit              1.0.6    2021-04-19 [1] CRAN (R 4.2.3)
##  systemfonts         1.0.4    2022-02-11 [1] CRAN (R 4.2.3)
##  tensorA             0.36.2.1 2023-12-13 [1] CRAN (R 4.2.3)
##  textshaping         0.3.6    2021-10-13 [1] CRAN (R 4.2.3)
##  TH.data             1.1-2    2023-04-17 [1] CRAN (R 4.2.3)
##  tibble            * 3.2.1    2023-03-20 [1] CRAN (R 4.2.3)
##  tidybayes         * 3.0.6    2023-08-12 [1] CRAN (R 4.2.3)
##  tidyr             * 1.3.0    2023-01-24 [1] CRAN (R 4.2.3)
##  tidyselect          1.2.1    2024-03-11 [1] CRAN (R 4.2.3)
##  tidyverse         * 2.0.0    2023-02-22 [1] CRAN (R 4.2.3)
##  timechange          0.2.0    2023-01-11 [1] CRAN (R 4.2.3)
##  twosamples          2.0.1    2023-06-23 [1] CRAN (R 4.2.3)
##  tzdb                0.3.0    2022-03-28 [1] CRAN (R 4.2.3)
##  urlchecker          1.0.1    2021-11-30 [1] CRAN (R 4.2.3)
##  usethis           * 2.2.3    2024-02-19 [1] CRAN (R 4.2.3)
##  utf8                1.2.3    2023-01-31 [1] CRAN (R 4.2.3)
##  uuid                1.2-0    2024-01-14 [1] CRAN (R 4.2.3)
##  V8                  4.4.2    2024-02-15 [1] CRAN (R 4.2.3)
##  vctrs               0.6.5    2023-12-01 [1] CRAN (R 4.2.3)
##  viridisLite         0.4.2    2023-05-02 [1] CRAN (R 4.2.3)
##  withr               3.0.0    2024-01-16 [1] CRAN (R 4.2.3)
##  xfun                0.38     2023-03-24 [1] CRAN (R 4.2.3)
##  xml2                1.3.6    2023-12-04 [1] CRAN (R 4.2.3)
##  xtable              1.8-4    2019-04-21 [1] CRAN (R 4.2.3)
##  yaml                2.3.7    2023-01-23 [1] CRAN (R 4.2.3)
##  zip                 2.3.1    2024-01-27 [1] CRAN (R 4.2.3)
##  zoo                 1.8-12   2023-04-13 [1] CRAN (R 4.2.3)
##
##  [1] C:/R
##  [2] C:/Program Files/R/R-4.2.3/library
##
##
────────────────────────────────────────────────────────────────────────────
─
```
